# Earlier diagnosis of lung cancer in a randomised trial of an autoantibody blood test followed by imaging

**DOI:** 10.1183/13993003.00670-2020

**Published:** 2021-01-14

**Authors:** Frank M. Sullivan, Frances S. Mair, William Anderson, Pauline Armory, Andrew Briggs, Cindy Chew, Alistair Dorward, John Haughney, Fiona Hogarth, Denise Kendrick, Roberta Littleford, Alex McConnachie, Colin McCowan, Nicola McMeekin, Manish Patel, Petra Rauchhaus, Lewis Ritchie, Chris Robertson, John Robertson, Jose Robles-Zurita, Joseph Sarvesvaran, Herbert Sewell, Michael Sproule, Thomas Taylor, Agnes Tello, Shaun Treweek, Kavita Vedhara, Stuart Schembri

**Affiliations:** 1 School of Medicine, University of St Andrews, St Andrews, UK; 2 Institute of Health and Wellbeing, University of Glasgow, Glasgow, UK; 3 Respiratory Medicine, NHS Tayside, Dundee, UK; 4 Tayside Clinical Trials Unit, University of Dundee, Dundee, UK; 5 Dept of Health Services Research and Policy, London School of Hygiene and Tropical Medicine, London, UK; 6 Radiology, NHS Lanarkshire, Bothwell, UK; 7 Respiratory Medicine, NHS Greater Glasgow and Clyde, Glasgow, UK; 8 General Practice, NHS Greater Glasgow and Clyde, Glasgow, UK; 9 School of Medicine, University of Nottingham, Nottingham, UK; 10 Centre for Clinical Research, University of Queensland, Saint Lucia, Australia; 11 Respiratory Medicine, NHS Lanarkshire, Bothwell, UK; 12 The Institute of Applied Health Sciences, University of Aberdeen, Aberdeen, UK; 13 Dept of Mathematics and Statistics, University of Strathclyde, Glasgow, UK; 14 School of Life Sciences, University of Nottingham, Nottingham, UK; 15 Radiology, NHS Greater Glasgow and Clyde, Glasgow, UK; 16 Radiology, NHS Tayside, Dundee, UK; 17 Health Services Research Unit, University of Aberdeen, Aberdeen, UK

## Abstract

The EarlyCDT-Lung test is a high-specificity blood-based autoantibody biomarker that could contribute to predicting lung cancer risk. We report on the results of a phase IV biomarker evaluation of whether using the EarlyCDT-Lung test and any subsequent computed tomography (CT) scanning to identify those at high risk of lung cancer reduces the incidence of patients with stage III/IV/unspecified lung cancer at diagnosis compared with the standard clinical practice at the time the study began.

The Early Diagnosis of Lung Cancer Scotland (ECLS) trial was a randomised controlled trial of 12 208 participants at risk of developing lung cancer in Scotland in the UK. The intervention arm received the EarlyCDT-Lung test and, if test-positive, low-dose CT scanning 6-monthly for up to 2 years. EarlyCDT-Lung test-negative and control arm participants received standard clinical care. Outcomes were assessed at 2 years post-randomisation using validated data on cancer occurrence, cancer staging, mortality and comorbidities.

At 2 years, 127 lung cancers were detected in the study population (1.0%). In the intervention arm, 33 out of 56 (58.9%) lung cancers were diagnosed at stage III/IV compared with 52 out of 71 (73.2%) in the control arm. The hazard ratio for stage III/IV presentation was 0.64 (95% CI 0.41–0.99). There were nonsignificant differences in lung cancer and all-cause mortality after 2 years.

ECLS compared EarlyCDT-Lung plus CT screening to standard clinical care (symptomatic presentation) and was not designed to assess the incremental contribution of the EarlyCDT-Lung test. The observation of a stage shift towards earlier-stage lung cancer diagnosis merits further investigations to evaluate whether the EarlyCDT-Lung test adds anything to the emerging standard of low-dose CT.

## Abstract 2

**A positive EarlyCDT-Lung test, followed by CT, significantly reduced the numbers of late-stage cancers: performance of the blood test should be assessed further in a screening study where all eligible participants in the intervention arm have a CT scan** https://bit.ly/32maUrB

## Introduction

The 5-year lung cancer mortality rates of 80–90% remain unacceptably high and the UK's survival rate is poor by international comparisons [1]. To improve the poor prognosis, methods are needed that detect lung cancer at an earlier stage, when it is more likely to be treated with curative intent. Several clinical trials have reported that low-dose computed tomography (LDCT) screening can reduce lung cancer mortality by ∼20% [2–5]. Most recently, the NELSON trial reported a 24% reduction in lung cancer mortality from screening after 10 years of follow-up of 13 131 males [4]. However, no difference in all-cause mortality was demonstrated in NELSON nor in other large trials to date with follow-up >5 years, including the US-based National Lung Screening Trial [3–9]. That LDCT screening can reduce lung cancer mortality has provided impetus to consider national screening programmes for the early detection of lung cancer. However, the widespread adoption of LDCT screening will likely remain limited by resource constraints and concerns about overdiagnosis [10]. Cost-effective national screening programmes in the UK are likely to have to take a more targeted approach to LDCT. A biomarker test could potentially play a role in identifying those most at risk and who have most to gain from a targeted approach [11].

The EarlyCDT-Lung test is an ELISA that measures seven autoantibodies, each with individual specificity for the following tumour-associated antigens: p53, NY-ESO-1, CAGE, GBU4-5, HuD, MAGE A4 and SOX2. Autoantibodies can be detected in peripheral blood in patients with solid tumours up to 3–4 years before symptomatic presentation, although it is not yet clear how long autoantibodies continue to be present once triggered [12, 13]. In clinical studies of symptomatic lung cancer and a high-risk cohort study, the EarlyCDT-Lung test has demonstrated a specificity of 91% and sensitivity of 37–41% [14, 15]. The Early Diagnosis of Lung Cancer Scotland (ECLS) trial was a phase IV (prospective screening) biomarker evaluation that addressed the question: “Does using the EarlyCDT-Lung test to identify those at high risk of lung cancer and any subsequent CT scanning reduce the incidence of patients with late-stage lung cancer (III and IV) or unclassified presentation at diagnosis, compared with standard clinical practice?”.

## Methods

ECLS was a pragmatic randomised controlled trial involving 12 208 participants recruited through general practices and community-based recruitment strategies in Scotland in the UK [16]. Recruitment occurred between April 2013 and July 2016, and follow-up was undertaken for 24 months after randomisation for each participant. Adults age 50–75 years at increased risk of developing lung cancer compared with the general population were eligible to participate. These were defined as current or former cigarette or tobacco smokers with at least 20 pack-years, or with a history of smoking of <20 pack-years plus immediate family history (mother, father, sibling or child) of lung cancer. Potential trial participants were identified from the electronic medical records of general practices that were located in the most socioeconomically deprived areas in Scotland or they self-referred in response to a range of advertising methods. Trial participants had no symptoms suggestive of current malignancy, terminal illness or immunosuppressant therapy and had an Eastern Cooperative Oncology Group performance status of 0–2 at recruitment.

The study was conducted in accordance with the principles of Good Clinical Practice and the UK National Research Governance Framework [17]. The University of Dundee and Tayside Health Board co-sponsored the trial, which was registered at ClinicalTrials.gov with identifier NCT01925625. Institutional Review Board approval was provided by the East of Scotland Research Ethics Committee (REC 13/ES/0024). Funding for the trial was obtained from the Scottish Government and the test manufacturer Oncimmune (Nottingham, UK). The trial was conducted in accordance with the protocol [16]; the protocol and the statistical analysis plan are available in the supplementary material. An independent Trial Steering Committee provided trial oversight. The report herein adheres to the CONSORT statement and Aarhus guidelines for the reporting of clinical trials on early cancer diagnosis [18, 19].

### Randomisation and masking

All participants who gave informed consent provided a blood sample prior to randomisation. Participants were then individually randomised, stratified by recruitment site (Tayside, Glasgow and Lanarkshire), and minimised by age, sex and smoking status. Smoking cessation advice was offered in keeping with NHS Scotland advice. Participants allocated to the intervention arm were tested with the EarlyCDT-Lung test. If this was positive, they received a baseline chest radiograph (in order to prioritise access to CT for patients with positive findings on chest radiography) and chest LDCT scan followed by 6-monthly LDCT scans up to 24 months post-randomisation (supplementary table S1). Images from test-positive participants were reviewed by a panel of experienced thoracic radiologists and respiratory physicians. Test-positive participants were followed-up within the study or *via* the NHS care pathway (following the prevailing Fleischner Society guidelines), whichever was most intensive [20]. Participants allocated to the control arm and those who were test-negative received standard clinical care in the NHS in Scotland following national guidelines for identification and management of symptoms suggestive of lung cancer with no further study investigations [21].

Blood samples were processed according to the protocol (supplementary material) and Standard Operating Procedures, consistent with relevant UK and US guidelines. The EarlyCDT-Lung test was performed on 0.5 mL plasma samples. All test-positive, and a random sample of test-negative and control arm participants recruited between December 2013 and April 2015, were invited to complete study questionnaires measuring psychological and smoking outcomes, the EuroQol-5D questionnaire, and health service use (supplementary table S2). Invitation to complete the study questionnaires was done at 1, 3, 6 and 12 months for the test-negative and control arms, with additional questionnaire testing at 18 and 24 months for participants in the test-positive group. These results are reported elsewhere [22, 23].

With participant consent, validated data on cancer occurrence, mortality and comorbidities were obtained from National Services Scotland, which is a high-quality health services data repository. These were linked and analysed in the Dundee Health Informatics Centre Safe Haven.

Pathology and tumour staging reports were prepared by independent assessors who were blinded to the allocation status of participants. Staging data were taken from the Scottish Cancer Registry (SMR06) [24]. The primary outcome variable extracted from SMR06 was the first occurrence of all diagnoses starting with the International Statistical Classification of Diseases and Related Health Problems 10th Revision codes C33 (primary malignant neoplasm of trachea) and C34 (bronchus or lung). Where more than one lung cancer tumour was present at diagnosis, the most advanced tumour was used for classification of disease. To determine staging, reported clinical and pathological “T (tumour), N (node), M (metastasis)” were used with pathological staging taking precedence when present by data analysts blinded to allocation status. Lung tumour histology was coded in accordance with the International Classification of Diseases for Oncology Third Edition and lung cancer staging was determined using the TNM Classification of Malignant Tumours Seventh Edition [25].

### Sample size

During study planning, the background rate of lung cancer was 187 per 100 000 per year for people aged 50–75 years in Scotland. Those in the most deprived quintile were associated with an increased risk of 1.8 times compared with the middle quintile of deprivation [26, 27]. The ECLS study population was selected using similar entry criteria as the Mayo screening study in the USA [26]. The precise baseline rate of stage III/IV presentation for the high-risk population envisaged in this study was uncertain, as was the size of the reduction in stage III/IV presentation likely to be achieved through use of the EarlyCDT-Lung test. Based on the literature and expert opinion, we estimated a stage III/IV presentation rate of 1200 per 100 000 per year in the control group, resulting in an estimated 2.4% prevalence rate over the 2-year follow-up period. Using this estimate and 85% power at 5% significance (two-sided), we wanted to be able to detect a 35% reduction in the rate of stage III/IV presentation in the intervention arm. Based on discussion with a range of stakeholders, this was considered likely to be sufficiently clinically significant to influence practice. Taken together, we estimated the event rate over the 2 years of follow-up at 120 events in the control arm and 78 events in the intervention arm, and required a sample size of n=5000 per arm.

The protocol allowed for the sample size to be modified if the observed event rate proved to be markedly different from the modelled estimates. The sample size was revised to 12 000 in 2015, after recruitment of approximately 8600 participants, when it appeared that, while still meeting trial eligibility criteria, our initial assumption of the rate of stage III/IV presentation had been overestimated. The increase in sample size was achieved by adding an extra recruitment centre (Lanarkshire) and extending the recruitment period. The revised power was 85% at 5% significance (two-sided) to detect a 35% reduction in stage III/IV lung cancer, based on a rate of 600 per 100 000 with a 3-year recruitment period and 2 years of follow-up, with no loss to follow-up anticipated.

### Statistical analysis

The primary analysis compared the rate of stage III/IV lung cancer within 2 years of randomisation between the intervention and control arms. The analyses followed the intention-to-treat principle. Cox proportional hazards models were used to estimate the hazard ratio. One participant who withdrew consent for use of their data was excluded from analysis. The models were adjusted for age, sex, smoking history, socioeconomic status and general practice.

Similar methodology was used to analyse the secondary outcomes of mortality rates. Further analysis compared the outcomes of those in the intervention arm with a positive test, those in the intervention arm with a negative test and those in the control arm. Comparisons of proportions were carried out using Fisher's exact test due to the small number of events. Poisson regression models (adjusting for follow-up time when necessary) were used to investigate other clinical outcomes. Specificity and sensitivity were calculated, but these are estimated values as the true figures are not estimable for early stage and late stage separately. This is because the test-positives received a more intensive intervention than the test-negatives and in a prospective study cancer status is unknown most of the time. The full statistical analysis plan can be found in the supplementary material.

A within-trial model-based cost-effectiveness analysis was conducted, estimating the cost per stage I/II lung cancer case detected comparing the intervention with the control arm. Diagnostic costs were included for all groups. A model-based approach was taken for two reasons: 1) prevalence of lung cancer during the trial was different between arms (our model assumed the same prevalence in both arms) and 2) data about resource use for detection were only available for test-positive participants (n=598) during the trial, therefore resource use was modelled. Full assumptions and parameters used in the model are presented in the supplementary material. Briefly, detection resources comprised the EarlyCDT-Lung test, monitoring tests and confirmatory diagnostic tests. The outcome was number of stage I/II lung cancers detected within the 2-year follow-up. Treatment costs are not included in this within-trial cost-effectiveness analysis.

## Results

### Characteristics of the participants

A total of 77 077 invitation letters were sent to people fulfilling the medical record search criteria from 166 general practices; 16 268 people responded. An additional 2389 potential participants self-referred in response to advertising. 12 241 were invited to an in-person screening appointment and 12 215 were randomised. The recruitment rate of people identified as potential study participants from general practice records was 13.4% (10 352 out of 77 077). Six participants were excluded post-randomisation but prior to receiving imaging because of ineligibility. One participant who withdrew consent for use of their data was excluded from analysis, leaving 12 208 participants. Participant characteristics were balanced between arms (table 1). 51.8% of participants lived in the two most deprived quintiles, the mean±sd age at recruitment was 60.5±6.58 years and the mean±sd pack-years smoked was 38.2±18.58. The incidence rate of lung cancer in the trial population, as determined from cancer registry data, was 520 per 100 000 per annum (0.52%).

**TABLE 1:** Selected baseline characteristics of the trial participants

	**Intervention (EarlyCDT-Lung test)**	**Control (standard clinical care)**
**Subjects**	6088	6121
**Age at randomisation years**		
50–54	1393 (22.9)	1409 (23.0)
55–59	1562 (25.7)	1531 (25.0)
60–64	1300 (21.4)	1318 (21.5)
65–69	1179 (19.4)	1203 (19.7)
70–75	654 (10.7)	660 (10.8)
**Sex**		
Male	3095 (50.8)	3129 (51.1)
Female	2993 (49.2)	2992 (48.9)
**SIMD quintile**		
1 (most deprived)	1751 (28.8)	1726 (28.2)
2	1431 (23.5)	1420 (23.2)
3	1108 (18.2)	1121 (18.3)
4	966 (15.9)	1002 (16.4)
5	782 (12.8)	792 (12.9)
No information	50 (0.8)	60 (1.0)
**Smoking status**		
Current smoker	3199 (52.5)	3178 (51.9)
Ex-smoker	2889 (47.5)	2943 (48.1)
Quit ≥1 week	2207 (36.3)	2283 (37.3)
Quit ≥6 months	1998 (32.8)	2083 (34.0)
Pack-years	38.4±18.7	38.0±18.5
Family history	1550 (25.5)	1614 (26.4)
**Comorbidity**		
COPD	306 (5.0)	287 (4.7)

Data are presented as n or n (%). SIMD: Scottish Index of Multiple Deprivation; COPD: chronic obstructive pulmonary disease.

### Adherence to protocol

**FIGURE 1:**
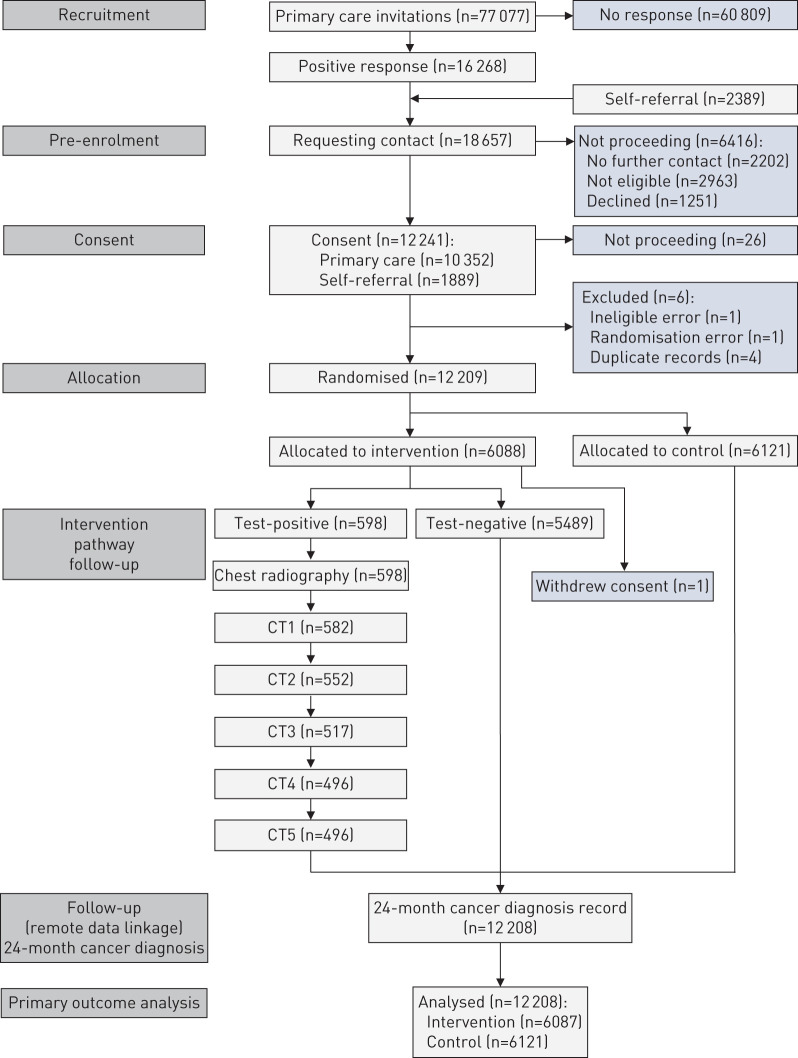
CONSORT flowchart. CT: computed tomography.

We accessed the records of 99.9% of the study population; the CONSORT flowchart (figure 1) presents the end-point ascertainment in the intervention and control arms. The CONSORT statement is available in the supplementary material.

Follow-up was performed using a national, closed administrative data system for 24 months after individual randomisation or to death if within the follow-up period. We also checked national prescribing and inpatient/outpatient data systems for activity relating to trial participants in the 2-year post-randomisation follow-up period. We confirmed health service contacts in the 2-year follow-up for 10 973 (89.9%) of the participants: 5489 in the intervention arm and 5484 in the control arm. When the 1235 patients who did not record health service contacts during this period were removed from the analysis, the key findings were unchanged (data pack S3 in the supplementary material).

### Results of testing

**FIGURE 2:**
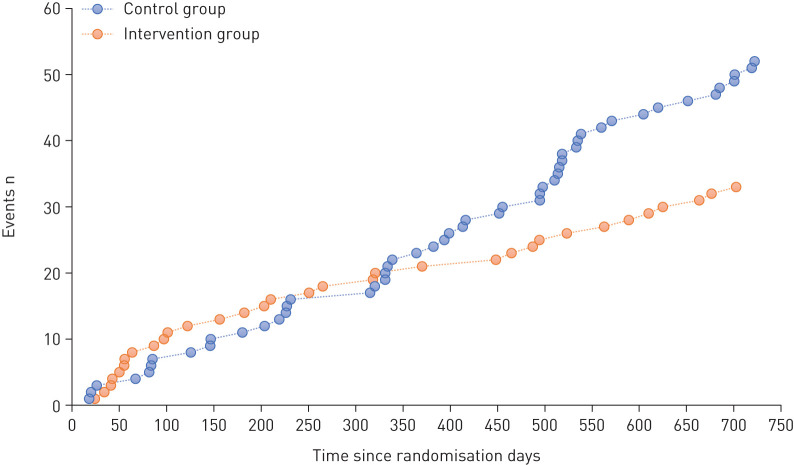
Primary outcome: diagnosis of stage III/IV/unspecified lung cancer 2 years after randomisation in the intervention and control arms.

The results of the primary analysis are presented in table 2 and figure 2. 9.8% (598 out of 6087) of participants in the intervention arm had a positive EarlyCDT-Lung test and 3.0% (n=18) of these had a confirmed case of lung cancer within 2 years. In the test-negative arm, 0.7% (n=38) had confirmed lung cancers. For the intervention group as a whole, 0.92% (n=56) had confirmed lung cancer within 2 years. In the control arm, 1.16% (n=71) had confirmed lung cancer within 2 years. The percentage of stage III/IV/unspecified lung cancer diagnosis in the intervention and control arm was 0.5% (33 out of 6087) and 0.8% (52 out of 6121), respectively. The absolute risk reduction in stage III/IV/unspecified lung cancer diagnosis was 0.3% (95% CI 0.01–0.6). The number of participants to be screened to prevent one stage III/IV/unspecified lung cancer diagnosis was 325 (95% CI 13–637) and the hazard ratio for stage III/IV presentation was 0.64 (95% CI 0.41–0.99; p=0.0432) (data pack S2 in the supplementary material).

**TABLE 2:** Stage of lung cancer at diagnosis in the intervention and control arms

	**Intervention**		**Control (standard clinical care)**
	**Test-positive**	**Test-negative**	
**Subjects**	598	5489	6121
**Stage**			
I	10 (1.7)	7 (0.1)	9 (0.1)
II	2 (0.3)	4 (0.1)	10 (0.2)
III	3 (0.5)	12 (0.2)	17 (0.3)
IV	3 (0.5)	15 (0.3)	28 (0.5)
Unspecified	0 (0.0)	0 (0.0)	7 (0.1)
No lung cancer	580 (97.0)	5451 (99.3)	6050 (98.8)

Data are presented as n or n (%).

Although we did not perform LDCT on all study subjects, the estimated test performance characteristics using cancer registry data after 2 years of follow-up as the reference standard are described in table 3. The EarlyCDT-Lung test had an estimated sensitivity of 52.2% (95% CI 30.6–73.2%) for stage I/II disease and 18.2% (95% CI 7.0–35.5%) for stage III/IV disease, and specificity of 90.3% (95% CI 89.6–91.1%) for stage I/II disease and 90.2% (95% CI 89.4–91.0%) for stage III/IV disease. The positive predictive value was 2.0% (95% CI 1.0–3.5%) for stage I/II disease and 1.0% (95% CI 0.4–2.2%) for stage III/IV disease, and the negative predictive value was 99.8% (95% CI 99.6–99.9%) for stage I/II disease and 99.5% (95% CI 99.3–99.7%) for stage III/IV disease in the population studied.

**TABLE 3:** Estimated EarlyCDT-Lung test performance characteristics 6 months, 1 year and 2 years after randomisation

	**Test-positive**	**Test-negative**	**Sensitivity**	**Specificity**	**Positive predictive value**	**Negative predictive value**
**Subjects**	598	5489				
**Stage of lung cancer 6 months after randomisation (*post hoc*)**						
I/II	7 (1.2)	2 (0.0)	77.8 (40.0–97.2)	90.3 (89.5–91.0)	1.2 (0.5–2.4)	100.0 (99.9–100.0)
III/IV	5 (0.8)	8 (0.2)	38.5 (13.9–68.4)	90.2 (89.5–91.0)	0.8 (0.3–1.9)	99.9 (99.7–99.9)
I–IV	12 (2.0)	10 (0.2)	54.6 (32.2–75.6)	90.3 (89.6–91.1)	2.0 (1.0–3.5)	99.8 (99.7–99.9)
**Stage of lung cancer 1 year after randomisation (*post hoc*)**						
I/II	9 (1.5)	4 (0.1)	69.2 (38.6–90.9)	90.3 (89.5–91.0)	1.5 (0.7–2.8)	99.9 (99.8–100.0)
III/IV	6 (1.0)	14 (0.2)	30.0 (11.9–54.3)	90.2 (89.5–91.0)	1.0 (0.4–2.2)	99.7 (99.6–99.9)
I–IV	15 (2.5)	18 (0.3)	45.5 (28.1–63.6)	90.4 (89.6–91.1)	2.5 (1.4–4.1)	99.7 (99.5–99.8)
**Stage of lung cancer 2 years after randomisation**						
I/II	12 (2.0)	11 (0.2)	52.2 (30.6–73.2)	90.3 (89.6–91.1)	2.0 (1.0–3.5)	99.8 (99.6–99.9)
III/IV	6 (1.0)	27 (0.5)	18.2 (7.0–35.5)	90.2 (89.4–91.0)	1.0 (0.4–2.2)	99.5 (99.3–99.7)
I–IV	18 (3.0)	38 (0.7)	32.1 (20.3–46.0)	90.4 (89.6–91.1)	3.0 (1.8–4.7)	99.3 (99.1–99.5)

Data are presented as n, n (%) or % (95% CI). Absolute risk reduction of late-stage lung cancer diagnosis 2 years after randomisation was 0.31%. The number needed to screen to prevent one late-stage lung cancer diagnosis 2 years after randomisation was 325 (95% CI 13–637).

**FIGURE 3:**
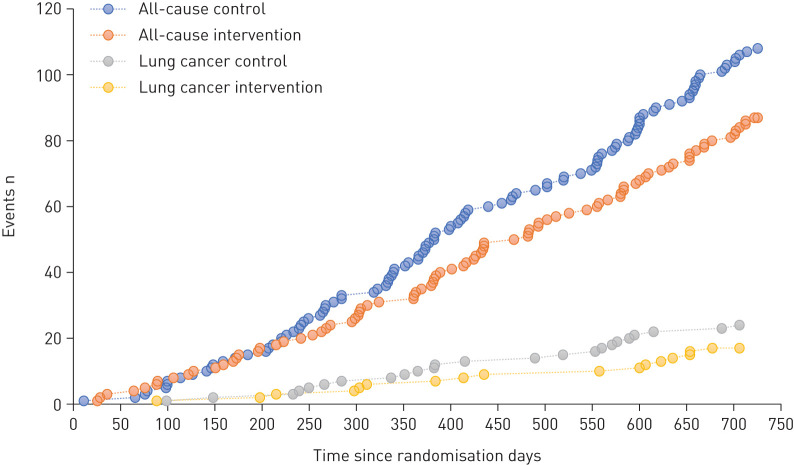
Secondary outcomes: mortality 2 years after randomisation in the intervention and control arms.

Figure 3 shows the secondary outcomes of lung cancer and all-cause mortality at 2 years, and demonstrates divergence after the first year of follow-up. In the intervention arm, there were fewer events than in the control arm for all-cause mortality. There were nonsignificant differences in lung cancer mortality (intervention arm 17 out of 6087 (0.28%) *versus* control arm 24 out of 6121 (0.39%)) and all-cause mortality (intervention arm 87 out of 6087 (1.43%) *versus* control arm 108 out of 6121 (1.76%)) after 2 years. Participants in the intervention arm were diagnosed with lung cancer on average 87.3 days earlier (mean 303.0 (95% CI 214.9–364.0) days) compared with the control arm (mean 390.3 (95% CI 340.6–440.1) days) (data pack S3 in the supplementary material).

The cost per early case (stage I/II) detected after 2 years was GBP116 000 (95% CI GBP54 900 to dominated, *i.e.* screening using the EarlyCDT-Lung test would be more costly and less effective than the control arm) (supplementary table S3). Sensitivity analyses were conducted varying prevalence and test costs; results indicate that cost-effectiveness was most affected by changes in prevalence. More detailed analyses are planned to extrapolate the cost per case detected to a full lifetime cost per quality-adjusted life year (QALY) gained analysis, including stage-specific treatment costs (supplementary material). As not all required data are available at the time of writing, comprehensive cost-effectiveness analyses will be presented in a subsequent article.

### Adverse events

Five adverse events, as defined in the protocol as being directly related to the intervention (collection of blood sample), were reported and all were considered minor. For those in the intervention arm, there was one injection site haematoma, one panic attack and one pre-syncope. In the control arm there were two episodes of syncope.

## Discussion

This is the first trial conducted as a phase IV evaluation of a blood-based biomarker panel for lung cancer. The results show a significant decrease in the incidence of advanced stage disease, thereby meeting the primary end-point of the study. In the study population as a whole, the absolute risk reduction in stage III/IV lung cancer diagnosis for those in the intervention arm was 0.3%. For those participants given a lung cancer diagnosis in the study period, there was a 14.3% absolute risk reduction (33 out of 56 *versus* 52 out of 71) in stage III/IV lung cancer presentation in the intervention arm. After the short follow-up period of 2 years, there were nonsignificant reductions in lung cancer mortality: 29.2% in relative risk (control 24 *versus* intervention 17) and 19.4% in all-cause mortality (control 108 *versus* intervention 87).

Community-based trials such as ECLS are more likely to produce generalisable results than those conducted in academic health centres, which often recruit from a more tightly selected population [28]. Strengths of our trial include community recruitment with a high proportion of participants recruited by their general practitioners from the two most socioeconomically deprived quintiles (51.8%) of the Scottish population, integration with a national healthcare system providing whole-population care, a high end-point ascertainment rate (>99.9%) and the intention-to-treat analysis.

The lung cancer diagnosis rate (1%) was lower than we anticipated when planning the study and lower than might be expected from other studies using LDCT. Therefore, our approach, in contrast to LDCT, may have missed early-stage lung cancers. Our follow-up period of 2 years was short and cases will continue to emerge as the study final results become available. Another potential contributor to the lower rate of diagnosis may be the “healthy volunteer” effect, which may have led to a higher rate of recruitment of the healthiest among the at-risk population meeting our inclusion criteria [29]. It is worth noting that even with a lower rate of lung cancer, those in the intervention arm were at a statistically significant and clinically important reduced risk of stage III/IV presentation. The results of this study are not directly comparable to those using a validated questionnaire before LDCT [30]. A direct comparison of both methods would need to be undertaken to determine how a biomarker test compares to a questionnaire followed by LDCT. A control arm involving CT screening would have provided evidence comparing the US Preventive Task Force guidelines against a “biomarker first” approach, but CT screening was not available when the ECLS trial started and remains unavailable in many health systems, including the UK.

The finding that there were more lung cancers in the control arm (71 *versus* 56 in the intervention arm) was also unexpected as CT screening trials usually report more cancers diagnosed in the intervention arm. We consider that there are four potential reasons for this. First, as discussed earlier, we may have not diagnosed all cases of lung cancer. Second, although treatment arms were well balanced due to randomisation, there may be differences between arms in unmeasured risk factors, such as asbestos exposure [31]. Third, false reassurance in the test-negative arm (leading to risk-reduction behaviours in those receiving the EarlyCDT test) may also be a potential explanation. A recent systematic review found that negative test results are unlikely to cause false reassurance, anxiety or a change in health-related behaviours; hence, we consider it unlikely that false reassurance had a substantial impact on lung cancer presentation in those with negative test results [32]. Finally, the observed numerical difference is not statistically significant and could be due to chance (p=0.2)

We have presented a short-term within-trial analysis of cost-effectiveness data. A recent study has suggested that the use of an autoantibody test in patients with pulmonary nodules is a cost-effective use of healthcare resources [33]. The base case cost per QALY gained of GBP116 000 is a within-trial estimate, and does not include long-term costs of treatment and survival beyond the trial. Longer-term analyses will employ a model to link the short-term outcomes measured within the trial to longer-term health impacts (*e.g.* morbidity and mortality), and will consider the longer-term impact of early detection and treatment on the cost per QALY gained in the context of more effective and expensive therapies.

The seven autoantibodies to the tumour-associated antigen panel of the EarlyCDT-Lung test demonstrated high specificity (90.4%) and moderate sensitivity (32.1%) for detecting lung cancer at 2 years. The moderate sensitivity of the test at 2 years may be due to tumour-induced suppression of immune responses that lead to less autoantibody production and detection [34]. The study measured the EarlyCDT-Lung test only once at baseline and so does not address the frequency at which the test might be repeated. A previous report showed that in patients with lung nodules the EarlyCDT-Lung test enhanced the positive predictive power of nodule-based risk assessment for the detection of lung cancer [35]. The high specificity of the EarlyCDT-Lung test could be used in combination with LDCT, which demonstrates high sensitivity, to ensure a high detection rate of stage I/II lung cancer cases. Recent developments in the use of artificial intelligence in imaging and other biomarkers are also likely to be important [36].

In conclusion, ECLS demonstrates that blood-based biomarker panels, such as the EarlyCDT-Lung test, followed by LDCT can detect stage I/II lung cancer. Follow-up analyses will be performed after 5 and 10 years, although we recognise that the absolute lung cancer incidence would be higher than that detected due to deaths from other causes. Further investigation in large, community-based studies will be required to determine the long-term impact of performing the EarlyCDT-Lung test with LDCT on mortality, cost-effectiveness, the level of risk that should be targeted, the time interval between tests and how to improve the engagement of people at the highest risk [37].

## Supplementary material

10.1183/13993003.00670-2020.Supp1 **Please note:** supplementary material is not edited by the Editorial Office, and is uploaded as it has been supplied by the author.
Appendices ERJ-00670-2020_Appendices
Supplementary_Tables ERJ-00670-2020_Supplementary_Tables

## Shareable PDF

10.1183/13993003.00670-2020.Shareable1 This one-page PDF can be shared freely online.
Shareable PDF ERJ-00670-2020.Shareable
