# Supplementary material for: Earlier diagnosis of lung cancer in a randomised trial of an autoantibody blood test followed by imaging
Source: Eur Respir J. 2021 Jan 14;57(1):2000670. doi: 10.1183/13993003.00670-2020 (PMC7806972; doi:10.1183/13993003.00670-2020)
Supplement: Supplementary file 1 [file ERJ-00670-2020_Appendices.pdf]

APPENDICES (SA)

Manuscript Title: Improved diagnosis of earlier-stage lung cancer in a randomised trial of an autoantibody blood test followed by imaging. The Early Diagnosis of Lung Cancer Scotland (ECLS) Team

Table of Contents:

The Early Diagnosis of Lung Cancer Scotland (ECLS) Team	SA. 2
Appendix 1. ECLS Study Protocol; Version 8.1 11-10-2018	SA. 4
Appendix 2. Statistical Analysis Plan; Version 1.0	SA. 145
Appendix 3. Within-trial model-based cost effectiveness analysis: assumptions and parameters.	SA. 168
Appendix 4. CONSORT 2010 Checklist for Reporting a Randomised Trial	SA. 171
Appendix 5. Health Economics Analysis Plan; Version 2.0	Separate document

The Early Diagnosis of Lung Cancer Scotland (ECLS) Team

Trial Steering Committee

**Name**	**Role**	**Affiliate Institution**
Prof David Weller	Primary Care	Edinburgh University
Dr Bernard Higgins	Respiratory physician	NHS Newcastle Upon Tyne
Prof Stephen Spiro	Respiratory physician	University College Hospital
Prof Amanda Lee	Statistics	Aberdeen University

Trial Management Committee

Name	Role	Affiliate Institution
Prof Frank Sullivan	Chief Investigator (Primary care)	St Andrews University
Dr Stuart Schembri	Co-Chief Investigator (Radiology)	Dundee University
Ms Joana Rocha	Database management	Tayside Clinical Trials Unit
Mr Michael Hannah	Database management	Tayside Clinical Trials Unit
Prof Andrew Briggs	Health economics	London School of Hygiene & Tropical Medicine
Dr Jose Robles-Zurita	Health Economics	Glasgow University
Ms Nicola Mcmeekin	Health economics	Glasgow University
Herb Sewell	Immunology	Nottingham University
Prof Cathy Jackson	Primary care	St Andrews University
Prof Denise Kendrick	Primary care	Nottingham University
Prof Frances Mair	Primary care	Glasgow University
Dr John Haughney	Primary care	NHS GGC
Dr Agnes Tello	Project management	St Andrews University
Prof Chris Robertson	Public health	Strathclyde University
Prof Lewis Ritchie	Public health	Aberdeen University
Prof Kavita Vedhara	Qualitative research	Nottingham University
Roderick Cameron	Radiology	NHS Tayside
Dr Cindy Chew	Radiology	NHS Lanarkshire
Dr Michael Sproule	Radiology	NHS GGC
Dr Thomas Taylor	Radiology	NHS Tayside
Dr William Anderson	Respiratory medicine	NHS Tayside
Dr Alistair Dorward	Respiratory medicine	NHS GGC
Dr Joseph Sarvesvaran	Respiratory medicine	NHS GGC
Dr Manish Patel	Respiratory medicine	NHS Lanarkshire
Dr Robert Milroy	Respiratory medicine	NHS GGC
Prof Alex McConnachie	Statistics	Glasgow University
Ms Petra Rauchhaus	Statistics	Tayside Clinical Trials Unit
Prof John Robertson	Surgery	Nottingham University
Ms Emma Mackenzie	Trial information systems management	Tayside Clinical Trials Unit

Mrs Pauline Armory	Trial management	Tayside Clinical Trials Unit
Dr Roberta Littleford	Trial management	Tayside Clinical Trials Unit
Prof Colin McCowan	Trial methodology	St Andrews University
Prof Shaun Treweek	Trial methodology	Aberdeen University
Dr Fiona Hogarth	Trials Unit Co-Director	Tayside Clinical Trials Unit

ECLS Investigators for Current Publication

Name	Role	Affiliation
Prof Frank Sullivan	Chief Investigator (Primary care)	St Andrews University
Dr Stuart Schembri	Co-Chief Investigator (Radiology)	Dundee University
Prof Andrew Briggs	Health economics	London School of Hygiene & Tropical Medicine
Dr Jose Robles-Zurita	Health Economics	Glasgow University
Ms Nicola Mcmeekin	Health economics	Glasgow University
Prof Herb Sewell	Immunology	Nottingham University
Prof Denise Kendrick	Primary care	Nottingham University
Prof Frances Mair	Primary care	Glasgow University
Dr John Haughney	Primary care	NHS GGC
Dr Agnes Tello	Project management	St Andrews University
Prof Chris Robertson	Public health	Strathclyde University
Prof Lewis Ritchie	Public health	Aberdeen University
Prof Kavita Vedhara	Qualitative research	Nottingham University
Dr Cindy Chew	Radiology	NHS Lanarkshire
Dr Michael Sproule	Radiology	NHS GGC
Dr Thomas Taylor	Radiology	NHS Tayside
Dr William Anderson	Respiratory medicine	NHS Tayside
Dr Alistair Dorward	Respiratory medicine	NHS GGC
Dr Joseph Sarvesvaran	Respiratory medicine	NHS GGC
Dr Manish Patel	Respiratory medicine	NHS Lanarkshire
Prof Alex McConnachie	Statistics	Glasgow University
Ms Petra Rauchhaus	Statistics	Tayside Clinical Trials Unit
Prof John Robertson	Surgery	Nottingham University
Mrs Pauline Armory	Trial management	Tayside Clinical Trials Unit
Dr Roberta Littleford	Trial management	Tayside Clinical Trials Unit
Prof Colin McCowan	Trial methodology	St Andrews University
Prof Shaun Treweek	Trial methodology	Aberdeen University
Dr Fiona Hogarth	Trials Unit Co-Director	Tayside Clinical Trials Unit

Appendix 1. ECLS Study Protocol; Version 8.1 11-10-2018

Detection in blood of autoantibodies to tumour antigens as a case-finding method in lung cancer using the EarlyCDT-Lung test
Version 8.1 11-10-2018

ECLS STUDY	**ECLS Study Protocol** Detection in blood of autoantibodies to tumour antigens as a case-finding method in lung cancer using the EarlyCDT-Lung test

**Study Acronym**	**ECLS**
**Sponsor**	University of Dundee - NHS Tayside
**Sponsor R&D Number**	2013ON07
**Coordinating Trial Centre**	Tayside CTU
**Funder**	Chief Scientist Office, Scottish Government<br>Oncimmune Ltd
**Chief Investigator**	Professor Frank Sullivan
**REC Number**	***13/ES/0024***
**ClinicalTrials.Gov ID**	NCT01925625
**Version Numbers and Dates**	Version 8.1, 11-10-18

CONTACT NAMES AND ADDRESSES

Prof F M Sullivan (Chief Investigator), Head of Division Population & Behavioural Science, Medical School Director of Research, University of St. Andrews, St. Andrews, Fife. KY16 9TF
fms20@st-andrews.ac.uk 01334 463512

Dr Stuart Schembri (Co-Chief Investigator/Principal Investigator), Senior Staff Specialist Department of Respiratory and Sleep, The Canberra Hospital, Garran, ACT 2605, Australia
s.schembri@dundee.ac.uk

Dr Fiona Hogarth, Tayside Clinical Trials Unit) Tayside Clinical Trials Unit, Ninewells Hospital and Medical School, Dundee DD1 9SY
f.j.hogarth@dundee.ac.uk 01382 383581

University of Dundee · The University of Nottingham · University of Glasgow · University of Aberdeen · NHS Scotland · tctu Tayside Clinical Trials Unit

Primary and secondary objectives and outcomes: summary

	Objectives	Outcomes
**Primary**	to assess the effectiveness of EarlyCDT-Lung test in reducing the incidence of patients with late-stage lung cancer at diagnosis, compared with standard clinical practice;	difference at 24 months after randomisation, between the number of patients with stage 3, 4 or unclassified lung cancer at diagnosis in the intervention arm, and those in the control arm;
**Secondary 1**	to assess the effectiveness of EarlyCDT-Lung test in improving the diagnosis of early-stage lung cancers;	numbers, at 24months after randomisation, in the different stages at diagnosis (3/ 4/ U/ other) in the intervention arm and the control arm;
**2**	to undertake a cost-effectiveness analysis of EarlyCDT-Lung test as a primary screening method in comparison to standard clinical practice;	difference, after 2 years, in the costs and outcomes between the intervention arm and the control arm; cost-effectiveness of the intervention compared to standard clinical practice
**3a**	to compare lung-cancer mortality, all-cause mortality and cancer-specific mortality in high-risk groups provided with EarlyCDT-Lung test, compared with standard practice;	estimates, after 2 years, of lung cancer mortality, all-cause mortality and cancer-specific mortality in the intervention arm and in the control arm; assessment of significance of differences;
**3b**	to compare long-term future mortality in high-risk groups provided with EarlyCDT-Lung test, compared with standard practice;	estimates, after 5 and 10 years of long-term future mortality in the intervention arm and in the control arm; assessment of significance of differences;
**4**	to obtain refined estimates of the sensitivity, specificity, positive predictive value and negative predictive value of EarlyCDT-Lung test;	estimates, after 2 years of **(i)** the number of patients with stage 3, 4 or unclassified lung cancer at diagnosis in the EarlyCDT-Lung test-positive group and those in the EarlyCDT-Lung test-negative group and **(ii)** stage at diagnosis in the EarlyCDT-Lung test-positive and EarlyCDT-Lung test-negative group;

**5**	to assess behavioural outcomes including smoking, psychological outcomes including cancer worry, anxiety, depression, distress specific to clinical investigations;	scores at baseline, and follow-up on EQ5D, Hospital Anxiety and Depression Scale (HADS), Positive and Negative Affect Schedule (PANAS), Revised Illness Perception Questionnaire – Lung Cancer (IPQ-LC), Lung cancer risk perception, Health anxiety subscale of Health Orientation Scale (HOS) and the Lung Cancer Worry Scale (LCWS), Medication, smoking behaviour, demographic details. Follow-up questionnaires include same items, plus Impact of Events Scale (intervention group only), healthcare utilisation and dates and results of follow-up investigations for lung cancer (test positive group only). The HADS is not included in follow- up questionnaires. Follow-up questionnaires are administered between 1 and 24 months to subsets of the control arm and intervention arm; (all participants in the EarlyCDT-positive group will be approached with the recruitment aim of 300 from this group collected at 1,3,6,12,18 and 24 months. The EarlyCDT-negative and control groups will be recruited at the same rate as the EarlyCDT-positive group with the recruitment aim of 300 from each group collected at 1,3,6 and 12 months).
**6**	to assess the effectiveness of EarlyCDT-Lung test on other clinical outcomes such as CVD, COPD, other cancers, hospital stays and outcomes identified though SMR linkage, etc.;	Incidence of other clinical outcomes such as CVD, COPD, other cancers, hospital stays, identified through SMR linkage, measured at 24 months, 5 and 10 years in the intervention arm and in the control arm; assessment of significance of differences;
**7**	to assess uptake of subsequent investigations such as CXR, CT, bronchoscopy, etc.	numbers in Tayside group (EarlyCDT-Lung test-positive, EarlyCDT-Lung test-negative, control) undertaking subsequent investigations such as CXR, CT, bronchoscopy, etc.

TABLE OF CONTENTS

Contents	7
Protocol Approval	8
List of Abbreviations	9
Summary	10
1. Introduction	16
2. Study Design	23
3. Study Population	27
4. Participant Selection and Enrolment	29
5. Study and Safety Assessments	38
6. Data Collection and Management	40
7. Statistics and Data Analysis	43
8. Trial Management and Oversight Arrangements	48
9. Good Clinical Practice	51
10. Study Conduct Responsibilities	53
11. Reporting, Publication and Notification of Results	55
12. References	56
Appendix 1: Preparatory Focus Group Work	60
Appendix 2: ECLS Study: Qualitative Sub-studies	67
Appendix 3: ECLS Study: START Recruitment Sub-study	90
Appendix 4: Sub-study 5: Exploring Public Perceptions of Lung Cancer Screening (MRC PhD Studentship, University of Glasgow)	109
Appendix 4A: ECLS Non-Attenders Interview Schedule- Understanding why people who are initially interested in lung cancer screening fail to participate	123
Appendix 5: Sub-study 6: Is there a difference in the emotional, cognitive and behavioural response to a positive earlyCDT test if pulmonary nodules are present on a chest computed tomography compared to a normal chest computed tomography?	125
Appendix 6: Living with lung nodules: what information would patients find helpful?	131

Appendix 7: List of Investigators and Collaborators	143

PROTOCOL APPROVAL

Title: Detection in blood of autoantibodies to tumour antigens as a case-finding method in lung cancer using the EarlyCDT-Lung test

ClinicalTrials.Gov ID: NCT01925625

Signatures

By signing this document I am confirming that I have read, understood and approve the protocol for the above study.

**Co- Chief Investigators**	**Signature**	**Date**
Prof F M Sullivan<br>University of St Andrews		
Dr Stuart Schembri<br>The Canberra Hospital		
**Co-Investigators**	**Signature**	**Date**
Professor Denise Kendrick<br>University of Nottingham		28/11/18
Manish Patel<br>NHS Lanarkshire		27/11/18
Dr William Anderson<br>NHS Tayside		28/11/2018
Dr Jennie Hancox<br>University of Nottingham		04/12/2018

**Statistical Review**	**Signature**	**Date**
Dr Petra Rauchhaus<br>Tayside CTU		
Dr Alex McConachie<br>Robertson Centre for Biostatistics,<br>University of Glasgow		

LIST OF ABBREVIATIONS

AAB	Autoantibody
AE	Adverse event
CRF	Care Report Form
CI	Chief Investigator
CNORIS	Clinical Negligence And Other Risks Scheme
CT scan	Computerised Tomography Scan
CXR	Chest X-Ray
eCRF	Electronic case report form
EarlyCDT – Lung Test	Early Cancer Detection Test- Lung Test
ECLS Study	Early Cancer Detection Test – Lung Cancer Scotland Study
GG&C	Greater Glasgow & Clyde
GCP	Good Clinical Practice
HIC	Health Informatics Centre
ICF	Informed Consent Form
ISF	Investigator Site File
SAE	Serious adverse event
SCR	Scottish Cancer Register
SMR	Scottish Morbidity Record
SOP	Standard Operating Procedure
TASC	Tayside Medical Science Centre
TCTU	Tayside Clinical Trials Unit
TAA	Tumour Derived/Associated Antigens
TMF	Trial Master File

SUMMARY

QUESTION / RATIONALE

Lung cancer is the most common cause of cancer-related death worldwide. The majority of cases are detected at a late stage when prognosis is poor. Lung cancer remains the fourth least likely cancer to be picked up early by GPs. Low dose computed tomography (CT) scanning of high risk individuals can reduce lung cancer mortality by 20% but it is expensive and, despite scanning, late stage diagnosis results in substantial morbidity.

The EarlyCDT-Lung Test is an early detection test designed to assist in lung cancer risk assessment and detection in the earliest stages of the disease. Survival rates are much higher when cancer is diagnosed early but because lung cancer is often diagnosed symptomatically, most cases are discovered after the disease has spread. In these cases, the 5-year survival rate is less than 10%. By testing patients who are at a high risk for developing lung cancer before symptoms appear, the EarlyCDT-Lung test could help diagnose lung cancer sooner, when treatment options are more likely to be successful. The EarlyCDT-Lung test detects autoantibodies, which are a patient’s immune response to antigens produced by solid-tumor cells. Because these autoantibodies are produced by healthy individuals at lower levels, the EarlyCDT-Lung test enables physicians to identify those patients producing autoantibodies at higher levels and who are at an increased lung cancer risk or who are already in the early stages of lung cancer.

The EarlyCDT-Lung test can potentially identify those at high risk of lung cancer in whom the benefit/risk ratio for CT scanning is likely to be more favourable. The primary research question is therefore:
Does using the EarlyCDT-Lung test to identify those at high risk of lung cancer and any subsequent CT scanning reduce the incidence of patients with late-stage lung cancer (3 & 4) or unclassified presentation (U) at diagnosis, compared with standard practice?

Secondary questions include, but are not limited to:

i) Is the use of the EarlyCDT-Lung test cost-effective compared to standard clinical practice?

ii) What is the short and long term emotional and behavioural impact of the EarlyCDT-lung test?

iii) Does the EarlyCDT-Lung test improve clinical outcomes including but not limited to cardiovascular disease (CVD), COPD, hospital stays and outcomes identified through SMR linkage?

HYPOTHESIS

In a high risk population the EarlyCDT-Lung test reduces the incidence of late stage tumours; 3 / 4 / Unclassified (U) at diagnosis compared to normal clinical practice.

AIMS

to assess the effectiveness of EarlyCDT-Lung test in increasing early stage lung cancer detection, thereby reducing the rate of late stage (3 / 4 / U) presentation, compared to normal clinical practice;

to assess the cost-effectiveness of EarlyCDT-Lung test compared to normal clinical practice;

to assess the effectiveness of EarlyCDT-Lung test in reducing adverse outcomes including potential psychological and behavioural consequences.

DESIGN

We propose a randomised controlled trial of 12,000 participants. Cancer screening programmes should be based on the high quality evidence which trials provide that they reduce cancer specific mortality. People should be invited to participate in population screening programmes on the basis of firm evidence that the overall balance between potential benefits and harms is favourable. Where screening programmes have relied upon observational data, for example in breast and prostate cancer screening programmes have remained controversial for many years. Eventually in the case of breast cancer large trials have been undertaken to determine the place of the screening method in national programmes. In contrast where large trials have preceded regional and national roll-out of cancer screening programmes e.g. in bowel cancer, the programmes have been more evidence based (for example, population based trials of faecal

occult blood testing have consistently demonstrated significant reductions in colorectal cancer mortality and are summarised in a meta-analysis that indicates a reduction of 16% overall and 25% when adjusted for screening uptake). In the case of lung cancer we have observational data to suggest that the Early CDT-Lung test may be effective and it is now necessary to undertake a trial to determine whether this potential benefit outweighs potential harms and whether the test would be a cost effective use of NHS resources.

SETTING

To recruit participants via general practices, predominately within the lowest quintile of deprivation measured using the Scottish Index of Multiple Deprivation in NHS Tayside, NHS Greater Glasgow & Clyde (GG&C) and NHS Lanarkshire (recruitment in NHS Tayside is now complete.) However, it is anticipated that a number of potential participants will contact the study team in response to the initial media interest surrounding the launch of the study and via family and friends of randomised participants. All interested individuals outwith the GP recruitment strategy will be assessed in relation to inclusion/exclusion criteria including residing within the selected geographical post codes. These participants will be screened at either their participating GP practice or at the local Clinical Research Facility/Centre.

PARTICIPANTS

Adults aged 50 to 75 who are at risk of lung cancer will be eligible to participate. These are defined as those who are current or former cigarette smokers with at least 20 pack-years, or have a history of cigarette smoking less than 20 pack-years plus a family history (mother, father, brother, sister) of lung cancer which gives an individual a personal risk similar to a smoking history of 20 pack years. Participants should be healthy enough to undergo pulmonary resection or stereotactic radiotherapy.

INTERVENTION

EarlyCDT-Lung test followed by imaging studies in those with a positive result.

COMPARATOR

Standard practice of awaiting clinical presentation of symptoms suggestive of lung cancer then investigation by the standard NHS pathway involving chest X-ray, CT scan and bronchoscopy as clinically necessary.

OUTCOMES

Primary

The difference, at 24 months after randomisation, between the rates of patients with stage 3, 4 or unclassified lung cancer at diagnosis in the intervention arm, and those in the control arm;

Secondary

(1) numbers at 24 months after randomisation, in the different stages at diagnosis (3/ 4/ U/ other) in the intervention arm and the control arm;

(2) difference, after 2 years, between costs and outcomes in the intervention arm and in the control arm, cost-effectiveness of EarlyCDT-Lung test compared to normal clinical practice;

(3a) estimates, after 2 years, of lung cancer mortality, all-cause mortality and cancer-specific mortality rates in the intervention arm and in the control arm ; assessment of significance of differences;

(3b) estimates, after 5 years and 10 years, of long-term future mortality rates in the intervention arm and in the control arm; assessment of significance of differences;

(4) estimates, after 2 years of, provided by (i) the number of patients with stage 3, 4 or unclassified lung cancer at diagnosis in the EarlyCDT-Lung test-positive group and those in the EarlyCDT-Lung test-negative group and (ii) stage at diagnosis in the EarlyCDT-Lung test-positive and EarlyCDT-Lung test-negative group;

(5) scores at baseline, and follow-up on in a survey administered prior to treatment allocation, including EQ5D, Hospital Anxiety and Depression Scale (HADS), Positive and Negative Affect Schedule (PANAS), Revised Illness Perception Questionnaire – Lung Cancer (IPQ-LC), Lung cancer risk perception, Health anxiety subscale of Health Orientation Scale (HOS) and the Lung Cancer Worry Scale (LCWS),

Medication, smoking behaviour, demographic details. Follow-up questionnaires include same items, plus Impact of Events Scale (intervention group only) and healthcare utilisation. The HADS is not included in follow- up questionnaires. Follow-up questionnaires are EQ-5D, cancer worry, positive and negative mood, smoking behaviour including cessation intentions and attempts; scores in additional questionnaires administered at between 1 and 24 months to subsets of the control arm and intervention arm; (all participants in the EarlyCDT-positive group will be approached with the recruitment aim of 300 from this group collected at 1,3,6,12,18 and 24 months. The EarlyCDT-negative and control groups will be recruited at the same rate as the EarlyCDT-positive group with the recruitment aim of 300 from each group collected at 1,3,6 and 12 months).

Three qualitative sub-studies will enquire via interview (telephone or face-to-face) to; 1) Investigate the experiences of individuals who choose not to have the EarlyCDT-Lung test rationale for not responding to a lung cancer screening, 2) Enquire how patients perceive the EarlyCDT-lung test and what do they understand about their test results and 3) Examine changes in smoking behaviour following EarlyCDT-Lung testing.

The recruitment and methodologies for these sub-studies are outlined in detail in Appendix 2.

(6) incidence at 24 months, and after 5 years and 10 years, in other clinical measures such as CVD, COPD, hospital stays, and outcomes identified through SMR linkage, etc. in the intervention arm and in the control arm; assessment of significance of differences;

(7) numbers in Tayside group (EarlyCDT-Lung test-positive, EarlyCDT-Lung test-negative, control) undertaking subsequent investigations such as CXR, CT, bronchoscopy, etc. Statistical modelling will be used to generate sample data for the Glasgow and Lanarkshire groups.

METHODS

Based on the test's 93% specificity and 41% sensitivity we anticipate that approximately 640 participants in the intervention arm will have a positive test result. These will be offered a chest X-ray. Those with a negative or indeterminate X-ray will be referred for a study CT scan. If the initial CT is negative then subsequent CTs will be offered 6 monthly for 24 months. Those individuals with monitorable abnormalities

as classified by the radiology/respiratory physician's study panel on baseline CT scan or subsequent CT will be followed up over the study period or referred for NHS clinical care as appropriate. All individuals entering the study will be flagged and followed up via the Scottish Cancer Registry. Participants who develop lung cancer will be individually followed-up via electronic record-linkage to assess both time to diagnosis and stage of disease at diagnosis. If no histological stage is available, stage will be assessed blind to allocation status from chest X-rays or CT, or, if no imaging is available a medical assessment of stage will be carried out.

HOW THE RESULTS OF THIS RESEARCH WILL BE USED

The study will assess the EarlyCDT-Lung test's clinical and cost effectiveness and suitability for a large-scale, accredited screening service for early lung cancer detection. It will also assess potential morbidity arising from the test and potential harms and benefits of a negative EarlyCDT-Lung test result.

DATES AND DURATION OF TRIAL

01/04/2013 –31-07-16 - End of recruitment (12k)

End of 24 month follow-up - 31/07/18 (+/- 4 weeks)

1. INTRODUCTION

1.1. BACKGROUND TO THE RESEARCH

Lung cancer is the world's leading cause of cancer related mortality and a major source of morbidity. 85% of patients with lung cancer remain undiagnosed until the disease is symptomatic and has reached an advanced stage. Moreover, Scotland has had one of the highest rates of lung cancer in the world. Around 2,460 men and 2,340 women are diagnosed with lung cancer in Scotland every year, which is 16% of the total UK lung cancer cases, despite Scotland having 8% of the UK's population. Survival from lung cancer is poor with less than 9% of patients still alive at five years after diagnosis, due primarily to late stage of presentation.

Early detection and diagnosis of cancer improves prognosis - the current 5-year survival rate is approximately 60% for stage I lung cancer but is only 1% for those with stage IV disease. The potential of early detection of lung cancer to improve outcomes was highlighted by the National Cancer Institute (NCI) National Lung Screening Trial (NLST) which recently reported that CT screening reduced lung cancer mortality by 20%. However as a primary screening modality CT is expensive and leads to substantial morbidity in a significant percentage of individuals whose tests are false positives. The EarlyCDT-Lung test is an innovative diagnostic test for early detection of lung cancer. The test can stratify individuals by risk of developing future lung cancer; those with a positive test are invited for a chest X-ray then, if that is normal, a CT scan. This targeted approach to CT scanning for early lung cancer detection is likely to be a more cost-effective and potentially less harmful approach to population screening than a blanket CT-scanning program of all people considered at high risk of future lung cancer.

A substantial body of published research has documented autoantibody (AAB) responses against various tumour derived/associated antigens (TAA) in patients with a wide range of solid tumours, including lung cancer. The serum proteome provides an attractive source of potential biomarkers and because serum collection is minimally invasive it can be repeatedly surveyed for cancer biomarkers. AABs have been detected months to years before clinical diagnosis of breast and lung cancers, supporting the hypothesis

that AABs could be incorporated into an early detection assay. Subsequent research studies have confirmed AABs to TAAs in patients with early stage lung cancer. AABs have been reported in lung cancer subjects up to 5 years before clinical diagnosis even where annual screening spiral CTs were being performed.

A serum assay has been developed and validated called Early Cancer Detection Test-Lung (EarlyCDT-Lung) that can detect 40% of lung cancers with a specificity of 90% by measuring autoantibodies to a panel of cancer antigens (p53, NY-ESO-1, CAGE, GBU4-5, Annexin1, & SOX2). Further confirmation of this sensitivity and specificity of the test for lung cancer using four new, independent sample sets has recently been published. A study of patient demographics showed no difference in autoantibodies based on age, gender and ethnicity. This autoantibody technology is different from CT scanning which in a prevalence screening test has a sensitivity of 67% for lung cancers developing over the following 12 months but with a low specificity of only around 49%. Indeed a prevalence CT screen will detect approximately 36% of the lung cancers which will develop in the next three years. If the EarlyCDT-Lung test has a three year 'look forward', as clinical data suggests, then the test will detect 40% of lung cancers which develop over this three year time period but with seven times fewer false positives than CT scanning. Two new autoantibodies (AAbs) have recently been added to the panel (and one removed) and the test now measures seven; p53, NY-ESO-1, CAGE, GBU4-5, HuD, MAGE A4 & SOX2 and identifies 41% of lung cancers with an increased specificity of 93% (Chapman et al; 2012). The 7-AAbs panel will be utilised in this study and all statistical calculations are based on the 41% sensitivity and 93% specificity of the Early CDT-Lung test.

EarlyCDT-Lung detects lung cancer at all stages – i.e. it detects early stage lung cancer as well as advanced disease - which means autoantibodies are present at all stages of disease. In a large group of patients with newly diagnosed lung cancers there was no difference in positivity rate for EarlyCDT-Lung in early or late stage disease lung cancers – whether this was looking at all lung cancers, only non small-cell (NSC) lung cancer, NSC lung cancer, or only small-cell lung cancer (SCLC). Thus, while autoantibodies are present in early stage they are not a biomarker of only early stage disease. An audit, (presented July 2011 at the

International Association for The Study of Lung Cancer) of the first 1000 patients to take the EarlyCDT-Lung test commercially, further confirms that the test works in clinical practice. These data are promising but an insufficient basis for introducing a national lung cancer screening program in the UK.

1.2. RESEARCH QUESTIONS

The primary research question is:

Does using the EarlyCDT-Lung test to identify those at high risk of lung cancer and any subsequent CT scanning reduce the incidence of patients with late-stage lung cancer (3 & 4) or unclassified presentation (U) at diagnosis, compared with standard practice?

Secondary questions include, but are not limited to:

i) is the use of the EarlyCDT-Lung test cost-effective compared to standard clinical practice?
ii) what is the emotional and behavioural impact of the EarlyCDT-lung test?
iii) does the EarlyCDT-Lung test improve clinical outcomes including but not limited to CVD, COPD, other cancers, hospital stays, outcomes identified through SMR linkage, etc.?

1.3. RATIONALE FOR STUDY

Lung cancer is the most common cause of cancer related death worldwide. The majority of cases are detected at a late stage when prognosis is poor.

CT scanning can reduce lung cancer mortality by 20%, but there are too many false positives leading to a large number of individuals without cancer being exposed to repeated unnecessary radiation.

A disproportionate amount of patients are given cause for concern when between 35%-75% of patients screened by CT are treated as positive (with resultant increased anxiety) but only 2%-3% will have a true cancer.

Active interventions (e.g. trans-thoracic biopsy, surgical resection) as the result of positive CT scans give rise to significant side effects and complications in a percentage of individuals.

The cost of screening with CT is expensive and unlikely to meet the thresholds for cost-effectiveness (£20k - £30k/QALY) usually used within the UK by bodies such as NICE.

Background to the study: pre-trial qualitative work

Four focus group sessions (Ethical approval by I-WHO, University of Nottingham, Appendix 1) were held with smokers aged 50 and over living in some of Glasgow and Dundee's most deprived areas in order to explore recruitment preferences and likely willingness to participate in the forthcoming EarlyCDT Lung Cancer Scotland (ECLS) Study.

The work was carried out throughout June and July 2012 in four areas of Scotland: Castlemilk, Darnley, Charleston and Douglas. A total of 32 people aged 50 – 75 took part in the work, including 14 men and 18 women. All but one were current smokers, and most had smoked for 40 years or more, smoking one pack or more per day.

The findings from the work enabled the formation of a number of recommendations for both the main trial recruitment strategy and materials, including:

1.3.1 Recruitment Strategy

- Adopting a personal approach to invitations, sent from GPs and followed up in writing;
- Setting deadlines for people to respond to invitations to maximise likely response rates, bolstered by local radio and newspaper coverage of the study;
- Providing early summary information which emphasises that the study is not focussed on trying to encourage people to stop smoking;

- Telling people which group they are in *after* taking their blood in order to minimise attrition during initial appointments but also emphasising the value of being in the 'non-test group' for the benefit of wider research and public health; and
- Offering flexible appointments that are close to people's homes.

1.3.2 Recruitment Materials

- Making sure that all documents explicitly say that the trial relates solely to lung cancer;
- Explaining the reasoning for the study design (including control and intervention groups) and setting out clearly what the inclusion/exclusion criteria are, and why these criteria apply;
- Explaining the purpose of randomisation, and ensuring early on that people know when they will be notified of which group they are in. This includes offering assurances that random means random and that being placed in the test group is not an indicator of risk;
- Acknowledging that not only smokers can be affected by lung cancer; and
- Offering sufficient information on the issue of making blood available to other researchers, and what this might entail, to allow fully informed consent to be given.

Trial documents have been developed based on the learning to emerge from these groups which will hopefully maximise participation in the upcoming trial and forearm those involved in its delivery as to the potential barriers to participation that may exist among the target population.

1.4. OBJECTIVES

Primary Objective

To assess the effectiveness of EarlyCDT-Lung test in reducing the incidence of patients with late-stage lung cancer at diagnosis compared with standard practice.

Secondary Objectives

1) to assess the effectiveness of EarlyCDT-Lung test in improving the diagnosis of early-stage lung cancers;
2) to undertake a cost-effectiveness analysis of EarlyCDT-Lung test as a primary screening method compared to standard clinical practice;

3a) to compare lung-cancer mortality, all-cause mortality and cancer-specific mortality in high-risk groups provided with EarlyCDT-Lung test, compared with standard practice;

3b) to compare long-term future mortality in high-risk groups provided with EarlyCDT-Lung test, compared with standard practice;

4) to obtain refined estimates of the sensitivity, specificity, positive predictive value and negative predictive value of EarlyCDT-Lung test;
5) to assess behavioural outcomes including smoking, psychological outcomes including cancer worry, anxiety, depression, distress specific to clinical investigations;
6) to assess the effectiveness of EarlyCDT-Lung test on other clinical outcomes;
7) to assess uptake of subsequent investigations.

1.5. OUTCOMES

Primary Outcomes

The difference, at 24 months after randomisation, between the number of patients with stage 3, 4 or unclassified lung cancer at diagnosis in the intervention arm, and those in the control arm;

Secondary Outcomes

1) numbers, at 24 months after randomisation, in the different stages at diagnosis (3/ 4/ U/ other) in the intervention arm and the control arm;
2) difference, after 2 years, between costs and outcomes in the intervention arm and in the control arm, cost-effectiveness of EarlyCDT-Lung test compared to normal clinical practice;

3a) estimates, after 2 years, of lung cancer mortality, all-cause mortality and cancer-specific mortality rates in the intervention arm and in the control arm; assessment of significance of differences;

3b) estimates, after 5 years and 10 years of long-term future mortality rates in the intervention arm and in the control arm; assessment of significance of differences;

4) estimates, after 2 years of (i) the number of patients with stage 3, 4 or unclassified lung cancer at diagnosis in the EarlyCDT-Lung test-positive group and those in the EarlyCDT-Lung test-negative group and (ii) stage at diagnosis in the EarlyCDT-Lung test-positive and EarlyCDT-Lung test-negative group;

5) scores at baseline, and follow-up on in a survey administered prior to treatment allocation, including EQ5D, Positive and Negative Affect Schedule (PANAS), Revised Illness Perception Questionnaire – Lung Cancer (IPQ-LC), Lung cancer risk perception, Health anxiety subscale of Health Orientation Scale (HOS) and the Lung Cancer Worry Scale (LCWS), Medication, smoking behaviour, demographic details. Follow-up questionnaires include same items, plus Impact of Events Scale (intervention group only), healthcare utilisation and dates and results of follow-up investigations for lung cancer (test positive group only). The HADS is not included in follow- up questionnaires. Follow-up questionnaires are EQ-5D, cancer worry, positive and negative mood, smoking behaviour including cessation intentions and attempts; scores in additional questionnaires administered at between 1 and 24 months to subsets of the control arm and intervention arm; (all participants in the EarlyCDT-positive group will be approached with the recruitment aim of 300 from this group collected at 1,3,6,12,18 and 24 months. The EarlyCDT-negative and control groups will be recruited at the same rate as the EarlyCDT-positive group with the recruitment aim of 300 from each group collected at 1,3,6 and 12 months).

6) incidence at baseline, 24 months, and after 5 years and 10 years, in other clinical measures such as CVD, COPD, other cancers, hospital stays, and outcomes identified through SMR linkage, etc. in the intervention arm and in the control arm; assessment of significance of differences;

7) numbers in Tayside group(EarlyCDT-Lung test-positive, EarlyCDT-Lung test-negative, control) undertaking subsequent investigations such as CXR, CT, bronchoscopy, etc. Statistical modelling will be used to generate sample data for the Glasgow and Lanarkshire groups.

2. STUDY DESIGN

2.1. STUDY DESCRIPTION

A randomised controlled trial involving 12,000 participants recruited through primary care and community based recruitment strategies in Scotland.

2.1.1. Setting

General practices in the lowest quintile of deprivation in Scotland as measured by the quintiles of the Scottish Index of Multiple Deprivation (SIMD 2012). Subsequent recruitment will be attained through adverts, posters, flyers and community based interactions. Potential participants can either be seen at their participating GP practice or at the local clinical research centre, or other appropriate clinical location.

2.1.2. Participants

Adults aged 50 to 75 who are at risk of lung cancer will be eligible to participate. These are defined as those who are, current or former cigarette smokers with at least 20 pack-years, or have a history of cigarette smoking less than 20 pack-years plus an immediate family history (mother, father, brother, sister, child) of lung cancer which gives an individual a personal risk similar to a smoking history of 20 pack years. Participants should be healthy enough to undergo pulmonary resection or stereotactic radiotherapy.

GP Invitation Letter/Study Introduction via alternative Recruitment Strategy

POSITIVE RESPONSE

- Send/email full PIS/PIB to non-GP recruitment strategy participants
- Make or receive call from participant
- Discuss study/check screening eligibility & arrange appointment

VISIT 1 @ GP PRACTICE OR CLINICAL RSEARCH FACILITY DUNDEE/GLASGOW) 30-45 MINS

See Research Nurse (RN) and obtain Informed Consent

Check inclusion/exclusion criteria

- Obtain 10 ml blood sample (for Early CDT test (0.5 mls)/cancer related medical research)
- Invite to complete study questionnaire (with assistance, if required)
- Web based randomisation-group allocation
- Remind participants that they will receive results in 4 weeks (test group only)
- Send Thank You letter (non-test group)

POST BLOODS TO UNIVERSITY OF NOTTINGHAM (UON)

- UoN process 10ml sample and send 0.5ml of test group sample to Oncimmune, Kansas, USA and store test and non-test blood for future cancer related medical research (with consent)

EARLY CDT TEST RESULTS

Research team obtains results via secure portal within 4 weeks of Visit 1

POSITIVE RESULT

Letter sent inviting participant to attend/call

Visit 2 @ GP/CRF (~30-45 mins)

- CXR/Study CT arranged
- Summary information given/posted to Participant (PIS 2)
- Participation & Results Letter sent to GP

NEGATIVE RESULT

- Results letter sent/offer to discuss

CXR & STUDY CT SCAN @ LOCAL HOSPITAL

- Participants attends for CXR
- If CXR negative participant attends for CT scan
- If CXR positive or suspicious participant informed to attend for NHS CT scan and seen by PI (Respiratory Physician) if required

(if NHS pathway negative, participant remains in study)

6 MONTHLY STUDY CT SCANS

- Participant given appointments to attend for Study CT scans every 6 months for 2 years

CLINICALLY SIGNIFICANT

CXR/CT SCAN/IMAGE INCIDENTIAL FINDINGS

- Results deemed clinically significant by review panel of radiologists/respiratory physicians will be followed up via NHS routine clinical care.

2.2. STUDY FLOWCHART

2.3. STUDY ASSESSMENT AND NOTIFICATIONS

Table 1. Overview of Study Assessments/Notifications.

ASSESSMENT/PROCEDURES	TIMELINE* (± 2 weeks)			
	Visit 1 (~30 -45mins)		Visit 2*(~30mns)	
Informed Consent	**X**			
Inclusion/Exclusion Criteria	**X**			
• *Review/Record only Relevant Medical History relating to IC/EC*	**X**			
• *Review/Record Relevant Medications*<br>• *Relating to IC/EC*	**X**			➢ **EarlyCDT Positive Test Participants may visit or call.*<br>➢ *EARLY CDT Negative Test Participants may attend for further information/advice only.*
Blood Sample	**X**			
Baseline Questionnaire	**X**			
Thank you letter to Control Group		**X**		
EarlyCDT- Lung Test Result Letter		**X**		
GP Results Letter & ICF copy (negative)		**X**		
Result Discussion/ Imaging Schedule			**X**	
Provide PIS 2			**X**	
GP Result Letter & ICF copy (positive)			**X**	

**EARLYCDT – Lung Test Positive Result Participants – Imaging Schedule**					
	TIMELINE(± 4 weeks) (±12 weeks for CT prior to study entry)				
	0	6 months	12 months	18 months	24 months
CXR	**X**				
*CT Scan**	**X**	**X**	**X**	**X**	**X**
***Scheduled every 6 months, if participant enters NHS clinical care pathway, subsequent study CT scans will be cancelled.**		***Research team member will call 2-4 days before each scheduled CT scan to check health status and attendance.***			

3. STUDY POPULATION

3.1. NUMBER OF PARTICIPANTS

Twelve thousand participants from general practices in the most deprived quintile of the population Scotland (as measured by the quintiles of the Scottish Index of Multiple Deprivation (SIMD) 2012 - version 2. In this second phase of recruitment, from 10,000 to 12,000 only participants from NHS Greater Glasgow and Clyde and NHS Lanarkshire will be invited as recruitment in NHS Tayside is now complete.

3.2. INCLUSION CRITERIA

1. Participant is willing and able to give informed consent for participation in the study
2. Male or female aged 50 years to 75 years
3. Current or Ex-smoker with at least 20 year pack history
4. or Less than 20 year pack history but with family history of lung cancer in a 1st degree relative (mother, father, sister, brother, child)
5. ECOG Status: 0, 1 and 2 (Eastern Co-operative Oncology Group)

Grade	ECOG
0	Fully active, able to carry on all pre-disease performance without restriction
1	Restricted in physically strenuous activity but ambulatory and able to carry out work of a light or sedentary nature, e.g., light house work, office work
2	Ambulatory and capable of all self-care but unable to carry out any work activities. Up and about more than 50% of waking hours

6. Geographical postal sectors of:

NHS Geographical Area	Eligible Postcodes
**Tayside**	DD1 - DD11, PH1–PH3 , PH6-PH8, PH10, PH11, PH13, PH15 & PH16, KY13
**Greater Glasgow & Clyde**	G1-G5, G11 –G15, G20-G22, G31-34, G40 –G46,<br><br>G51- G53, G60-G62 &G64, G66 & G69, G72 & G73,<br><br>G76-G78, G81-G83<br><br>PA1–PA8 (except PA6), PA11-PA16 & PA19
**Lanarkshire**	G33, G65, G67, G69, G71-75, ML1-12

3.3. EXCLUSION CRITERIA

1. History of any cancer other than non-melanomatous skin cancer, cervical cancer in situ.
2. Symptoms suggestive of lung cancer within past 6 months (haemoptysis, unintentional weight loss (at least 5% in preceding 6 months).
3. Patients for whom the GP considers invitation to the study would cause undue distress.
4. Patients with other terminal disease.
5. Patients on (> 3months) of Cyclophosphamide . Note: Other prolonged / continuous use (>3months) of cytotoxic/ immuno-suppressant drugs eg: Methotrexate, Azathioprine, Rapamycin, Mycophenolate, Rituximab and anti-immunophilins such as Ciclosporin, Tacrolimus,and Monotherapy using glucocorticoids/ steroids eg prednisolone are NOT exclusion criteria.

4. PARTICIPANT SELECTION AND ENROLMENT

4.1. IDENTIFYING PARTICIPANTS

Potentially eligible individuals will be identified from GP medical records by an electronic medical record search undertaken by the Scottish Primary Care Research Network. Potential participants will be recruited via their General Practitioner (through SPCRN) using a range of methods including:

a. postal invitation letter including a Participant Information Brochure;

   and where necessary or appropriate:

b. invitation letter including a summary of the study Participant Information Sheet on collection of repeat prescription;

c. invitation during consultation with GP / Practice Nurse / Health Care Assistant at the practice;

d. invitation to those eligible on registered research volunteer databases

e. poster present in the GP's waiting room;

f. other recruitment strategies may be employed including; Media campaign involving: local and national newspapers; BBC Scotland; local radio,

   Celebrity endorsement

g. Publicity campaign using posters/leaflets etc….including:

   Football/Bingo halls/ Bowling

   Smoking Cessation Clinics

   Hospital main entrances/ hospital clinics

   Shopping Centres/Supermarkets/Pubs/etc.

   Benefits offices/Post offices etc.

   Sheltered Housing /Housing Associations

   Community and charitable outreach programs

   Mobile screening clinic

   Pharmacist approach through practices.

The potential impact of the presentation of the PIB on recruitment (rather than understanding) is unclear and is being evaluated by the embedded MRC START study (http://www.medicine.manchester.ac.uk/mrcstart/about/). ECLS has l included an embedded methodological substudy (substudy 4) of two, ethically-approved PIB presentations as part of the START study. The full protocol of sub-study 4 is given in Appendix 3. (The START (sub-study 4) now completed).

For this sub-study, potential participants received one of two versions – the original version (ECLS PIB) or a revised version (START PIB), which has had both its language and its design modified after consultation with groups of the public selected to be similar to the ECLS target population. The content (i.e. the topics covered) remains the same in the both versions of the Participant Information Brochure. The allocation of brochure sheet version to each participant was decided randomly. The main outcome of interest here is the proportion of participants receiving each version of the sheet who go on to take part in the ECLS trial. When a participant is consented to the ECLS Study they will indicate that they have read and understood either the ECLS PIB or the START PIB. A sample size of approximately 2000 participants was involved in MRC START in ECLS, split equally between the two versions of the PIB and GP Invitation Letters. Sub study 5 is the work of an MRC funded PhD studentship at the University of Glasgow. The proposal adds two new aspects to the psychological sub-studies. Firstly looking at people who decide not to take part after showing interest by either cancelling or not attending their appointment, and exploring why they change their mind. Secondly exploring any differences between the people taking part who self refer for the study as opposed to reply to a GP invitation. Full details of the sub-study can be found in Appendix 4.

The study invitation letter will include a slip for participants to either express interest in finding out more about the study, provide their contact details or to request no further contact about

the study. Those returning an expression of interest will be telephoned, more than 24 hours after anticipated receipt of the Participant Information Brochure, by a member of the research team. Additionally, the participant is given the opportunity to call or email the study team. The call (instigated by participant or study team) will allow a discussion of the study, to answer any questions the potential participant may have, do a preliminary assessment of eligibility and if they agree, to make an appointment for a recruitment visit (hereafter referred to as the eligibility assessment phone contact). An appointment letter/email will be sent out to confirm appointment.

A reminder call/email or text, whichever is preferable to the participant, will be carried out up to 2 days prior to the screening appointment. A reminder process decreases non-attendance. Non-responders to the GP postal invite will be approached again using a reminder letter or postcard. Those participants who have not responded after the first reminder will be viewed as non-responder and eligible for the first qualitative sub-study (See Appendix 2). This study will investigate the experiences of individuals who choose not to respond to lung cancer screening. If GPs agree, a member of the ECLS research team will attend the practice and call non-responders as per the eligible SPCRN list. This process has proven to be a successful reminder methodology in previous primacy care research conducted by the CI. A member of the research team will undertake the eligibility assessment phone contact for those expressing interest in the consultation or by returning the expression of interest form at a later date.

The recommended study visit order (findings from focus groups) is:

- obtain consent
- take bloods from all consented participants (in the unlikely event; a blood sample is unobtainable or the blood sample blood sample from a participant in the test group is lost during transportation the participant will be contacted to arrange a subsequent sample.)

- ➢ complete survey questionnaire
- ➢ randomise to treatment arm

After randomisation group allocation is known all participates will be asked if they continue to be happy for their bloods to be used for the Early CDT- Lung Test (lung cancer test group) and for future cancer related research for those who agreed by initialling the relevant box on the consent form.

For participants randomised to the intervention arm the EarlyCDT-Lung test will be performed and patients followed up according to their result.

At Visit 1 participants are advised that those with a positive EarlyCDT-Lung test result will be invited to a follow-up visit to interpret the test results and explain the progress on study thereafter. Those with a negative EarlyCDT-Lung test result will be written to, explaining the test results and will be offered a follow-up visit or a telephone call if they wish. They will be advised of symptoms to watch for including persistent cough, coughing up blood, shortness of breath, weight loss or loss of appetite. They will be counselled to carry on having tests for other types of cancer if they are offered (e.g. bowel cancer test, mammograms, cervical smears). In less than 1% of cases an Early CDTLung test is deemed *invalid.* The test panel for the participant shows a characteristic indicative of some interfering nonspecific immunoreactive component. This result is deemed neither positive nor negative and it is not recommended that the test is repeated as the result will remain invalid. On those very rare cases that this occurs a participant will be informed by a telephone call and a follow-up results letter. Those in the control arm will be written to and thanked for their contribution to the study and advised and counselled identically to those with a negative test result.

All participants who agreed to donate blood for future will be advised that it will be used in cancer related research.

A patient specific section of the study website (www.eclsstudy.org) containing Participant Information Sheets and research staff contact details will be available for participants to view.

4.2. CONSENTING PARTICIPANTS

All individuals taking informed consent will have received training in Good Clinical Practice (GCP). It will be explained to patients that they are under no obligation to enter the trial and that they can withdraw at any time during the trial, without having to give a reason. A copy of the signed Informed Consent Form (ICF) will be given /or posted out to the study participant. A copy of the signed consent form will be given or sent to the GP with a letter outlining the study and patient pathway. The letter will notify the GP of their patients' group allocation, relevant Early CDT-Lung test result, and any notable findings found at the screening visit, namely, the request to give up smoking or relevant clinical information that requires further clinical judgement. The original copy of the ICF is to be retained at the study site (ISF or TMF, as appropriate.). If any notable findings are found at the screening visit an anonymised copy of the GP letter will be filed in the participant's study file. If new safety information results in significant changes to the study risk–benefit assessment, the Protocol, Participant Information Sheet and/or consent form will be reviewed, updated and amended as necessary. All participants will be informed of the new information, given a copy of the revised consent form and asked to re-consent if they choose to continue in the study.

4.3. SCREENING FOR ELIGIBILITY

SPCRN staff will visit practices to undertake searches of the GP computerised records. The resulting list of potentially eligible participants will be checked by a GP at the practice to ensure that those for whom study participation would cause undue distress and those with a terminal disease are not sent

study invitations. Telephone screening of potentially eligible participants who have returned an expression of interest will be undertaken by a member of the research team at the eligibility assessment phone contact. This will include assessment of age, smoking history, family history of lung cancer, previous cancer, ECOG status and eligible postcode.

4.4. INELIGIBLE AND NON-RECRUITED PARTICIPANTS

The reason(s) for ineligibility will be explained to the patients and any questions they have will be answered. They will be thanked for their interest in the trial and any relevant clinical information will be communicated to their GP where the patient has given consent.

4.5. RANDOMISATION

Participants will be allocated to intervention or comparison group during the recruitment visit (Visit 1) using a web-based randomisation system; TRuST, provided by Tayside Clinical Trials Unit (TCTU). Set-up of the randomisation system will be by TCTU staff under the supervision of a TCTU statistician. Randomisation will be stratified by site and minimised by age, sex and smoking history.

4.6. ADMINISTRATION OF THE TEST

Individuals at higher risk will be identified from GP medical records or community based recruitment as described above. Consenting individuals will be randomised to either receive an EarlyCDT-Lung test or standard care.

4.7. MANAGEMENT OF THE VISITS

Based on the test's 93% specificity and 41% sensitivity we anticipate that approximately 640 participants in the intervention arm will have a positive test result. These will be offered a chest X-ray. Those with a negative or indeterminate X-ray will be referred for a CT scan. If the initial CT is negative then subsequent CTs will be offered 6 monthly for 24 months.

If a participant has had a chest X-ray in the previous 1 month, or a CT scan in the previous 3 months to a scheduled study CT scan, these can be reviewed as part of the study. This will reduce the need to expose participants to unnecessary radiation. With the participants consent chest X-rays or CT scans prior to study entry will be retrospectively coded as per ECLS Radiology Schema. The participant will proceed to have the series of 5 CTs if clinically appropriate.

Participants will receive appointments via mail/email (as preferred). Participants will be called 2-4 days before each CT-scan appointment. By calling, this allows the participant to ask any questions, check health status, arrange transport (if required) and increase participant retention. Individuals with monitorable abnormalities as classified by the radiology/respiratory physician's study panel on baseline CT scan or subsequent CT will be followed up over the study period or referred for NHS clinical care as appropriate. All individuals entering the study will be flagged and followed-up via the Scottish Cancer Registry. Participants who develop lung cancer will be individually followed-up via their medical records to assess both time to diagnosis and stage of disease at diagnosis. If no histological stage is available, stage will be assessed by a panel of three respiratory physicians blind to allocation status of the study subjects from chest X-rays or CT, or, if no imaging is available, medical assessment of stage will be carried out.

Prior to sending CT scan appointments participant deaths will be check using the SMR to ensure sensitivity is maintained. All participants in the EarlyCDT- Positive test groups known to have died will be removed from the CT scan appointment schedule register. If patients (EarlyCDT-Positive test) fail to attend for any imaging assessment during the study, they will receive two reminders (one letter, one phone call). On the third non-attendance, a letter will be sent to the participant's GP to inform them of non-attendance. An appointment window of ± 4 weeks will be initiated for each scheduled CT scan.

Participants will receive results letters in relation to their initial CXR and CT scan and subsequent CT scans. Any clinical intervention/treatment will be arranged by the study team.
Two additional sub- studies 6 and 7 (Appendix 5 & 6, respectively) have been added to establish difference in the emotional, cognitive and behavioral response to a positive EarlyCDT test if pulmonary nodules are present on a chest computed tomography compared to a normal chest computed tomography?

This study will utilise anonymised data from study participants in the EarlyCDT-positive group who participated in the emotional and behavioural outcomes study and completed the baseline questionnaire and at least one follow-up questionnaire at one, three or six months post recruitment (Qualitative Sub-Studies, Appendix 2). Participants will not require to be contacted for this sub-study. Full proposal is described in Appendix 5.

Sub- study &: Living with lung nodules: what information would patients find helpful?

The aim of this sub-study study is to explore the ECLS study participant response following receipt of a letter informing them that their CT scan showed a pulmonary nodule.

The letter currently used in the trial is based on that used in routine clinical care. It is important to explore patient's experiences following receipt of the letter to inform any roll out of lung cancer screening as a national programme. Through the use of focus groups the existing letter will be reviewed and, if deemed appropriate, modifications will be made with a view to improving how these test results are communicated.

The objectives of this study will be achieved through the use of four different focus groups. Groups 1 and 2 will focus on the current experiences of the ECLS trial participants receiving notification of a lung nodule while Groups 3 and 4 will seek to address what the participant response would be to a modified information letter which would be developed (if changes are required) from the comments from focus Groups 1 and 2.. A full outline of the project is outlined in Appendix 6.

4.8. WITHDRAWAL AND STUDY TERMINATION PROCEDURES

No circumstances are anticipated for the withdrawal of patients from the trial initiated by the clinical team or trial investigators. Patients may choose to withdraw from the trial at any time, without giving a reason, and without compromising their future treatment.

If the study should be terminated early, for whatever reason, all participants with a positive Early CDT-Lung test will continue to be seen by the PI (lung specialist) and will continue to undergo any clinically relevant investigations and reviews and will be treated (if required) according to current clinical practice.

5. STUDY AND SAFETY ASSESSMENTS STUDY ASSESSMENTS

The main study assessment is the test result.

Other assessments include;

i. diagnosis
ii. costs associated with intervention including any follow up/confirmatory tests and subsequent treatment
iii. costs associated with routine clinical management of patients
iv. health utility data
v. mortality (various)
vi. measures of psychological outcomes and health behaviour
vii. other clinical outcomes uptake of subsequent investigations

5.1. SAFETY ASSESSMENTS

As the study does not employ an Investigational Medicinal Product, Adverse Events (AE) or Serious Adverse Events (SAE) will be recorded but not reported in the Annual Report. A number of factors affecting the trial population suggest that we would expect to observe a larger than normal incidence of episodes of ill-health due to both the age and co-morbidities of the study population. All known disease progressions and co-morbidities will be regarded as outcomes including complications arising from investigations which result in a hospital stay which will be captured in outcome 6 and all medical treatment or interventions will be predicated upon normal clinical care and not related to the study protocol. All CXR and CT scan incidental findings (incidentaloma) will be recorded in the CRF as an incidental findings as per Radiological reporting Schema and Study Operations Manual. Bespoke letters to GPs and/or specialist referrals will be completed by study

physicians as required. Participants will be informed of any incidental findings and any action required by a study physicians via letter or phone call if appropriate.

6. DATA COLLECTION & MANAGEMENT

6.1. DATA COLLECTION

It is the CI and PIs responsibility to ensure the accuracy of all data entered and recorded in the CRF/eCRFs and the database. The Delegation of Responsibilities Log will identify all trial personnel responsible for data collection, entry, handling and managing the database.

The data will be collected by the RN and/or the PI directly onto a paper CRF with subsequent transcription to the eCRF. Where there is electronic storage of non-identifiable data this will be on a password protected device and/or database. A plan for data quality control will be developed by the data management staff at Tayside Clinical Trials Unit and the trial management team.

All research blood samples (anonymised using barcodes) will be labelled and packaged according to IATA regulations using Royal Mail Safeboxes or INTELSIUS or equivalent transport box systems to be transported to the University of Nottingham for processing, transporting and storage of samples for future research. All samples will be stored under custodianship as per UK Biobank guidelines. Sample Analysis and Chain of Custody Plans will be documented in the Study Operations Manual.

The participant's medical notes (GP and hospital) paper or electronic will act as source data for relevant past medical history, subsequent medical conditions, hospital admissions and diagnostic reports.

Psychological and behavioural data relating to smoking, psychological outcomes including cancer worry, anxiety, depression and distress will be collected on all 12,000 participants through a baseline questionnaire administered during Visit 1. If required the RN can assist the participant with the completion of the questionnaire. Follow-up data will be collected between 1 and 24 months on subsets of the intervention and control groups. All participants in the EarlyCDT-positive group will be

approached with the recruitment aim of 300 from this group collected at 1,3,6,12,18 and 24 months. The EarlyCDT-negative and control groups will be approached at the same rate as the EarlyCDT-positive group with the recruitment aim of 300 from each group, collected at 1,3,6 and 12 months.. A web-based tool will be used weekly to randomly sample patients from the EarlyCDT-negative and control groups, stratified by the two study centres. It is anticipated an average of 8 individuals (4 from each of the two study centres) will be randomly sampled and invited to complete follow-up questionnaires from each of the EarlyCDT-negative and control groups per week (based on a 10 month recruitment period and an anticipated response rate of 67%). Response rates will be monitored and if they are lower than 67%, the number randomly sampled at each centre will be increased to achieve a minimum of 200 responses. Participants will be sent a £5 gift voucher for use in a range of stores for each questionnaire to be completed. A Cochrane review found the use of small monetary incentives significantly increases response rates to postal questionnaires (Edwards et al., 2009). There are precedents in trials and other UK studies where small monetary incentives have been used.

Two methods will be used for the initial period of recruitment: 50% of the sample will receive the questionnaire with the voucher and 50% will receive the voucher once they have returned their questionnaire. An assessment will be carried out to determine which of the two methods is more effective in maximising recruitment rate and will then be employed for the remainder of the study. A phone number will be provided for participants to call the research team for assistance in completing questionnaires. Occasionally the research team may call participants to check on postal delivery and offer assistance with completion to increase return rate. Participants who develop lung cancer during the 24 month follow-up period will not be sent further study questionnaires.
For those participants who have agreed to further contact will be eligible for qualitative sub-study 2 and 3 as outlined in Appendix 2.

Follow up questionnaire sampling to achieve 300 participants in control, negative and positive groups is now complete. No participants will be actively recruited to these sub-studies. However, those participants in the positive group will be invited to continue to complete the follow-up questionnaires to 24 months post randomisation.

6.2. DATA MANAGEMENT SYSTEM

Tayside Clinical Trials Unit (TCTU) will provide a data management system using OpenClinica (https://www.openclinica.com/), its standard GCP-compliant data management system. Case Report Forms (CRF) will be developed together with the trial management team, statistician and data manager to ensure that the data management system supports the research aims of the study. The data management system will be fully validated, including the provision of test data and supporting documentation. Data entry will be coordinated by TCTU. Data will be stored on servers controlled through the Tayside Medical Science Centre and housed within the Health Informatics Centre and the University of Dundee. Backup and disaster recovery will be provided by TCTU according to its standard operating procedures.

The Statistical Analysis Plan will specify dummy tables linked to primary and secondary outcomes and the data management system will be designed to export directly to the dummy table formats for analysis.

7. STATISTICS AND DATA ANALYSIS

7.1. SAMPLE SIZE CALCULATION

The rate of lung cancer is 187/100,000 per year for patients aged 50-74 in Scotland 2008 (ISD cancer statistics). Deprivation is associated with a higher risk of lung cancer. Those in the most deprived quintile are associated with an increased risk of 1.8 times compared to the middle quintile of deprivation. (ISD cancer statistics) this gives an estimated annual lung cancer rate of 336/100,000 among the practices taking part in the study. A high risk group within this population will be selected using similar entry criteria (outlined above) as the Mayo screening study which had a 2% prevalence rate of lung cancer and a further 2% incidence rate over the following 5 years (Swensson 2005). The baseline rate of late stage presentation for the particular high risk population envisaged in this study is uncertain, as is the size of the reduction in late stage presentation likely to be achieved through use of EarlyCDT-Lung. Using an estimated late stage presentation rate of 1,200/100,000 per year in the control group i.e. 2.4% over the two-year follow-up period, we require 85% power at 5% significance (two-sided) to detect an estimated reduction of 35% in presentation rate in the test group i.e. as low as 780/100,000 per year or 1.56% over the two-year follow-up period. This corresponds to an estimated event rate over the two years of follow-up of 120 events in the control group and 78 events in the test group and implies a required sample size of n=5,000 per group i.e. 10,000 altogether.

The anticipated 35% reduction in event rate between the control group and the test group is justified by current estimates of the capability of the EarlyCDT-Lung test to identify cases (41% sensitivity, 93% specificity) together with current estimates of the specificity of CT scanning (67%). The sample size calculations are based upon standard methods for time to event data using the cpower function in R and stpower exponential procedure in Stata and assuming exponential survival. They were also confirmed using standard approaches for detecting a change in binomial

probabilities, and confirmed using approaches to detect a change in Poisson rates (with essentially identical results as loss to follow up is expected to be low).

The study aims for a short recruitment period and so no allowance has been made for accrual. With such an allowance, say to 1 year, the power will increase to 91% to identify a 35% reduction provided the minimum follow up period of 2 years is observed.

The initial assumptions of the rate of late stage presentation rate of 1,200/100,000 per year among the study participants was too optimistic and in January to May 2015 investigations were carried out to inform an increase in the sample size. Baseline information on the 8639 participants recruited to March 2015 (18 months from first randomisation) was used to derive an estimate of lung cancer risk based upon the Spitz Model (25). A number of variables in this model were not recorded in the study data base and low risk values were used in the risk calculation implying that the risk estimates should be underestimates. This suggested that the with 10,000 participants the rate of lung cancer would be expected to be around 680/100,000 and 540/100,000 for stage T3/T4/Unknown lung cancer using ISD cancer statistics figures of 80% lung cancers in Scotland are late stage. A sensitivity analysis around the missing data assumptions suggests that a late stage rate of around 600/100,000 may not be unreasonable, though is likely to be at the upper limit.

Using an assumption of 600/100,000 for late stage lung cancer and acknowledging that recruitment is over a 2 year period the study has a power of 80% to detect a 35% reduction associated with the use of the EarlyCDT-Lung test to identify cases, provided that analysis takes place after all randomised patients have been followed up for 2 years. While an 80% power is at the lower end of acceptable powers this is the power level which has been used in a number of lung cancer screening trials.

The power of the study is sensitive to the assumptions about the rate of late stage cancer and the recruitment rate, see Table 2. A power in excess of 90% could only realistically be achieved by recruiting 15,000 patients or by changing the primary endpoint to 3 years post randomisation for all patients. It the recruitment phase extends past 2 years to 2.5 years to recruit 12,000 participants then the power will increase slightly to 83%.

Table 2. Powers for a 35% reduction in the rate of T3/T4/Unknown lung cancer using a log rank test at the 5% significance level for various underlying rates in the control group, total sample sizes, and differing lengths of recruitment periods and follow up periods.

Recruitment		2 Years	2.5 Years	3 Years	2 Years
Follow Up		2 Years	2 Years	2 Years	3 Years
Rate T3T4/Unk	Sample Size				
500	10000	0.649	0.683	0.715	0.771
	12000	0.727	0.761	0.791	0.841
	15000	0.818	0.847	0.872	0.911
600	10000	0.727	0.761	0.791	0.841
	12000	0.802	0.832	0.858	0.9
	15000	0.881	0.905	0.924	0.952
700	10000	0.791	0.822	0.848	0.892
	12000	0.859	0.884	0.906	0.938
	15000	0.924	0.942	0.956	0.975

For the follow-up analysis of behavioural and psychological outcomes, 200 participants in each group will allow a mean difference of 3.00 (SD 15.04 (unpublished data from the ProtecT prostate cancer study) in the Impact of Events Scale with 80% power and 2-sided 5% significance level. We will, however, collect data from 300 patients in each group to allow for attrition. Assuming 80% participants are current smokers, we will obtain 80% power at 5% significance level to detect a reduction in smoking from 80% to 67% i.e. 13% points difference assuming follow up on 200 participants.

7.2. PROPOSED ANALYSES

Characteristics of participants will be compared informally between treatment arms at baseline. The main analysis of the primary outcome will be intention-to-treat. Cox proportional hazards models will be used to estimate the hazard ratio of the rate of late stage lung cancer in the intervention arm compared to the control arm. Participants who are lost to follow up will be censored. The models will adjust for age, gender smoking history and practice. If appropriate, random cluster effects will be included rather than fixed effects for practices. A similar methodology will be used for the secondary outcomes of comparisons of mortality rates (secondary outcomes 3a and 3b). A subsequent analysis will compare the outcomes of those with EarlyCDT positive in comparison to those in the intervention group with EarlyCDT negative (primary contrast for this analysis) and those in the control group (secondary analysis 1). Comparisons of proportions (secondary analyses 1 and 4) will be carried out using chi square tests. Fishers exact test will be used if the numbers of events are small.

The analyses of the questionnaire responses (secondary analysis 5) will be carried out using the appropriate 2 sample t tests and regression methods at baseline. Non parametric tests will be used if there is evidence of non-normal scores. Multilevel models will be used to analyse the repeated scores during follow up.

Poisson regression models, adjusting for follow up time if necessary, will be used to investigate the other clinical measures (secondary outcomes 6 and 7).

7.2.1. Cost effective analysis

The short-term within-trial analysis will compare the costs and outcomes associated with the intervention group to those of the comparison group at 24 months. A longer term analysis will employ a decision analytic model to link the short term outcomes measured within the trial to

potential longer term impacts on health (for example in terms of impacts on the development of cardiovascular disease, diabetes etc.). Both analyses will utilise the NHS and personal social service perspective favoured by NICE.

7.3. MISSING DATA

The extent of missing data will be examined and, if necessary, methods such as multiple imputation will be implemented to provide robust results, assuming data are missing at random (MAR).

7.4. TRANSFER OF DATA

Transfer of Data will be achieved according to standard TCTU SOPs (Study Operations Manual).

7.5. PREGNANCY

The female age group in this study (50 to 75 years) are unlikely to be pregnant. However, assessment of risk is established when all women are asked about the possibility of pregnancy prior to any imaging investigations as per usual NHS risk assessment protocols.

8. TRIAL MANAGEMENT AND OVERSIGHT ARRANGEMENTS

8.1. TRIAL MANAGEMENT GROUP

The trial will be overseen by a Trial Steering Committee (TSC) and a Trial Management Group (TMG) consisting of the CI and co-investigators, trial managers and with representation for research nurses and SPCRN. Day-to-day management of delivery of the trial will be achieved through the Trial Operations Group chaired by the Assistant Director of TCTU and comprising project and trial managers, data managers, statistician and software developers.

8.2. TRIAL MANAGEMENT

The Senior Trial Manager and Trial Manager will oversee the study and will be accountable to the CI. The Senior Trial Manager will be responsible for other trial processes hosted within the TCTU. However, this remains the overall responsibility of the CI. Any queries will be resolved by the CI or delegated member of the trial team.

A study-specific Delegation Log will be prepared for each site, detailing the responsibilities of each member of staff working on the trial.

8.3. TRIAL STEERING COMMITTEE

The committee will be a TSC with an integrated data monitoring committee. It will be a mixture of lung cancer investigators and independent members. The TSC will be chaired by an independent, expert in cancer research and clinical trials. Other independent members will include a statistician. The TSC will meet bi-annually, with the first meeting being shortly after the start of the project. The terms of reference of the TSC and the draft template for reporting will be detailed in the ECLS TSC Terms of Reference.

8.4. INSPECTION OF RECORDS

The CI, PIs and all institutions involved in the study will permit trial related monitoring, audits, REC review, and regulatory inspection(s). In the event of an audit, the CI agrees to allow the Sponsor, representatives of the Sponsor or regulatory authorities direct access to all study records and source documentation.

8.5. RISK ASSESSMENT

A pre-Sponsorship study risk assessment was carried out by the TASC Research Governance Manager prior to Sponsorship approval being granted.

8.6. STUDY MONITORING

The Sponsor will determine the appropriate extent and nature of monitoring for the study and will appoint appropriately qualified and trained monitors.

8.6.1 Potential Risks

8.6.2 Blood sampling

Veins and arteries vary in size from one patient to another and from one side of the body to the other. Obtaining a blood sample from some people may be more difficult than from others. Risks associated with having blood drawn are slight but may include:

- Excessive bleeding
- Fainting or feeling light-headed
- Hematoma (blood accumulating under the skin)
- Infection (a slight risk any time the skin is broken)

All research nursing staff will be highly trained and experienced in venipuncture thereby minimizing risk.

8.6.3 Test results

False positives and false negatives are explained as follows:

No medical test is completely accurate. This blood test is expected to pick up about 40 in 100 cases of lung cancer and detect the cancer at an early stage. However this means it doesn't pick up all cases of lung cancer. So even if your test is negative, or if you are in the non-test group, it is important that you see your GP if you are unwell in any way that could be due to lung cancer. This includes persistent cough, coughing up blood, shortness of breath, weight loss or loss of appetite. As no medical test is completely accurate, the blood test will be positive in some people who do not have early lung cancer (false positive). These people will be offered a chest X-ray and lung scans to see if they have lung cancer. We expect this to happen to 8 out of every 9 people who have a positive test result.

8.6.4 Radiography

Risks relating to chest X-ray and CT scan are explained as follows:

Chest X-rays and lung scans use radiation. People can develop cancer because of this radiation, but this is very rare. The amount of radiation you get from a chest X-ray is very small. About 1 million people would need to have a chest X-ray for one extra person to develop cancer because of the chest X-ray. A CT lung scan gives about 600 times as much radiation as a chest X-ray. 1500 people would need to have a CT lung scan for one extra person to develop cancer because of the scan. These risks are very small compared to the one in four chance we each have of developing cancer in our lifetime. Only about 640 people in this study are expected to have a positive blood test and will, therefore, need chest X-rays and scans. The chances of radiation affecting anyone in this study in this way are therefore very small.

8.6.5 Minimising Risk

All associated risks are well understood and have established procedures for management.

9. GOOD CLINICAL PRACTICE

9.1. ETHICAL CONDUCT OF THE STUDY

The study will be conducted in accordance with the principles of good clinical practice (GCP) and the Research Governance Framework Scotland.

In addition to Sponsorship approval, a favorable ethical opinion will be obtained from an appropriate REC and appropriate NHS R&D approval(s) will be obtained prior to commencement of the study.

9.1.1. Confidentiality

All laboratory specimens, evaluation forms, reports, and other records will be identified in a manner designed to maintain participant confidentiality. All records will be kept in a secure storage area with limited access to study staff only. Clinical information will not be released without the written permission of the participant, except as necessary for monitoring and auditing by the Sponsor, its designee or Regulatory Authorities. The CI and study staff involved with this study will not disclose or use for any purpose other than performance of the study, any data, record, or other unpublished, confidential information disclosed to those individuals for the purpose of the study. Prior written agreement from the Sponsor or its designee will be obtained for the disclosure of any said confidential information to other parties.

9.1.2. Data Protection

The CI and study staff involved with this study will comply with the requirements of the Data Protection Act 1998 and subsequent General Data Protection Regulation updates, with regard to the collection, storage, processing and disclosure of personal information and will uphold the Act's core principles. The CI and study staff will also adhere, if appropriate, to the current version of the NHS Scotland Code of Practice on Protecting Patient Confidentiality. Access to collated participant data will be restricted to the CI and appropriate study staff.

Computers used to collate the data will have limited access measures via user names and passwords. Published results will not contain any personal data that could allow identification of individual participants.

9.1.3. Insurance and Indemnity

The University of Dundee and Tayside Health Board are Co-Sponsoring the study.

Insurance. –The University of Dundee will obtain and hold Professional Negligence Clinical Trials Insurance cover for legal liabilities arising from the study.

Tayside Health Board will maintain its membership of the Clinical Negligence and Other Risks Insurance Scheme ("CNORIS") which covers the legal liability of Tayside in relation to the study. Where the study involves University of Dundee staff undertaking clinical research on NHS patients, such staff will hold honorary contracts with Tayside Health Board which means they will have cover under Tayside's membership of the CNORIS scheme.

Indemnity. The Co-Sponsors do not provide study participants with indemnity in relation to participation in the Study but have insurance for legal liability as described above.

10. STUDY CONDUCT RESPONSIBILITIES

10.1. PROTOCOL AMENDMENTS, DEVIATIONS AND BREACHES

The CI will seek approval for any amendments to the Protocol or other study documents from the Sponsor, REC and NHS R&D Office(s). Amendments to the protocol or other study docs will not be implemented without these approvals.

In the event that a CI needs to deviate from the protocol, the nature of and reasons for the deviation will be recorded in a Protocol Deviation & Breach Log and Protocol Deviation & Breach Report (if required) and submitted to the Sponsor (if required). If this necessitates a subsequent protocol amendment, this will be submitted to the Sponsor for approval and then to the appropriate REC and lead NHS R&D Office for review and approval.

In the event that a serious breach of GCP is suspected, this will be reported to the Sponsor immediately using the form “Notification to Sponsor of Serious Breach or Serious Deviation”.

10.2. STUDY RECORD RETENTION

To enable evaluations and/or audits from regulatory authorities, the investigators agree to keep records, including the identity of all participating patients (sufficient information to link records, all signed informed consent forms, source documents, and group allocation to intervention and control). The records should be retained by the study site coordinators and investigator according to TASC SOP or local NHS Board regulations, or as specified in the Clinical Study Agreement, whichever is longer.

If the CI, PI or a study site coordinator relocates, retires, or for any reason withdraws from the trial, the University of Dundee should be prospectively notified. The trial records must be transferred to an acceptable designee. The study site coordinator must comply with the TASC SOP on archiving and

obtain written permission from the Sponsor before disposing of any records, even if retention requirements have been met.

10.3. END OF STUDY

The end of study is defined as last patient last visit scan (LPLV) plus 24 M. The Sponsor, CI and/or the TSC have the right at any time to terminate the study for clinical or administrative reasons.
End of follow-up.

The end of the study will be reported to the Sponsor, REC and NHS R&D Offices within 90 days, or 15 days if the study is terminated prematurely. The CI will ensure that any appropriate follow up is arranged for all participants.

A summary report of the study will be provided to the Sponsor and REC within 1year of the end of the study.

10.4. CONTINUATION OF TREATMENT FOLLOWING THE END OF STUDY

All participants will enter the standard NHS care pathway after their last scan; for further investigations or treatment if: a positive scan, classified nodules or incidental finding or if a non-referable scan is determined they will be monitored by their GP if they become symptomatic for lung cancer.

11. REPORTING, PUBLICATIONS AND NOTIFICATION OF RESULTS

11.1. AUTHORSHIP POLICY

Ownership of the data arising from this study resides with the study team. On completion of the study, the study data will be analysed and tabulated, and a clinical study report will be prepared. Authorship eligibility for each manuscript arising from this study will be determined according to the criteria laid out in the Working Practice Document on Authorship filed in the Study Operations Manual.

11.2. PUBLICATION

The clinical study report will be used for publication and presentation at scientific meetings. Trial Investigators have the right to publish orally or in writing the results of the study.
Summaries of results will also be made available to trial Investigators for dissemination within their clinical areas (where appropriate and according to their discretion).

11.3. PEER REVIEW

This trial has undergone peer review by the Sponsorship Committee. The trial design and results will be reviewed in publications by the referees of the journal to which the paper (and its protocol) will be submitted.

12. REFERENCES/BIBLIOGRAPHY

1. Aberle D, Adams A, Berg C, Black W, Clapp J, et al. Reduced Lung-Cancer Mortality with Low-Dose Computed Tomographic Screening. National Lung Screening Trial Research Team, New England Journal of Medicine 2011;365(5):395-409.
2. Bach, PB; Mirkin, JN; Oliver, TK; Azzoli, CG; Berry, DA et al Benefits and Harms of CT Screening for Lung Cancer. A Systematic Review. JAMA 307 22 2418-2429 DOI: 10.1001/jama.2012.5521
3. Bower P, Wilson S, Mathers N. How often do UK primary care trials face recruitment delays? Family Practice 2007; 24: 601–603
4. Boyle P, C. J. Chapman, S. Holdenrieder, A. Murray, C. Robertson, W. C. Wood Benefits and Harms of CT Screening for Lung Cancer. JAMA. 2012 May20, 1.2.
5. Boyle P, Chapman CJ, Holdenrieder S, Murray A, Robertson C, Wood WC, et al. Clinical validation of an autoantibody test for lung cancer. Annals of Oncology 2011;22(2):383-89.
6. Campbell MK, Snowdon C, Francis D, Elbourne D, McDonald AM, Knight R, Entwistle V, Garcia J, Roberts I, Grant A. Recruitment to randomised trials: strategies for trial enrolment and participation study. The STEPS study. Health Technology Assessment 2007; Vol. 11: No. 48
7. Chapman C, Robertson J, Murray A, Titulaer M, Lang B, Thorpe A, et al. The Presence of Autoantibodies to Tumour-Associated Antigens Can Predate Clinical Diagnosis of Small Cell Lung Cancer Chest 2010;138(No. 4 Supplement):775A.
8. Chapman CJ, et al. EarlyCDT®-Lung test: improved clinical utility through additional autoantibody assays. *Tumor Biology* 2012; DOI: 10.1007/s13277-012-0379-2.
9. Chou R, LeFevre ML. Prostate cancer screening--the evidence, the recommendations, and the clinical implications. JAMA. 2011 Dec 28;306(24):2721-2. doi: 10.1001/jama.2011.1891.
10. Edwards PJ, Roberts I, Clarke MJ, DiGuiseppi C, Wentz R, Kwan I, Cooper R, Felix LM, Pratap S. Methods to increase response to postal and electronic questionnaires. Cochrane Database of Systematic Reviews 2009, Issue 3. Art. No.: MR000008. DOI: 10.1002/14651858.MR000008.pub4.

11. Finn OJ. Immune Response as a Biomarker for Cancer Detection and a Lot More. New England Journal of Medicine 2005;353(12):1288-90.
12. Hardcastle J, Chamberlain J, Robinson M, Moss S, Amar S, Balfour T, et al. Randomised controlled trial of faecal-occult-blood screening for colorectal cancer. Lancet 1996;348:1472-7.
13. Hewitson P, Glasziou PP, Irwig L, Towler B, Watson E. Screening for colorectal cancer using the faecal occult blood test, Hemoccult. Cochrane Database of Systematic Reviews, 2007 Issue 1.
14. Lam S, Boyle P, Healey GF, Maddison P, Peek L, Murray A, et al. EarlyCDT-Lung: An Immunobiomarker Test as an Aid to Early Detection of Lung Cancer. Cancer Prevention Research 2011;4(7):1126-34.
15. Macdonald IK, Allen J, Murray A, Parsy-Kowalska CB, Healey GF, et al. (2012) Development and Validation of a High Throughput System for Discovery of Antigens for Autoantibody Detection. PLoS ONE 7(7): e40759. doi:10.1371/journal.pone.0040759
16. MacMahon H, Austin JHM, Gamsu G, Herold CJ, Jett JR, Naidich DP, et al. Guidelines for Management of Small Pulmonary Nodules Detected on CT Scans: A Statement from the Fleischner Society1. Radiology 2005;237(2):395-400.
17. Mathew J, Healey G, Jewell W, Murray A, Chapman C, Peek L, et al. Demographics of populations at high risk of lung cancer and results of the Early CDT-Lung test. Journal of Clinical Oncology 2010;28(no. 15_suppl):7033.
18. Mayor S. Critics attack the new NHS breast screening leaflets for failing to address harms. BMJ2010;341:c7267.
19. McCartney M. Doctors should stop supporting unethical screening. BMJ 2011;343;d4592.
20. Mirkin JN. Benefits and Harms of CT Screening for Lung Cancer. A Systematic Review
21. Murray A, Chapman CJ, Healey G, Peek LJ, Parsons G, Baldwin D, et al. Technical validation of an autoantibody test for lung cancer. Annals of Oncology 2010;21(8):1687-93.

22. Oken, M.M., Creech, R.H., Tormey, D.C., Horton, J., Davis, T.E., McFadden, E.T., Carbone, P.P.: ECOG Performance Status ref Toxicity And Response Criteria Of The Eastern Cooperative Oncology Group. Am J Clin Oncol 5:649-655, 1982.)

23. Robertson J, Chapman C, Cheung K, Murray A, Pinder S, Price M, et al. Autoantibodies in early breast cancer. Journal Clinical Oncology 2005;23 No. 16S, Part I of II (June 1 Supplement)(2005 ASCO Annual Meeting Proceedings):549.

24. Scottish Primary Care Research Network. Scottish School of Primary Care - SPCRN. http://www.sspc.ac.uk/spcrn/: Last accessed 10.1.12.

25. Spitz MR, et al. A risk model for prediction of lung cancer. *J Natl Cancer Inst* 2007; 99(9):715-726.

26. Swensen SJ, Jett JR, Hartman TE, Midthun DE, Mandrekar SJ, Hillman SL, et al. CT Screening for Lung Cancer: Five-year Prospective Experience1. Radiology 2005;235(1):259-65.

27. Tan EM. Autoantibodies as reporters identifying aberrant cellular mechanisms in tumorigenesis. The Journal of Clinical Investigation 2001;108(10):1411-15.

28. Treweek S, Pearson E, Smith N, Neville R, Sargeant P, Boswell B, et al. Desktop software to identify patients eligible for recruitment into a clinical trial: using SARMA to recruit to the ROAD feasibility trial. Informatics in Primary Care 2010;18(1):51-58.

29. Treweek S, Pitkethly M, Cook J, Kjeldstrøm M, Taskila T, Johansen M, Sullivan F, Wilson S, Jackson C, Jones R, Mitchell E. Strategies to improve recruitment to randomised controlled trials. Cochrane Database of Systematic Reviews 2010, Issue 4. Art. No.: MR000013. DOI: 10.1002/14651858.MR000013.pub5.

30. US Preventive Services Task Force. Screening for breast cancer: recommendation statement. Ann Int Med2009;151:716-26

31. Vedhara K, Kendrick D. Early Cancer Detection Test: Maximising Recruitment. University of Nottingham Report August 2012

32. Zhong L, Coe SP, Stromberg AJ, Khattar NH, Jett JR, Hirschowitz EA. Profiling Tumor-Associated Antibodies for Early Detection of Non-small Cell Lung Cancer. Journal of Thoracic Oncology 2006;1(6):513-19.

33. Edwards PJ, Roberts I, Clarke MJ, Diguiseppi C, Wentz R, Kwan I, et al. Methods to increase response to postal and electronic questionnaires. Cochrane Database Syst Rev 2009(3)

34. British Journal of Cancer (2013), 1–5 | doi: 10.1038/bjc.2013.1

APPENDIX 1: PREPARATORY FOCUS GROUP WORK

1.1 Protocol

Research Protocol

Maximising recruitment in early cancer detection trials: The lung cancer trial

Version 1: 30/4/12

Investigators:

Professor Kavita Vedhara (IWHO, University of Nottingham)

Professor Denise Kendrick (Primary Care, University of Nottingham)

Professor John Robertson (Division of Breast Surgery, University of Nottingham)

Dr Roshan Das Nair (NUH NHS Trust & University of Nottingham)

Dr Kate Skellington-Orr (KSO Research Limited, Glasgow)

Professor Frank Sullivan (Population Health Sciences, University of Dundee)

Background

Approximately two thirds of trials fail to reach their recruitment target or have to extend their recruitment period (1,2). Failing to fulfil recruitment targets rates leads to underpowered studies, reduced generalisability, increased costs and delays in the implementation of effective interventions. Maximising recruitment is thus key to a trial's success.

Funding has been obtained for a large trial of the effectiveness and cost effectiveness of early cancer detection test in lung cancer, which is to be evaluated in individuals at high risk of lung cancer. The trial will be undertaken in general practices from disadvantaged areas in Glasgow and Dundee, commencing October 2012. Recruitment to trials amongst disadvantaged populations can be particularly challenging due to a lack of trust, limited knowledge of research and low literacy amongst potential participants (3). Previous research has demonstrated that qualitative methods

can be used to inform recruitment strategies by tailoring recruitment to the trial population. For example, the ProtecT feasibility study, for prostate specific antigen testing for prostate cancer, explored men's views of trial participation, interpretation of study information, understanding and acceptance of randomisation and treatment. Findings fed into recruitment strategies and this resulted in the proportion of men consenting to randomisation increasing from 49% to 70% (4).

The current research has been designed to deliver tailored recruitment strategies and materials for the population to be targeted in the forthcoming early lung cancer detection trial. We propose to achieve this be addressing the following aims:

Aims

1. To explore potential trial participant views on:
    (a) issues likely to influence recruitment into the trial and willingness to be randomised (e.g., recruitment strategies; understanding of risk information; clinical equipoise and randomisation)
    (b) recruitment and study documentation (e.g., invitation letter, questionnaires);
    (c) factors which facilitate and hinder trial participation.
2. To develop recruitment processes and materials for use in this, and subsequent trials.
3. To contribute to the literature on methods for enhancing trial recruitment

Methods

We will be working with a local research company based in Scotland (KSO Research Limited) who will identify eligible participants; undertake the focus groups and complete the transcribing and analysis of material obtained through focus group discussions. As the clinical trial will recruit patients from both Glasgow and Dundee, we will be seeking to conduct 2 focus groups in each city, with up to 10 participants in each group.

Participants: The population to be targeted in the trial will be individuals at high risk of developing lung cancer aged between 50-75 years i.e., individuals who self-identify as current or former cigarette smokers with at least 20 pack-years, or a history of cigarette smoking plus family history of lung cancer which gives an individual a personal risk similar to a smoking history of 20 pack years).

Recruitment: KSO Research limited will use 'on-street' recruitment methods to recruit participants into the focus groups. This will involve a trained recruiter working in each of the two areas. The recruiter will target local amenities where eligible participants may be found, for example, smoking areas outside of recreational facilities, at local train or bus stations, etc. Recruitment will take place at different times/days over one week. The company have used this kind of 'on-street' recruitment before with considerable success. They have observed that finding people who agree 'at random' to participate are often more likely to show genuine commitment to participation than those who respond to press advertisements. Furthermore, meeting the recruiter face-to-face encourages an early relationship and opportunity for participants to ask any questions that they have about participation on the spot before agreeing to take part. This approach also has the advantage of being cheaper than a press advertisement with no risk of over-subscription. In accordance with usual practice, the local police will be notified of the recruiters' on-street presence and will be done with their support.

Individuals will be approached on the street at random, although efforts will be made to recruit a mix of genders in each area and to fill quotas in three separate age bands (50-59, 60-69, 70-75 years). The recruiter will first introduce themselves; provide a brief verbal introduction to the research and will enquire whether the individual would be willing to discuss a research project about a new blood test for lung cancer involving people who currently smoke or who have smoked previously (see recruitment questionnaire). Those who consent to further conversation will be asked first about their smoking status and family history of lung cancer to establish eligibility. Those who

are not eligible will be thanked for their time and will only be given further information about the research should this be requested. Those who are eligible will be asked additional brief screening questions regarding age and working status. Individuals who agree to participate immediately will receive a participant cover letter and information sheet (enclosed) and will be asked to provide contact information so that they can be re-contacted and reminded of the date, time and location of the focus group. Participants who wish to consider the invitation first, will be provided with contact details for the research team and a participant information sheet. Individuals will be advised that they will receive £30 cash to thank them for their time and to cover their out of pocket and travel expenses. Anonymised data will be provided to the research team on:

1. The number of recruitment sessions undertaken
2. The number of people approached
3. The number of those approached who were eligible to participate and reasons for ineligibility
4. The number of those eligible who agreed to take par
5. The reasons why people chose not to take part
6. Characteristics of those who were eligible who did and did not agree to take part and the reasons for non participation (in an EXCEL spreadsheet)
7. The number of those agreeing to take part who attended each focus group

Procedure: Written informed consent (enclosed) will be obtained from all participants prior to commencing the focus groups. Participants will be reminded that the discussions of the group are to be audio-taped and transcribed verbatim, but that they will not be identified in the recordings and, as such, their contributions to the discussions will remain anonymous. Participants will be asked to discuss issues related to the aims outlined in 1a-c above, with discussions structured according to a topic guide (enclosed). Participants will receive £30 at the end of the focus group to thank them for their time and to cover out of pocket and travel expenses and will be asked to sign a receipt for this.

Analysis: Audio recordings of focus groups will be transcribed verbatim and a thematic analysis will be undertaken to provide a rich and detailed account of the data (5). Strategies for ensuring quality assurance of credibility, transferability, dependability, and confirmability of analysis will be followed (6). The information from this analysis will be used to further refine the recruitment strategy and materials for the main trial. All original data files will be confidentially destroyed and written data stored anonymously.

References:

(**1**) Watson JM, Torgerson DJ. Increasing recruitment to randomised controlled trials: a review of randomised controlled trials. *BMC Med Res Method* 2006, *6*:34 doi:10.1186/1471-2288-6-34.

(**2**) McDonald AM, et al. What influences recruitment to randomised controlled trials? A review of trials funded by two UK funding agencies. *Trials* 2006, *7*:9 doi:10.1186/1745-6215-7-9.

(**3**) Lovato LC, et al. Recruitment for controlled clinical trials: literature summary and annotated bibliography. *Control Clin Trials* 1997;18(4):328-52.

(**4**) Donovan J, et al. Prostate Testing for Cancer and Treatment (ProtecT) feasibility study. *Health Technol Assess* 2003;7(14):1-88.

(**5**) Braun V, Clarke V. Using thematic analysis in psychology. *Qualitative Research in Psychology* 2006 3: 77-101.

(**6**) Shenton A. Strategies for ensuring trustworthiness in qualitative research projects. *Education for Information* 2004; 22: 63-75.

1.2 APPROVAL

Institute of Work, Health & Organisations
http://www.nottingham.ac.uk/iwho

The University of Nottingham
UNITED KINGDOM · CHINA · MALAYSIA

Kavita Vedhara

Faculty of Medicine & Health Sciences
School of Community Health Sciences
Institute of Work, Health & Organisations
The University of Nottingham
International House
Jubilee Campus
Nottingham
NG8 1BB
t: +44 (0)115 9515315
f: +44 (0)115 846 6625

25/05/2012

Dear Kavita

I-WHO Ethics Committee Review

Thank you for submitting your proposal on "Maximising recruitment in early cancer detection trials: The lung cancer trial". This proposal has now been reviewed by I-WHO's Ethics Committee to the extent that it is described in your submission.

I am happy to tell you that the Committee has found no problems with your proposal. If there are any significant changes or developments in the methods, treatment of data or debriefing of participants, then you are obliged to seek further ethical approval for these changes.

We would remind all researchers of their ethical responsibilities to research participants. The Codes of Practice setting out these responsibilities have been published by the British Psychological Society. If you have any concerns whatsoever during the conduct of your research then you should consult those Codes of Practice and contact the Ethics Committee.

You should also take note of issues relating to safety. Some information can be found in the Safety Office pages of the University web site. Particularly relevant may be:
The *Safety Handbook*, which deal with working away from the University.
http://www.nottingham.ac.uk/safety/
Safety circulars: Fieldwork P5/99A on http://www.nottingham.ac.uk/safety/fieldwork.htm
Overseas travel/work P4/97A on http://www.nottingham.ac.uk/safety/overseas.htm
Risk assessment on http://www.nottingham.ac.uk/safety/risk-assessment.htm

Responsibility for compliance with the University Data Protection Policy and Guidance lies with all researchers.

Ethics Committee approval does not alter, replace or remove those responsibilities, nor does it certify that they have been met.

We would remind all researchers of their responsibilities:

- to provide feedback to participants and participant organisations whenever appropriate, and
- to publish research for which ethical approval is given in appropriate academic and professional journals.

Yours sincerely

pp Neil Coulson

Professor Nadina Lincoln
Chair IWHO Ethics Committee

1.3 FINAL REPORT

SEPARATE DOCUMENT (attached)

APPENDIX 2: ECLS STUDY: QUALITATIVE SUB-STUDIES

(Version 1, 11-09-13)

Study background information and rationale

Three qualitative sub-studies have been developed to explore a number of aspects of participant s' attitudes and experiences of EarlyCDT-Lung testing and changes in smoking behaviour:

Sub-study one: Investigating the experiences of individuals who choose not to have the EarlyCDT-Lung test (Ben Young)

In order to maximise the effectiveness of a screening test in a population, uptake must be high. Those at highest risk of a disease are often least likely to attend for screening[1] and rates of uptake can vary according to the type of screening[2, 3]. Furthermore, lung cancer screening trial participants have previously displayed gaps in essential knowledge, suggesting that the goal of informed choice in lung cancer screening may be difficult to achieve[4]. Quantitative research suggests screening uptake may be related to demographic factors, health status and attendance at previous screening tests[3, 5, 6]. The acceptability of a screening method is also a recurring factor in decisions to attend[2].

Lung cancer screening differs from other cancer screening because it targets a higher risk subgroup of the population characterised predominantly by smoking status. Barriers to uptake of lung cancer screening may include the absence of symptoms, lack of knowledge about the test and stigma associated with lung cancer[4, 7, 8]. Smokers may be more likely to perceive early detection and intervention to be of limited use[9]. It is important to develop an in depth understanding of the reasons some people decide not to have the EarlyCDT-Lung test in order to promote future uptake in those most at risk of lung cancer.

Objectives:

1. To explore decisions to not respond to ECLS Study invitations, reasons for not responding and the perceived barriers to attending for the ECLS Study visit.
2. To assess non-responders' understanding and knowledge of the information communicated to them about the EarlyCDT-Lung test.
3. To identify theory-based methods which could increase recruitment in a future trial or screening programme for early lung cancer detection.

Sub-study two: How do participant s perceive the EarlyCDT-lung test and what do they understand about their test results? (Laura Bedford & Gozde Ozakinci)

As the EarlyCDT-lung test is a different type of screening test for lung cancer it is important to examine participant s' beliefs and expectations about the test. Qualitative research has highlighted different aspects of screening tests that individuals can hold beliefs about, for example, beliefs about the accuracy (sensitivity) of the test, the nature of the test, risks and side effects associated with the test, and type of result obtained using the test. Together, each of these beliefs can make up an individual's overall perception of a screening test. Relatively limited research to date has found that screening test beliefs are one of several factors that can influence screening uptake[8] and predict emotional and behavioural responses to screening test results[10-12]. As there is currently no method to quantitatively assess screening test beliefs, qualitative work in this area will be valuable as it will inform the development of a measure to capture participant s' beliefs about screening tests. Furthermore, findings will inform how information on the EarlyCDT-lung test is presented to participant s.

A participant's understanding of their test result also has the potential to influence psychological outcomes following screening. For example, research has shown that a lack of understanding of the correct meaning of a positive screening test result can predict emotional distress following receipt of

screening test results[13, 14]. Misunderstanding of a negative or normal screening test result has been found to lead to false reassurance, where an individual incorrectly understands a negative screening test result to mean that they are at no risk of developing the condition being screened for[15, 16]. In addition to understanding of test results, dissatisfaction with information provided on test results has also been found to predict adverse emotional outcomes, such as high levels of anxiety and worry[15-17].

It is therefore important to explore what participant s understand about their EarlyCDT-lung test result and identify what information they need about their result as this will help to inform future communication of EarlyCDT-lung test results. Furthermore, the majority of work exploring understanding of screening test results and satisfaction with test result information has used quantitative methodology, so it would be valuable to conduct qualitative research in this area so that these factors can be explored in more depth.

Objectives:

1. To examine participants' understanding of, and satisfaction with, the information they received about the test
2. To explore participants' beliefs about the EarlyCDT-lung test
3. To examine participants' understanding of their test result
4. To find out how satisfied participants were with the information provided on their test result.

<u>Sub-study three - An examination of changes in smoking behaviour following EarlyCDT-Lung testing (Ben Young)</u>

Smokers who attend lung cancer screening may be more motivated to stop smoking than other smokers[18-20] and they may experience a 'teachable moment' for smoking cessation, a brief period in which motivation to stop smoking is enhanced[21]. However, evidence of changes in smoking behaviour

in lung cancer screening participant s is inconclusive and long term changes in smoking prevalence in screened groups have not generally been observed[22, 23]. Increased cessation rates have been observed in participants receiving abnormal results[21, 24]. The ECLS Study will measure smoking behaviour and attitudes to smoking in a sample of participants over 12 months and this sub-study aims to provide an in depth exploration of those individuals' experiences in relation to smoking during the study.

Perceived barriers to smoking cessation can include current smoking behaviour, motivation to quit, past quit attempts, preferences for cessation support and fear of withdrawal symptoms, or of being judged or failing[25, 26]. Facilitators to cessation can include concerns about health, cost and the views of others[27], as well as support services which are perceived to be personalised, accessible and effective[25]. The implementation of smoking cessation interventions as part of lung cancer screening programmes is being advocated[28], creating a need for an evidence based approach to the integration of cessation interventions in such programmes. An exploration of the perceived barriers and facilitators to smoking behaviour change in ECLS Study participants can inform the development of relevant and acceptable cessation support in the lung cancer screening context.

Objectives:

8. To identify and explore decisions made regarding smoking cessation, reasons for those decisions and perceived barriers and facilitators to smoking cessation in screened ECLS Study participants.
9. To compare differences in thoughts and experiences regarding smoking cessation between:
    a. Those who are successful in stopping smoking (i.e. reporting a change in smoking status from smoker to non-smoker), those who are unsuccessful (i.e. reporting a cessation attempt but no change in smoking status) and those who do not attempt to stop smoking (i.e. reporting no cessation attempt).
    b. Those who receive a positive lung cancer screening test result and those who receive a negative result.

<u>Method</u>

1. Study management

The studies will be conducted by Laura Bedford and Ben Young (PhD students) under the supervision of academic supervisors Kavita Vedhara (Professor of Health Psychology), Denise Kendrick (Professor of Primary Care Research) and John Robertson (Professor of Surgery), three ECLS study Principal Investigators based at the University of Nottingham, a research partner. In addition, Roshan das Nair (Consultant Clinical Psychologist and Honorary Associate Professor) will assist. Dr Gozde Ozakinci (University of St Andrews) will conduct analysis of Sub-study 2 data.

2. Duration

Sub-studies will take place between September 2013 – September 2015.

3. Selection of participants

<u>Sub-study one – Investigating the experiences of individuals who choose not to have the EarlyCDT-Lung test (Ben Young)</u>

One recruitment strategy for the main ECLS study is identifying potential participants via SPCRN from primary care. Each GP list is screened by the GP to ensure eligibility and suitability. Invitation letters are sent via the Health Informatics Centre. These individual's details are not know to the study team. Individuals who respond positively either by returning the reply slip or calling/emailing the study team directly will be registered on the Recruitment Tracker. The recruitment tracker is held within the HIC safehaven and ensures participant confidentiality and data security as per HIC information governance Standard Operating Procedures. The contact details of these individuals are released by HIC to the study team to allow further contact. Invited individuals who respond indicating they would like no further contact are registered on the recruitment tracker as a negative response. The specific details of these individuals are not visible to the study team, merely documented as a number of negative

responses. The group which responded neither positively nor negatively are termed non-responders. This group can often be over 80% of the invited population. As per the study protocol these individuals will be sent a reminder letter/postcard.

Participants unable to speak English will not be eligible for any of the three sub-studies as they involve with telephone or face to face interview. A surrogate marker for sub-studies 2 & 3 will be eligibility into the main ECLS study. For sub-study 1, eligibility will be assessed at the beginning of the telephone interview.

For sub-study 1, eligible participants will be those within the non-responder group and who would have been eligible for the main ELCS study had they responded and can confirm they remember receiving the invitation and that they intentionally did not respond.

Non-responders will be identified periodically and sampled purposively from both the Glasgow and Dundee areas as recruitment to ECLS Study progresses. Consent will be obtained to participant in sub-study 1.

Participants in the main ECLS study are invited to give consent to be contact for further contact. Only those participants who have given permission will be contacted with regard to sub-studies 2 & 3. Participants who have withdrawn from the main study or have received a diagnosis of lung cancer will not be eligible for sub-studies 2 &3.

For all sub-studies one reminder will be sent. If the invited participant does not reply to either the initial invitation or the reminder they will not be contacted again for the sub-study participation.

Sub-study 2: How do participants perceive the EarlyCDT-lung test and what do they understand about their test results? (Laura Bedford and Gozde Ozakinci)

Eligible participants will be:

- Willing and able to give informed consent for participation in the study
- In the EarlyCDT-negative group or the EarlyCDT-positive group
- Have received their screening test result within the past eight weeks.

The aim is to recruit a diverse sample. This can be achieved through a maximum variation sampling strategy, which is recommended for qualitative studies.[29] A sample obtained using maximum variation is not a representative sample but a purposive sample recruited to tap a variety of different views on a subject. This requires a strategy for sampling people who are different on a wide range of demographics. In this study, age, gender, ethnicity, level of deprivation (from the Scottish Index of Multiple Deprivation) and education level will be considered as variation factors. Roughly equal numbers of participants from Glasgow and Dundee will be recruited. Maximum variation sampling is an iterative process, whereby the first few participants sampled will direct who is sampled next.[30]

It is expected that the final sample will consist of 30 participants, 15 from the EarlyCDT-positive group and 15 from the EarlyCDT-negative group. It is expected that saturation will be achieved with this sample; however, up to two participants from each group may be further recruited if saturation is not achieved. Both groups of participants will be recruited at the same rate. The sampling of participants will be dictated by the overall number required for this study. As previously stated, it is anticipated that 15 participants will be sampled per group over the 10-month study period. Therefore, approximately six participants will be approached per month per group with the aim of recruiting two participants from each group (33% response rate). A review will be carried out after two months of recruitment, and if response rates are low, then the number of participants approached each month will be increased. The order of selecting participants will be based on maximum variation as described above.

Sub-study 3 - An examination of changes in smoking behaviour following EarlyCDT-Lung testing (Ben Young)

Eligible participants will have been: i) allocated to the ECLS Study screened group; ii) selected for follow up questionnaires; iii) have been a self-reported smoker at baseline. Participants reporting either successful or unsuccessful cessation attempts, or no attempt, will be sampled from groups receiving a positive test result and a negative test result, making six distinct groups for this sub-study.

- A smoker at baseline will be defined as a 'YES' response to the question: "Have you smoked any cigarettes or tobacco in the last seven days/week?"
- A cessation attempt will be identified as a 'YES' response to the question at 3 months: "In the LAST 2 MONTHS have you tried to stop smoking?"
- A successful cessation attempt will be defined as a smoker at baseline and a 'NO' response to the following question at 3 months: "Have you smoked any cigarettes or tobacco in the last seven days/week?"

Individuals will be periodically sampled from participants in the RCT and approached in advance of planned researcher visits to Scotland. They will be sampled in a stratified quota manner with the objective of 15 negative test and 15 positive test participants and the aim of a 5-5-5 smoking behaviour split in each group. It is anticipated that 150 individuals will be approached with a recruitment rate of 20%, however the number of people sampled will be adjusted depending on response rates, the number of participants becoming eligible in each group and the available time in the relevant researcher visit to Scotland.

4. Participant recruitment

Sub-study 1

Eligible participants will be mailed an invitation letter, information sheet and consent and contact forms requesting a contact telephone number and convenient times to call, to be returned in a prepaid envelope. This process will be carried out via HIC as the contact details of ECLS study non-responders are not known to the study team. The replies will be returned to HIC and any positive responses will be uploaded onto the recruitment tracker. The researcher's telephone number and ECLS study team details will be provided for any queries. If a participant's telephone number is available, a follow up call will be made by the researcher to confirm receipt of the mailing and answer any questions. If there is no answer, a voicemail message may be left or one further call attempt made at a different time.

On receipt of a completed consent form and contact form participants will be telephoned by the researcher at a time they have indicated as being convenient. Verbal consent will be confirmed at the beginning of the call. Eligibility for the study will be confirmed and any questions from the participant will be answered. Should an individual indicate that they now wish to take part in the ELCS Study following contact regarding the sub-study, they will become ineligible for the sub-study and they will instead be telephoned by the ECLS Study research team as described in the main protocol.

Sub-studies 2 & 3

The researcher will send potential participants an invitation letter and a participant information sheet. The invitation letter will include a seven-day response deadline to ensure that the interview will take place as soon as possible of receipt of test result. Potential participants will be invited to indicate their interest in the research by completing a short form and posting it back to the researcher in the freepost envelope provided, or contacting the researcher by e-mail or phone. Once a response is received from a participant, the researcher will contact the participant by phone to arrange a date and time for their interview. During this phone call, the researcher will explain the details of the study and

answer any questions that the participant has concerning study participation. An appointment letter or e-mail will be sent to the participant to confirm the date and time of their interview. The day before the interview, the participant will be contacted by phone, text, or e-mail as a reminder. If the participant is unable to attend the interview, but would still like to take part, then another interview will be arranged.

[Please note: in order to maximise comprehension of study materials, readability of all participant documents has been tested using a readability programme. The participant information sheet layout and format was informed by the results of a study that applied the user testing method to improve the readability of a participant information sheet[31]. The participant information sheet (PIS) for study two was also piloted with three participants who matched those who will be eligible for recruitment to the trial.]

5. Procedure

5.1. Obtaining informed consent

Sub-study 1. Potential participants will be sent PIS and informed consent form (ICF) with their letter of invitation. Contact numbers are given to contact the study team to answer any questions they may have. They are invited to send their reply and completed consent form in the prepaid envelope (to HIC). A copy of the ICF be kept by the participant and the original filed the study ISF. Prior to the telephone interview they will be asked to confirm their verbal consent. One consent form will be kept by the participant and the other kept by the researcher and filed in the ISF.

Sub-studies 2&3. At the start of each face-to face interview, participants will be provided with a copy of the PIS and informed consent form. They will be asked if they have any further questions concerning their involvement in the study and asked to sign the ICF. If the interview is to be conducted by

telephone, the ICF will have been sent in advance of the interview and the participant will be asked to return to the study team using the prepaid envelope. Prior to conducting the telephone interview verbal consent will be confirmed. The process for obtaining consent will be in accordance with the REC guidance and Good Clinical Practice (GCP). Two consent forms will be signed and dated by the participant and the researcher. A copy of the ICF be kept by the participant and the original filed the study ISF.

5.2. Interview procedure

It is estimated that each interview will take no more than an hour. All interview questions have been structured around the aims of each sub-study (see interview schedules below).

Sub-study one

Participants will take part in a semi-structured interview by telephone which will be digitally recorded. There is a precedent for using this method for the study of non-responders to cancer screening trials in previous research in the UK[8, 32, 33]. It is anticipated telephone interviews will be more acceptable and convenient than face-to-face interviews in a population of non-responders and this method has been used successfully in similar studies[8, 32]. An initial interview schedule can be found in the appendices and these questions will be developed further by the findings of additional pilot work and a qualitative meta-synthesis currently being undertaken. Approximately 20 interviews will be conducted, however recruitment will continue until no new themes emerge from the interviews.

Sub-studies two and three

The participant will be interviewed in a one-to-one semi-structured interview at their local facility (CRC/CRF, GP practice) or if required their home. They can also take part in the interview over the phone. The interview will be conducted in a location where it cannot be overheard. Only the researcher and the participant will be present at the interview, however, the participant will have the

option to have someone with them if they would prefer. If the participant would prefer a home visit, where possible a study nurse will also attend but will not present at the interview unless invited by the participant. All interviews will be carried out by the researcher who will adhere to local policies of research fieldwork. Participants will be offered taxi transport or travel expenses to attend face-to-face interviews.

5.3. Incentives

Participants in all three sub-studies will be given a £5 gift voucher for use in a range of stores to thank them for their time. If they request it, participants will receive a lay summary of the findings of the study when it is complete.

5.4. Withdrawal from each sub-study

Participants may be withdrawn from each study either at their own request or at the discretion of the researcher. The participants will be made aware that they do not have to give a reason for withdrawing from the study and withdrawing will not affect their future care. Participants will be asked if any information collected prior to withdrawal be kept for future analysis.

6. Analysis

Interviews for all three sub-studies will be audio recorded, transcribed verbatim and analysed using thematic analysis. The process of thematic analysis will be informed by the following phases outlined by Braun and Clarke[34] in their step-by-step guide to doing thematic analysis:

1) Familiarising oneself with the data (i.e., transcribing and re-reading the data),

2) Generation of codes (i.e., developing codes that identify key features of the data. This will be done using NVivo software),

3) Searching for themes (i.e., sorting the codes into themes and gathering all the data relevant to each theme. Consideration will be given to how codes can be combined to form an overall theme)

4) Reviewing of themes (i.e., checking the extracts for each theme, ensuring that they form a clear pattern, and developing a thematic map), and

5) Defining and naming themes.

An initial analysis of the data will be conducted by both researchers [LB & BY]. They will discuss the results of each analysis and a final thematic framework will agreed upon. A third researcher [RdN] will review the framework and results of both analyses. This procedure will enable a validation of the themes and provide an in-depth interpretation of the data. The 18 and 24 month psychological data will be analysed by the forth researcher [MC].

7. Adverse events

The occurrence of adverse events as a result of participation within these studies is not expected. However, the researchers are aware that certain questions will be of a sensitive nature, which participants might find distressing. Participants will be made aware that they do not have to answer all of the questions if they do not want to. The researchers will remind participants that they can withdraw from the study at any time without giving a reason. In the unlikely event the participant feels distressed by the interview; they will be signposted to local services for further support if required and to the ECLS study research team.

8. Records and record retention

All data will be stored in compliance with the Data Protection Act 1998 and in accordance with University of Dundee, Health Informatics Standard operating Procedures and the Universities of Nottingham and St Andrews Code of Research Conduct and Research Ethics (non-identifiable study management information only, dates of interviews etc). The researchers [LB, BY, MC & GO] will be responsible for maintaining all documents concerning the study. The database will be stored on a University of Nottingham password protected computer in a locked office at Queens Medical Centre

and will only be accessible by the research team and a University of St Andrews password protected computer in a locked office (e.g., the researchers and research supervisors).

Each participant will be assigned a unique study identity code number for use on their interview transcript. Transcripts and signed consent forms will be treated as confidential documents and held securely in accordance with regulations. Each transcript document will be password protected and will be stored on a password protected file on University of Nottingham server. Only members of the research team (e.g., the researchers and the research supervisors) will have access to interview transcripts. Audio recordings will be stored on an audio recorder in a locked filing cabinet. Once audio recordings have been transcribed, the recording will be deleted off the audio recorder. Signed consent forms will be kept in the study ISF in a locked filing cabinet in a locked office at Queens Medical Centre. In line with the University of Nottingham Code of Research Conduct and Research Ethics, data will be stored for seven years from the date of any publication that is based upon them.

9. Data protection

All members of the research team will adhere to the Data Protection Act, 1998. Study documents will contain only the minimum required information for the purposes of the study.

10. Publication and Dissemination

The results of the sub-studies will be used for publications in peer reviewed scientific journals, conference presentations and a PhD thesis. Participants will not be identified in any publications.

11. Funding source

The sub-studies are funded by Oncimmune Ltd, University of Nottingham and the Dundee Cancer Centre.

Sub-study 1: Telephone Interview Schedule

Please note: This is an interview guide. The questions posed will relate to this guide, but the exact questions will be formulated based on the individual's responses to previous questions during the interview and on the basis of the preceding interviews with other participants. This iterative process is required when using qualitative methods to explore themes fully.

It will have been established in the pre-interview screening call that the participant remembers receiving the ECLS Study invitation mailing and made a conscious decision to not respond to it.
Preliminary data gathering tool

At the start of the interview the researcher will ask the following structured questions as a preliminary data gathering tool.

1. What is your age?
2. Are you married/single/cohabiting?
3. What is your current work situation?
4. How would you describe your current health? Prompts:
   - Do you have any problems getting around?
   - Are you taking any medication?
5. Have you ever been tested for any diseases, called a 'screening test'?
   - For lung cancer?
   - For other cancers?
   - For other diseases?
   - If yes, why did you have the test(s)?
   - If no, why not?
6. Are you a smoker?

- If yes, how many cigarettes do you smoke per day on average and for how many years have you smoked?
- If no, have you ever smoked?

7. Do you have any family history of lung cancer? Or other cancers?
   - If yes, prompt to elaborate.

Topic Guide for semi-structured interview

Aim: To find out how the participant reacted to receiving the ECLS Study invitation letter/reminder

1. When people receive a letter like this they often have many different thoughts and feelings. Please describe your thoughts and feelings when you received the mailing? When you received the invitation mailing for the Early Lung Cancer Detection Study, please describe how you felt about it?

Prompts:

a. Thoughts and feelings might be about the mailing itself, the screening test, the research study, lung cancer, your health in general or other information you read in the mailing

b. They might be positive or negative thoughts or feelings

c. They might be prolonged or brief thoughts or feelings

d. How did you feel when you received the mailing? Why did you feel like that?

e. What thoughts did you have when you read the letter? How did those thoughts make you feel?

f. How did you feel 5 minutes after reading the letter and leaflet? Were you still thinking about it? If so, what were you thinking?

g. How did you feel 24 hours after reading the letter and leaflet? Were you still thinking about it? If so, what were you thinking?

h. How much of the letter did you read?

i. How much of the leaflet did you read?

j. Where did you put them?

k. Did you show them to anyone else?

i. What did they say about them?

ii. How did this influence you?

l. Did you discuss them with anyone else?

i. What did they say about it?

ii. How did this influence you?

m. Did you try to find out any more information about the study?

n. At what point did you decide you wouldn’t respond to the letter? What were your thoughts and feelings at that stage?

o. What were your reasons for not replying to the letter?

i. Too busy/unable to attend/could not read the letter?

ii. Worries about the test e.g. fear of needles?

iii. Worries about lung cancer e.g. fear of the test being positive?

iv. Invitation materials not good enough?

v. Advice from others e.g. discussion with family or friends?

vi. Previous experience of screening tests?

vii. Taking part would not benefit me e.g. may have been in untested group?

Sub-study 2: Telephone Interview Schedule

Aim: To explore understanding and knowledge of the information communicated in the ECLS Study invitation mailing

2. The blood test offered to you is a new test. It can sometimes be difficult to understand what a test like this does and what the result means. It is important for us to see what people understood from the information they read. Please tell me everything you understand about the test you were offered. Tell me as much as you can and if you don't know or you are unsure, it's fine to say so.

Prompts:

a) What do you understand about what the test does?

b) What do you understand about how the test is done?

c) What do you understand about how good is the test at finding lung cancer?

d) What do you understand about the risks of having the test? In other words, any bad things that could happen?

e) What is your understanding of what it means if somebody gets a positive test result?

Aim: To explore thoughts about how the ECLS Study invitation materials could be improved.

3. If you were in charge of writing to people to ask them to have the test, what would you think was important to put in the letter and leaflet? What would you change about the way you were invited and the invitation letter?

4. Is there anything else you would like to tell me about what we have talked about?

Sub-study 3: Telephone Interview Schedule

Please note: This is an interview guide. The questions posed will relate to this guide, but the exact questions will be formulated based on the individual's responses to previous questions during the interview and on the basis of the preceding interviews with other participants. This iterative process is required when using qualitative methods to explore themes fully.

Aim: To explore changes in attitudes to smoking

1) Before you joined the Early Lung Cancer Detection Study, how did you think and feel about smoking?

2) Had you ever tried to stop smoking before you joined the study? If so, how did you find it?

Prompts:

a) How easy or difficult did you find it?

b) Did you use a stop smoking support service or ask for any help to stop smoking?

c) How long did you stop smoking for and how did you feel about that?

3) How do you feel about smoking now?

Prompt:

a) If different from Q.1 – Why do you think your feelings have changed?

Aim: To establish the decisions made regarding smoking cessation during the ECLS Study, the success of those decisions and explore the reasons for those decisions and the perceived barriers and facilitators to cessation.

Questions are worded differently depending on whether the participant was successful at stopping smoking ('Stopped'), unsuccessful at stopping smoking ('Tried') or made no attempt to stop smoking ('No attempt').

4) Stopped: You told us you have stopped smoking since you had the lung blood test. Is this correct?

Tried: You told us you have tried to stop smoking since you had the lung blood test but you didn't manage to stop smoking. Is this correct?

No attempt: Some smokers try to stop smoking and other people choose to carry on smoking without trying to stop. You told us you have not tried to stop smoking since you had the lung blood test and you are still a smoker. Is this correct?

a) If not correct, clarify the decisions made to stop smoking and the success of those decisions i.e. stopped/relapsed/tried but didn't stop.

5) Stopped & Tried: Can you tell me about your decision to try/stop smoking?

Prompts:

a) Which method(s) did you use to try to stop smoking?

b) How easy or difficult was it for you to try to stop smoking after the lung cancer blood test?

c) What do you feel helped you to try to stop smoking?

d) Which things did you feel did not help you to try to stop smoking?

No attempt: What thoughts and feelings did you have about smoking after your lung cancer blood test?

Prompts:

a) Did the lung cancer blood test change your thoughts and feelings about smoking? If so, how?

b) We know that some people find that having a lung cancer blood test makes them want to stop smoking but other people find that it doesn't. It is important for us to understand why this is. Based on your experience of having a lung cancer blood test, why do you think this is?

Aim: To explore thoughts and feelings about smoking cessation advice for lung cancer screening patients.

6) Imagine you are having a lung cancer blood test for the first time, but this time everybody who has the test is given special advice and support about stopping smoking. How would this make you feel about smoking?

Prompts:

a) Would it make you think or feel differently about having the lung cancer blood test if you knew this was going to happen? If so, how?

b) Would it make you think or feel differently about stopping smoking? If so, how?

c) Would it change how confident you felt about being able to stop smoking? If so, how?

d) Would it change your plans to stop or carry on smoking? If so, how?

e) Would it make you more likely or less likely to have a repeat lung cancer blood test in the future e.g. five years time? And why?

7) What type of special advice & support would you find most helpful, if it was given to you during a visit for a lung cancer blood test?

8) Is there anything else you would like to tell me about the things we have talked about?

Please note: This is an interview guide. The questions posed will relate to this guide but the exact questions will be formulated based on the individual's responses to previous questions during the interview, and on the basis of the preceding interviews with other participants. This iterative process is required when using qualitative methods to explore themes fully.

1. Since your lung cancer blood test, have you had any other tests for lung cancer?

2. Before you had the lung cancer blood test, what were you told about it?

3. How satisfied were you with the information you received about the test?

4. Since you have had other tests for lung cancer, how has your understanding about this lung cancer blood test changed? (Question 4 is for participants in the EarlyCDT-positive group who have had further tests for lung cancer).

5. If somebody asked you what having the lung cancer blood test was like, how would you describe it to them based on your experience?

6. How do you think lung cancer is found in the blood?

7. If somebody asked you to tell them about the letter you got about your lung cancer blood test result, how would you describe it to them? How satisfied were you with this letter, in terms of giving you the information you needed to understand the test results. (Question 7 is for participants in the EarlyCDT-negative group)

8. If somebody asked you to tell them about the appointment when you were given your lung cancer blood test result, how would you describe it to them based on your experience? (Question 8 is for participants in the EarlyCDT-positive group)

9. Your lung cancer blood test result was negative. What does a negative blood test result mean to you? (Question 9 is for participants in the EarlyCDT-negative group)

10. Your lung cancer blood test result was positive. What does a positive blood test result mean to you? (Question 10 is for participants who received a positive test result but have not yet had further tests for lung cancer)

11. Since you have had other tests for lung cancer, how have your thoughts about your lung cancer blood test result changed? (Question 11 is for participants who received a positive test result and have had further tests for lung cancer)

End of interview

- We have now come to the end of the interview. Before I switch off the recorder, is there anything else that you would like to tell me?

General prompts to use throughout interview:

- Can you please tell me more about that …
- How has that changed …
- Can you give me an example of …
- What is your understanding of …

APPENDIX 3: **ECLS STUDY: START RECRUITMENT SUBSTUDY (Version 1, 6/1/2014)**

MRC START in ECLS: What are the effects of a re-written and re-designed Participant Information Sheet?

NB: This sub-study was implemented when the recruitment target was 10,000 from NHS Tayside and NHS Glasgow. This study is now complete.

1. Background

In the UK, the NIHR vision sees 'more patients and health professionals participating in health research' [1]. Fundamental to health research is the testing of interventions through Randomised Controlled Trials (RCTs). Achieving high participation in RCTs has traditionally been difficult. Published data show that a minority of RCTs recruit successfully [2,3]. Recruitment problems reduce the total recruited sample (limiting internal validity), and the proportion of eligible participants who are recruited (limiting external validity). They can increase the cost of the study and delay the results. In extreme cases, poor recruitment can result in the cancellation of a trial.

Clearly, there is a need to develop and test interventions to improve recruitment, and one method is to 'nest' trials of recruitment interventions in ongoing RCTs. Given the consensus among the research community concerning the challenge of recruitment, it is surprising that nested trials of recruitment interventions are so rare. Two recent reviews identified only 14 nested studies in real trials [4] and 27 overall [5]. Recruitment for science is not underpinned by a science of recruitment.

The MRC START study is designed to develop the conceptual, methodological and logistical framework for nested studies, and to assess their feasibility. At the completion of MRC START, we will have rigorously tested two potential interventions for adoption in to routine practice (improved participant information sheets (PIS), and a multimedia decision aid), and provided the framework to

make delivery of nested recruitment RCTs a routine activity. This will assist the rapid development of recruitment to meet policy goals [12].

The Early Cancer Detection Test – Lung Cancer Scotland (ECLS) is acting as a host trial to test an MRC START recruitment intervention. This protocol details the work that will be undertaken for the 'MRC START in ECLS' sub-study.

2. The intervention – Participant information sheets

Research has reported patients' rather patchy understanding at the end of a trial, such as one in five participants not knowing the name of the medicine being tested [6] and similar proportions not knowing that they could withdraw at any time [7]. These findings are confirmed by a systematic review of consent in cancer trials [8] in which aspects such as treatment risks and benefits and the right to withdraw consent, were found to be not well understood. The review concluded that "patients do not appear to be adequately informed" (p.304). A lack of participant knowledge might result from the difficulty in understanding complex information, such as randomisation [9], or because of the way the Participant Information Sheet (PIS) is written. The level of literacy required to understand a study PIS is often higher than that found within the general population [10], and poor information provision may particularly affect older or less educated patients [11].

One promising approach to improving the quality of the written information provided is to develop the PIS through formal User Testing. In this process people in the target group for the trial read the PIS and are then asked to find and show an understanding of key information contained in the sheet. Any identified problems are rectified by the use of clear writing and by changing the way the PIS is laid out and designed. Further User Testing then tests whether the changes have led to improvements to the way the PIS performs. Three small, recent studies suggest that a combination of re-writing, design and testing results in a PIS that works much better to inform potential trial participants and which they prefer [13, 14, 15]. These studies have involved hypothetical settings, with participants being asked to imagine themselves being recruited to a trial, and what remains

unknown is the effect of such changes to the PIS in actual trials. In particular, does an improved PIS impact on either of the quality of informed consent and the rate of recruitment?

3. Host study details

The ECLS trial will evaluate a new test (Early CDT) for lung cancer as part of a potential Scottish lung cancer screening program. The ECLS Trial needs to recruit at least 10,000 participants, chiefly from around Glasgow and Dundee. A key recruitment route will be through postal invitation, which opens up the possibility of using the trial to test MRC START user tested patient information.

The trial is being managed by Tayside Clinical Trials Unit (TCTU) and the trial management team at TCTU has developed a PIS presented in booklet form and referred to in this trial as a participant information brochure PIB. Recruitment consists of the following steps:

1. Potential participants are identified by SPCRN staff from practice lists.
2. Potential participants are sent a GP letter of invitation and a PIB
3. Those responding positively to the invitation (via reply slip, text, email or phone) are then screened for eligibility for the study. Those eligible and consenting are recruited.

ECLS, along with the TCTU PIB, has received ethical approval from the East of Scotland Research Ethics Service REC 1, reference 13/ES/0024.

A revised PIB and GP covering letter will be developed by the MRC START team (Dr Peter Knapp) through User Testing. The content of the original PIB will be retained but it will be re-written and re-designed based on the outcomes of the User Testing process.

Potential participants to the ECLS trial will be randomised to receive either the original or the user-tested PIB and GP invitation letter. It would be useful to know if the revised PIB and covering letter impact on rate of recruitment in comparison with the original PIB. A nested RCT would be the best approach to evaluate its effects.

4. Research Objectives

1. To measure differences in those expressing an interest in participating in the ECLS study as a result of receiving a participant information brochure and covering letter
2. To establish if the number of patients recruited in to ECLS is improved by the use of a participant information brochure and covering letter developed through User Testing, compared to a routine participant information sheet.

5. Method

5.1 Design

The proposed study will use an RCT design. Patients identified from GP lists will be randomly allocated to one of two conditions:

a) Control PIB: the original ECLS participant information brochure and covering letter (approved in 13/ES/0024 on 16/4/2013);

b) Intervention PIB: the user tested participant information brochure and covering letter (approved on [Date] by EoSREC REC 1).

5.2 Inclusion / exclusion criteria

All individuals approached by the ECLS host trial are eligible for this nested recruitment intervention study.

5.3 Recruitment and Randomisation

SPCRN will search practice lists for patients eligible for invitation to participate in the ECLS study. Potential participants identified from GP lists as eligible will be randomly allocated to receive the control or intervention PIB and covering letter in a 1:1 ratio. The randomisation will use a list of random numbers from http://www.random.org/sequences/ with management of random allocation being done by SPCRN staff.

Each potential participant will be allocated a cohort number (from the central patient management system).

Potential participants can express and interest in the ECLS study in a number of ways:

- By returning a reply paid slip to the central patient management system who notify the research team
- By email to the research team
- By text (to the research team)
- By phone to the research team

In each instance the patient cohort number will be recorded.

Anonymised data on numbers of respondents and PIS version will be sent to the MRC START team.

5.4 Control

The control PIS is not a plain text document but is formatted in a more attractive way. It was not, however, developed in a systematic way but relies on the experience of TCTU staff. One of the MRC START investigators, Shaun Treweek did proof-read the content of the control PIS in his former roles as Assistant Director of TCTU and co-investigator on ECLS. None of the other MRC START investigators were involved in the development of the original PIS. This PIS is 32 pages in length and was approved by The East of Scotland Research Ethics Committee REC1 on 16th April 2013, reference 13/ES/0024, as part of the application to conduct the ECLS study.

5.5 Intervention

The revised PIS will contain the same content as the original version but will differ in the way that the information is laid out, written and presented. It will be developed through User Testing with members of the public selected to reflect the target patients for the PIB, who will read and then be asked to find and show an understanding of key facts contained in the PIB. The testing will be undertaken in several rounds: the first round testing the original PIB; then several rounds with

different iterations of the revised PIB until we were confident that the PIB could perform well to inform potential trial participants.

5.6 Outcome measures

The primary outcome will be the number of patients recruited to the ECLS trial from each of the PIB arms.

Secondary outcomes will be:

1. The proportion of patients expressing an interest in participating in the ECLS study in response to each version of the PIB and covering letter
2. The proportion of recruited patients who complete the ECLS screening process from each of the PIB arms.

6. Statistical considerations

6.1 Sample size

MRC START in ECLS is powered to detect a significant improvement in recruitment rate, defined as an absolute increase of five per cent above baseline. Baseline response rates for the first five ECLS practices are around 20% (2/12/2013). Ineligibility, difficulties contacting people etc reduces the 20% response rate to a recruitment rate of around 14%. The recruitment rate is the key rate for the MRC START project. A range of samples sizes at 80% power and alpha 0.05 for a 5% minimum important difference between TCTU PIB and START PIB is shown in the table below.

Response rate (%)			Recruitment (%)		
TCTU PIB	START PIB	Sample size (per arm)	TCTU PIB	START PIB	Sample size (per arm)
17	22	1970 (985)	11	16	1466 (733)
20	25	2188 (1094)	14	19	1728 (864)
23	28	2384 (1192)	17	22	1970 (985)

Based on this table, we will have a sample size of 2000 invitations.

6.2 Analysis

Anonymised and aggregated recruitment data (ie. the number of potential participants sent each PIB and covering letter, the numbers expressing an interest in participation, recruited to ECLS and completing the screening from each group) from ECLS will be sent to the MRC START team in accordance with the MRC START data sharing agreement (see Section 12).

The proportion of participants who express an interest in the study, who are recruited in to the study and who complete the screening process will be calculated for the two groups (control and intervention PIB). The difference between the two proportions will be calculated along with the corresponding 95% confidence interval.

Results from this trial will ultimately be combined in a meta-analysis with response rate data from other host trials participating in the MRC START programme.

7. Ethical issues

Patients will not have the opportunity to give informed consent to enter into the nested recruitment study. This has been approved by NRES Committee Yorkshire and the Humber – South Yorkshire

(REC Reference 11/YH/0271) on the basis that the nested study is not withholding information – just changing the way it is presented.

The nested study (MRC START in ECLS) will be registered by the ECLS trial as a sub-study on *Clinicaltrials.gov*

8. Financial and Insurance Issues

The user testing for the nested trial is funded as part of MRC START which is sponsored by the University of Manchester. It forms a sub-study to the ECLS study, which is co-sponsored by the University of Dundee and Tayside Health Board. Normal NHS indemnity procedures will apply.

9. Project Timetable

Date	Action
Jun 2013	Documentation for the nested study agreed & signed off
Jun/July 2013	User Testing of original PIB and development and testing of revised PIB
Jan 2014	Submission to REC of application for substantive amendment
Feb/Mar 2014	Recruitment to the nested trial begins
May/Jun 2014	Recruitment to the nested trial ends
Jun/Jul 2014	Data cleaning and submission of data set to MRC START team
Jul 2014	Collation of results and analysis, begin write up of trial level paper

10. Dissemination of research

The results of this nested sub-study will be published in a peer-reviewed journal to further improve the evidence base regarding effective recruitment strategies in trials. This publication will be led by

the ECLS team. In addition the data will be included in a meta analysis of all studies recruited to the MRC START programme led by the MRC START team. Dissemination of research findings will be conducted in line with the MRC START authorship arrangements (see Section 13).

11. References

[1] Department of Health. Best Research for Best Health - a new national health research strategy. London. Department of Health, 2006.

[2] McDonald A, Knight R, Campbell M, et al. What influences recruitment to randomised controlled trials? A review of trials funded by two UK funding agencies. *Trials* 2006;7:9

[3] Bower P, Wilson S, Mathers N. How often do UK primary care trials face recruitment delays? *Fam Pract* 2007;24:601-603.

[4] Watson J, Torgerson D. Increasing recruitment to randomised trials: a review of randomised controlled trials. *BMC Med Res Methodol* 2006;6:

[5] Treweek S, Mitchell E, PitKethly M, et al. Strategies to improve recruitment to randomised controlled trials. *Cochrane Database of Systematic Reviews* 2010;Issue 1 Art. No.:MR000013. DOI: 10.1002/14651858.MR000013.pub4:

[6] Griffin JM, Struve JK, Collins D, Liu A, Nelson DB, Bloomfield HE. Long term clinical trials: how much information do participants retain from the informed consent process? *Contemporary Clinical Trials* 2006; 27(5): 441-8.

[7] Fortun P, West J, Chalkley L, Shonde A, Hawkey C. Recall of informed consent information by healthy volunteers in clinical trials. *Quarterly Journal of Medicine* 2008; 101: 625-9.

[8] Cox AC, Fallowfield LJ, Jenkins VA. Communication and informed consent in phase 1 trials: a review of the literature. *Support Care Cancer* 2006; 14(4): 303-9.

[9] Featherstone K, Donovan JL. Random allocation or allocation at random? Patients' perspectives of participation in a randomised controlled trial. *British Medical Journal* 1998; 317: 1177-80.

[10] Paasche-Orlow MK, Taylor HA, Brancati FL. Readability standards for informed-consent forms as compared with actual readability. *New England Journal of Medicine* 2003; 348: 721-6.

[11] Sugarman J, McCrory DC, Hubal RC. Getting meaningful informed consent from older adults: a structured literature review of empirical research. *Journal of the American Geriatric Society* 1998; 46(4): 517-24.

[12] Department of Health. Equity and excellence: liberating the NHS. London. The Stationary Office, 2010.

[13] Knapp P, Raynor DK, Silcock J, Parkinson B. Performance-based readability testing of participant materials for a Phase 1 trial: TGN1412. *Journal of Medical Ethics* 2009 35: 573-8.

[14] Knapp P, Raynor DK, Silcock J, Parkinson B. Performance-based readability testing of participant materials for a Phase 3 IVF trial. *Trials* 2009; 10: 79.

[15] P Knapp, DK Raynor, J Silcock, B Parkinson. Can user testing of a clinical trial patient information sheet make it fit-for-purpose? – a randomised controlled trial. *BMC Medicine* 2011, 9:89. doi:10.1186/1741-7015-9-89.

12. MRC START Data Sharing Agreement

MANCHESTER 1824

MRC START Data sharing agreement

This document specifies the data management and data sharing agreement between the MRC START study and the ECLS study.

In this document, the ‘START research team’ refers to researchers named on the protocol. ‘START collaborators’ refers to those providing ‘host’ trials for the study.

MRC START roles and responsibilities

ECLS team agrees to:

(a) Randomly allocate a proportion of patients participating in ECLS to receive either the standard participant information brochure or the user tested patient information brochure. Both baseline response rates and baseline recruitment are uncertain at this point. A range of samples sizes at 80% power and alpha 0.05 for a 5% minimum important difference between the Tayside Clinical Trials Unit (TCTU) PIB and the MRC START PIB have been calculated and the final sample size for the MRC START sub-study will be determined by the response rate achieved in the early stages of the ECLS recruitment as per the MRC START in ECLS protocol.

(b) Randomise patients to either the recruitment intervention using random number generation within the SQL software used for the ECLS patient management system.

(c) Collect data on the numbers of patients approached using each recruitment method, and data on the numbers recruited to the trial, and the number retained at each follow up point as follows:

  a. Expressing interest (responding to either PIB)
  b. Attending screening
  c. Being consented in to the trial
  d. Completing the trial

(d) Provide collected data in an anonymised form (labelled data set in SPSS or a data base suitable for import to SPSS) to the START research team for analysis by [*target date for data collection tbc*]

(e) Not introduce the recruitment intervention in a non-randomised fashion during MRC START

(f) Seek permission from the MRC START research team to introduce them after the end of the MRC START study period.

It is possible that host trials may wish to withdraw from MRC START before the end of the study. In this case, data collected up to that point would still be provided to the MRC START research team.

Data Protection and publication issues in the START study

The University of Manchester has strict guidelines for data storage, access to study data and adherence to the principles of data protection (including the Data Protection Act 1998). The link to relevant information is:

http://www.staffnet.manchester.ac.uk/services/records-management/data-protection/data-protection-guidance/

Data Transfer Policy

Datasets will be accepted from MRC START collaborators in electronic format (the University of Manchester can translate datasets in various formats through *Stat Transfer*). In addition, MRC START collaborators will provide written details of the coding of variables in the dataset to allow consistent analysis (see study protocol).

All datasets will be anonymised by MRC START collaborators *before* transfer to the University of Manchester, removing all identifiable patient information such as names and addresses. Data may be encrypted before transmission to ensure security.

Data storage

Datasets from MRC START collaborators will be transferred to a combined database on a secure server at the Health Sciences Research Group, University of Manchester. All data received will be treated in the strictest confidence. Analysis of the data will take place by Professor Peter Bower and Professor Sandra Eldridge. Professor Bower will act as custodian for the combined dataset. The combined dataset will be stored by the University of Manchester in a secure location. Data from individual datasets will remain the property of MRC START collaborators.

Environment

The NIHR School for Primary Care Research (http://www.haps.bham.ac.uk/primarycare/nspcr/index.shtml) comprises the leading academic centres for primary care research in England, with a focus on research to improve everyday practice in primary care. The MRC START research project is led by the Centre for Primary Care, Institute of Population Health, the University of Manchester (http://www.population-health.manchester.ac.uk/research/primarycare/)

13 MRC START Authorship Arrangements

MRC START publications & authorship arrangements

MRC START has the potential to generate a large number of publishable datasets, which will include nested trials of MRC START interventions run in single trials ('single datasets'), and the combined datasets of MRC START interventions run in multiple trials ('combined datasets').

This document describes the ground rules for publishing and authorship for applicants and researchers on the MRC START grant ('START research team') and researchers providing 'host' trials for the study ('START collaborators').

Core Principles

The core principle governing authorship are: clear communication; no surprises; no waiting to publish; and access to an independent adviser.

Ground rules:

1. All publications arising from the 'combined datasets' will include the START research team and representatives from START collaborators (normally host trial PI).
    a) Where START collaborators request more than one representative, nominations for authorship will be discussed among the START research team.
    b) Requirements for authorship are those of the International Committee of Medical Journal Editors (http://www.icmje.org/).
    c) If author numbers become excessive, papers may be authored under a collaborative name or a combination of named authors (START research team) and a group collaborative name (START collaborators) (http://www.councilscienceeditors.org/i4a/pages/index.cfm?pageid=3373).
2. The START research team are keen to encourage publication from single datasets where possible.
    a) Publication of the final MRC START data takes precedence – we cannot delay publication, for example, to allow single datasets to be published first.
    b) We would expect that START collaborators would look for opportunities to involve members of the START research team as authors in publications arising from individual datasets, either as individuals or under a collective name.
    c) The START research team will be able to provide materials for papers on the development of the interventions, as well as general background and criteria for reporting standards in nested trials developed as part for the MRC START project.
3. All other publications arising from MRC START (ie not based on the combined datasets) remain in the authorship of the START research team
4. START collaborators need to sign up to the MRC START authorship arrangements.

5. We will appoint an independent adviser to whom the START research team or START collaborators can go for advice or independent arbitration in the event of a disagreement about authorship.

References

1. Raffle AE, Gray JAM. Screening: Evidence and practice: Oxford University Press, USA; 2007.
2. Khalid-de Bakker C, Jonkers D, Smits K, Mesters I, Masclee A, Stockbrugger R. Participation in colorectal cancer screening trials after first-time invitation: a systematic review. *Endoscopy* 2011; **43**(12): 1059-69.
3. Palencia L, Espelt A, Rodriguez-Sanz M, et al. Socio-economic inequalities in breast and cervical cancer screening practices in Europe: influence of the type of screening program. *International Journal of Epidemiology* 2010; **39**(3): 757-65.
4. van den Bergh KAM, Essink-Bot ML, van Klaveren RJ, de Koning HJ. Informed participation in a randomised controlled trial of computed tomography screening for lung cancer. *Eur Resp J* 2009; **34**(3): 711-20.
5. Ferroni E, Camilloni L, Jimenez B, et al. How to increase uptake in oncologic screening: A systematic review of studies comparing population-based screening programs and spontaneous access. *Preventive Medicine* 2012; **55**(6): 587-96.
6. Jepson R, Clegg A, Forbes C, Lewis R, Sowden A, Kleijnen J. The determinants of screening uptake and interventions for increasing uptake: a systematic review. *Health Technology Assessment (Winchester, England)* 2000; **4**(14): i-vii, 1-133.
7. Chapple A, Ziebland S, McPherson A. Stigma, shame, and blame experienced by participant s with lung cancer: qualitative study. *British Medical Journal* 2004; **328**(7454): 1470-3.
8. Patel D, Akporobaro A, Chinyanganya N, et al. Attitudes to participation in a lung cancer screening trial: a qualitative study. *Thorax* 2012; **67**(5): 418-25.

9. Silvestri GA, Nietert PJ, Zoller J, Carter C, Bradford D. Attitudes towards screening for lung cancer among smokers and their non-smoking counterparts. *Thorax* 2007; **62**(2): 126-30.

10. Michie S, McDonald V, Marteau TM. Understanding responses to predictive genetic testing: A grounded theory approach. *Psychology and Health* 1996; **11**: 455-70.

11. Michie S, Smith JA, Senior V, Marteau TM. Understanding Why Negative Genetic Test Results Sometimes Fail to Reassure. *American Journal of Medical Genetics* 2003; **119**: 340-7.

12. Michie S, Weinman J, Miller J, Collins V, Halliday J, Marteau TM. Predictive genetic testing: High risk expectations in the face of low risk information. *Journal of Behavioural Medicine* 2002; **25**: 33-50.

13. Maissi E, Marteau TM, Hankins M, Moss S, Legood R, Gray A. Psychological impact of human papillomavirus testing in women with borderline or mildly dyskaryotic cervical smear test results: cross sectional questionnaire study. *British Medical Journal* 2004; **328**(7451): 1293-9.

14. Fylan F. Screening for cervical cancer: a review of women’s attitudes, knowledge and behaviour. *British Journal of General Practice* 1998; **48**: 1509-14.

15. French DP, Maissi E, Marteau TM. Psychological costs of inadequate cervical smear test results. *British Journal of Cancer* 2004; **91**: 1887-92.

16. French DP, Maissi E, Marteau TM. Psychological costs of inadequate cervical smear test results: three-month follow-up. *Psycho-oncology* 2006; **15**(6): 498-508.

17. Brett J, Austoker J. Women who are recalled for further investigation for breast screening: psychological consequences 3 years after recall and factors influencing re-attendance. *Journal of Public Health Medicine* 2001; **23**: 292-300.

18. Hahn EJ, Rayens MK, Hopenhayn C, Christian WJ. Perceived risk and interest in screening for lung cancer among current and former smokers. *Research in Nursing & Health* 2006; **29**(4): 359-70.

19. van der Aalst CM, van den Bergh KAM, Willemsen MC, de Koning HJ, van Klaveren RJ. Lung cancer screening and smoking abstinence: 2 year follow-up data from the Dutch-Belgian randomised controlled lung cancer screening trial. *Thorax* 2010; **65**(7): 600-5.

20. Ashraf H, Tonnesen P, Pedersen JH, Dirksen A, Thorsen H, Dossing M. Effect of CT screening on smoking habits at 1-year follow-up in the Danish Lung Cancer Screening Trial (DLCST). *Thorax* 2009; **64**(5): 388-92.
21. Taylor KL, Cox LS, Zincke N, Mehta L, McGuire C, Gelmann E. Lung cancer screening as a teachable moment for smoking cessation. *Lung Cancer* 2007; **56**(1): 125-34.
22. Deutekom M, Vansenne F, McCaffery K, Essink-Bot M-L, Stronks K, Bossuyt PMM. The effects of screening on health behaviour: a summary of the results of randomised controlled trials. *Journal of Public Health* 2011; **33**(1): 71-9.
23. Bach PB, Mirkin JN, Oliver TK, et al. Benefits and Harms of CT Screening for Lung Cancer A Systematic Review. *JAMA-J Am Med Assoc* 2012; **307**(22): 2418-29.
24. Townsend CO, Clark MM, Jett JR, et al. Relation between smoking cessation and receiving results from three annual spiral chest computed tomography scans for lung carcinoma screening. *Cancer* 2005; **103**(10): 2154-62.
25. Roddy E, Antoniak M, Britton J, Molyneux A, Lewis S. Barriers and motivators to gaining access to smoking cessation services amongst deprived smokers - a qualitative study. *BMC Health Services Research* 2006; **6**.
26. Jackson N, Prebble A. Perceptions of smoking cessation: Products and services among low income smokers. London: Health Development Agency, 2002.
27. Wiltshire S, Bancroft A, Parry O, Amos A. 'I came back here and started smoking again': perceptions and experiences of quitting among disadvantaged smokers. *Health Education Research* 2003; **18**(3): 292-303.
28. Ostroff J. Lung Cancer Screening and Cessation. National Cancer Policy Forum Workshop on Reducing Tobacco-Related Cancer Incidence and Mortality; 2012; Washington, USA: Institute of Medicine of the National Academies; 2012.
29. Onwuegbuzie AJ, Leech NL. A Call for Qualitative Power Analyses. *Quality & Quantity* 2007; **41**: 105-21.

30. Maykut P, Morehouse R. Beginning Qualitative Research: A Philosophic and Practical Guide. *London: Routledge Falmer* 2000.

31. Knapp P, Raynor DK, Silcock J, Parkinson B. Can user testing of a clinical trial participant information sheet make it fit-for-purpose? – a randomised controlled trial. *BMC Medicine* 2011; **9**: 89.

32. McCaffery K, Borril J, Williamson S, et al. Declining the offer of flexible sigmoidoscopy screening for bowel cancer: a qualitative investigation of the decision-making process. *Social Science & Medicine* 2001; **53**(5): 679-91.

33. Chapple A, Ziebland S, Hewitson P, McPherson A. What affects the uptake of screening for bowel cancer using a faecal occult blood test (FOBt): A qualitative study. *Social Science & Medicine* 2008; **66**(12): 2425-35.

34. Braun V, Clarke V. Using thematic analysis in psychology. *Qualitative Research in Psychology* 2006; **3**(2): 77-101.

APPENDIX 4: Sub Study 5: University Of Glasgow Mrc Phd Studentship.

Exploring public perceptions of lung cancer screening

Researcher: Hannah Scobie

Supervisors: Dr Katie Robb, Dr Sara MacDonald, Professor Sally Wyke, Dr Stephen Harrow. University of Glasgow

Funder: Medical Research Council

1. Introduction

1.1 Rationale

Lung cancer kills more people than any other cancer, with approximately 5,000 people dying from lung cancer every year in Scotland. This is often because there are few symptoms until the cancer is at an advanced stage when the chance of cure is low. Lung screening offers the potential to detect lung cancers at an earlier stage when they are easier to treat. A recent trial in the US found that lung cancer mortality decreased by 20% among those receiving low dose computed tomography screening (Aberle, Adams, Berg, Black, Clapp & Fagerstrom, 2011). However, the benefits of cancer screening are only realised if people are willing to participate. Cancer screening participation rates remain suboptimal (Audit Scotland, 2012), and may be particularly challenging in the case of lung screening. Smokers are disproportionately represented among people living in more deprived areas who also have lower uptake of other cancer screening programmes (Scottish Household Survey, 2013). This means that the potential lung screening target population could be particularly hard-to-reach.

1.2 Proposed research

The proposed research consists of two further sub-studies within the Early Cancer detection test – Lung cancer Scotland (ECLS) Trial. The first sub-study will qualitatively investigate why individuals decided not to take part in the ECLS Trial, after showing initial interest. This study (Study 1) will involve interviewing ECLS Trial 'non-attenders' – those who initially expressed an interest in having the test,

were appointed to be screened, but later decided not to participate. It is intended that up to a total of 20 men and women non-attenders in the ECLS trial will be interviewed. The sample will be drawn from the NHS Greater Glasgow and Clyde and NHS Lanarkshire Health Boards.

The second proposed sub-study (Study 2) will be a quantitative analysis of ECLS Trial attenders examining potential demographic and psychosocial differences by recruitment type. Participants in the ECLS Trial were recruited by two strategies: i) those who were invited to take part via their General Practice (GP) or; ii) those who 'self-selected' after seeing community advertisement/media releases or responded as a result of word of mouth. This study will examine potential differences in the demographic characteristics, beliefs about lung cancer and lung cancer screening, subjective health and risk perceptions among these two groups.

The proposed studies will complement the embedded psychological sub-studies currently being conducted by researchers at the University of Nottingham including: emotional and behavioural responses following screening; exploring why people declined to participate; understanding of screening results; and smoking cessation in participants of the lung screening Trial. The proposed work therefore adds two new aspects to the ECLS Trial research by considering; i) why people change their mind about participating in the Trial; and ii) exploring any potential differences between participants recruited through GPs and 'self-selecters'.

2. STUDY 1

2.1 Background & Literature Review

While it is noted that participating in a screening Trial is not the same as participating in a screening programme, it is useful to draw from the literature on cancer screening programme participation in helping to understand screening behavior. When participants make an appointment for cancer screening, it suggests they are motivated and intend to go to the screening appointment. However,

this intention to attend does not always translate into action (i.e. attending the appointment) and 'did not attend' (DNA) and cancellations are frequent outcomes at screening clinics (Sheeran, 2002). Within the psychological literature. Orbell & Sheeran (1998) used the term inclined abstainers. To describe people with positive intentions who fail to act.

In the context of the present study, participants who initially make an appointment (positive intention), but go on to cancel or do not attend their appointment would be considered to be inclined abstainers. It is this group who are the primary interest of Study 1.

Among the small number of studies on psychosocial barriers to lung cancer screening, cancer fatalism appears to play a significant role in uptake. A qualitative study in England exploring attitudes towards participation in lung cancer screening found themes of fatalism, worry, and avoidance in those who declined to be screened (Patel, Akporobora, Chinyanganya, Hackshaw, Seale, Spiro, & Griffiths, 2012). This conclusion was also supported by a quantitative study in the US, where participants who had fatalistic beliefs about lung cancer were less likely to undergo screening (Jonnalagadda, Bergamo, Lin, Lurslurchachai, Diefenbach, Smith, Nelson & Wisnivesky, 2012). Other barriers to lung cancer screening included: denial of risk, shame about smoking, fears about screening and embarrassment (Walton, McNeil, Stevens, Murray, Lewis, Aitken & Garrett, 2013).

Understanding the socio-demographic characteristics of attenders and non-attenders of cancer screening is crucial to ensure the introduction of a screening programme does not exacerbate health inequalities. For example, those from more deprived groups may be less likely to attend cancer screening (Weller & Campbell, 2009; Moser, Patnick & Beral (2009), but have a higher risk of cancer due to e.g. smoking, unhealthy diet, sedentary lifestyle. Other socio-demographic characteristics that may play a role in cancer screening attendance include age and gender.

2.2 Potential Risks & Benefits

Risks - This study is low risk, however there are a few areas to consider as potentially problematic. Study 1 (invitation Strategy 2) will involve writing to potential participants in some cases 6 months or more after they did not attend their appointment. It is possible that individual circumstances may have changed within this time. In some circumstances it is possible participants may have passed away or become unwell. As a result, Health Informatics (HIC) University of Dundee will check against the patients CHI number through NHS health records to see if participants are still alive. In addition, the Study 1, Strategy 2 invitation letter will include the sentence: 'We apologise if this letter arrives at a particularly difficult time for you.'

Another potential area of risk could be the topic of the study. We are discussing a health issue and cancer in particular, which might upset some participants. This will be avoided by reminding the participant that they are under no obligation to answer all of the questions and may stop the discussion at any point. Moreover, the interview will be flexible enough to allow participants to introduce information that they feel comfortable with. If the participant appears hesitant or in doubt about responding, the interviewer will give them some time to proceed, alter the question or move on. Finally, we will provide the telephone number and email address of the researcher at the end of the interview in case participants wish to talk about any of the issues raised in the interview. If necessary the researcher will refer participants to one of the project supervisors to provided further information or support. If required, the supervisor will provide details for professional organisations for people who feel they need to discuss issues further.

Benefits –There are few potential benefits to research participants although in the past some participants in similar studies have reported enjoying the opportunity to take part in research. Those who participate in the interviews will be offered a £20 voucher as a token of appreciation for their

participation (Appendix A). Participants will be required to sign for the voucher received at the end of the interview. If the participant wishes to withdraw from the interview at any point during the interview, the participant will still receive the voucher.

2.3 Aim

The aim of Study 1 is to explore the beliefs and perceptions about lung cancer and lung screening among people who initially expressed an interest in screening, were appointed to be screened, but who later cancelled or did not attend their appointment, and in some cases did not attend a reappointment.

Methodology

2.4 Inclusion Criteria

Participants are required to have been invited and subsequently been eligible to participate in the ECLS Trial. Further, participants will have shown initial interest in the study, but at a later time, declined to participate. See Table 1 for further details.

2.5 Exclusion Criteria

Participants who were invited to take part in the ECLS trial, and completed the study. Also, inability to speak, read or write English. The study involves understanding a Participant Information Sheet, completing a consent form and taking part in an interview in English. People who are unable to speak, read or write English will therefore be excluded most likely because they will not have responded to the initial invite to take part in the Trial. See Table 1 for further details.

Table 1: Study 1 Inclusion / Exclusion Criteria	
Inclusion	Exclusion
Invited to take part in the ECLS trial	Inability to speak, read or write English

Eligible to take part in ECLS trial on reassessment	Individuals who contacted the team for information, but did not make an appointment
Participants who made an appointment, but subsequently cancelled or DNA	Individuals whose eligibility to take part in the ECLS trial was not established
	Participants who cancelled or DNA, but rescheduled another appointment for a later date and attended.

2.6 Study Design

Interviews will be conducted face-to-face in the participants' own homes or at the University of Glasgow, or over the telephone, whichever is most convenient to the participant. Participants' travel expenses will be reimbursed if they choose to come to the University of Glasgow. It is recognised that the researcher will be working alone. As a result, the University of Glasgow's policy on lone working will be followed to ensure the safety of the researcher and participant.

Participants will receive the Participant Information Leaflet and informed consent form with their letter of invitation by post before the interview is conducted. Contact numbers are given to contact the study team to answer any questions they may have. In the case of telephone interviews being the preferred interview format, participants are invited to send their reply and completed consent form in the prepaid envelope. Prior to the telephone interview they will be asked to confirm their verbal consent. Participants will be offered the opportunity to ask any questions about the study before informed consent is taken by the researcher. The researcher will seek consent in the first instance. Interviews will last approximately 1 hour and will be based on a topic guide (Appendix B) developed from the existing screening literature with a particular focus on barriers to cancer screening. To avoid post-hoc rationalisations of their screening behaviour we will ask participants to discuss their general

views on screening first before moving on to their personal experience. With the permission of the interviewee, interviews will be audio-recorded and transcribed verbatim. If the participant does not consent to be recorded, the participant can continue with the interview with the researcher taking detailed notes instead. Data from interviews will be anonymised during the transcription process. Thereafter paper copies of the transcripts will be stored in locked filing cabinets at General Practice & Primary Care, University of Glasgow. Interview transcripts will be assigned unique identifiers and any quotations that may be used with publications or reports will use the unique identifier. As such individual participants will not be identified.

2.7 Researcher Effects

Researcher effects will be kept to a minimum by using a topic guide to ensure participants are asked the same questions. However, due to the nature of qualitative research, supplementary questions may vary depending on the responses of the participants.

2.8 Duration of Participation

Participants will be asked to take part in one qualitative interview lasting approximately one hour. The research team will not contact the participant again, although study results will be disseminated to the individual following completion of the study if requested. If participant request the study results, their name and address will be noted. Participants requesting the results will be mailed a summary of the main findings. The study results will also be disseminated through the normal academic channels, including, publications and conference presentations.

2.9 Criteria for Discontinuation

Study 1 involves a one off interview and this will be the only contact with the research team. If informed consent is taken at the time of interview and the participant completes the interview, the research team will have no further contact with the research participant. If a participant decides part

way through the interview to withdraw from this study the data collected would be retained if permission is given. If no permission is given, the data will be withdrawn.

If participants make an appointment with the researcher, and cancel or DNA the researcher will attempt to make contact again. Appointments will be rearranged up to three times. If a participant is unable to make the interview after the third attempt of rearranging an appointment they will be removed from the invitation list.

2.10 Procedure for collecting data

This will be a difficult group to engage, as a result, three recruitment strategies will be used:

1a. It is normal practice that the ECLS study team call participants the day before their appointment as a reminder in an attempt to reduce the number of DNAs. If during this call a potential participant states they wish to withdraw from the Trial the study team will ask the participant if they would be interested in taking part in a research project for people who decide not to attend their appointment. If participants express an interest they will be asked if they agree for a member of the research team to contact them directly to provide more information about the research. The participant will be reassured if they wish to decline and no further contact will be made by the research team.

1b. Within the ECLS Trial protocol, if a participant DNA, the study team will call the participant to offer a new appointment time. If during this call the participant states they wish to withdraw from the Trial, the study team will ask the participant if they would be interested in taking part in a research project and the procedure would be as described in 1a.

2) We will retrospectively identify and contact people who booked an appointment, accepted an appointment, but cancelled or DNA initially within the previous 12 months (i.e. 1 year from the commencement of the sub-study). If insufficient participants respond, we will contact people from

the beginning of the Trial in Glasgow. Participants will be identified from the Patient Management System used by the ECLS Trial. Eligible participants will be identified by the researcher, searching the additional text related to each case for key words such as, ‘cancelled’, ‘did not attend’ or ‘DNA’. Once participants have been identified, the Health Informatics Centre (HIC at Dundee University) will extract the names and addresses of those eligible.

Participants will be contacted by post after they have been identified as a suitable candidate via HIC. Invitation letters will be sent out via a mail merge at HIC and those identified by HIC as having died will be excluded. Participants will be given a reply slip to return if they would like the researcher to contact them. Alternatively they can contact the researcher by telephone or email. The researcher will not know the identity of the participant until the reply slip stating that they wish to participate is returned.

2.11 Data Protection

When potential participants express an interest, contact details will be stored in a locked filing cabinet at the University of Glasgow. Consent forms will similarly be stored in locked filing cabinets. Data from interviews will be digitally recorded and recordings will be uploaded to password protected university computers. The recordings will be assigned a unique ID number rather than the participant name. Thereafter paper copies of transcripts will also be stored in locked filing cabinets at the University of Glasgow. Any direct quotations that may be used with publications or reports will use the unique identifier. As such individual participants will not be identified. Data will be retained for 10 years after the study is completed.

Statistical Considerations

2.12 Sample Size

We will undertake interviews with a sample of approximately 20 ECLS Trial non-attenders. Based on previous literature, this is the likely number required to reach 'saturation' in terms of identification of new themes/ideas/issues. Based on previous experience, in order to obtain a sample of 20 participants, around 400 people may need to be contacted although this may be less depending on the success of Strategies 1a and b. The study aims to interview a mix of males and females. If possible a sampling frame will be used so the balance of gender reflects the ratio of men to women among the DNA group overall. However, we anticipate that it will be challenging to obtain 20 participants so this may not be possible.

2.13 Method of Analysis

The data will be analysed using the `framework approach´, a type of thematic analysis. Thematic analysis is a method for identifying, analysing, and reporting recurring patterns within data, which can then be reported in a detailed way. The demographic characteristics of the participants including age, gender and Scottish Index of Multiple Deprivation score will also be described.

3. STUDY 2

3.1 Background & literature review

The ECLS trial recruits participants in two distinct ways: i) invitation via GP or; ii) through community advertisement/ media releases/word of mouth and website review. As a result, it may be possible that there are sociodemographic and psychosocial differences between the participants who were invited by their GP and those who self-selected to participate.

Previous research in lung cancer screening indicates that there are significant differences between participants who are invited to take part, and those who self-select. Participants in the US National

Lung Screening Trial, who were recruited by the media, appeared to be younger, higher educated and less likely to be current smokers (NLST, 2010). Similarly, in the Dutch–Belgian Lung Cancer Screening Trial (NELSON trial), respondents to the initial invitation were somewhat younger, and less likely to be current smokers (van der Aalst et al., 2012).

Similar results can also be found outside lung cancer screening trials. In the Oslo Health Study, respondents to community and media advertisement were associated with older age, higher education levels, being married, and also not in receipt of benefits (S�gaard, Selmer, Bjertness & Thelle, 2004). A secondary analysis of the Malmo Diet and Cancer Study concurs with the results of S�gaard et al. (2004). When comparing the respondents of community invitations and personal invitations, Manjer et al. (2002) found that community respondents were older, and more often females, than participants recruited using personal invitations. Furthermore, participants recruited through community advertisement had a comparably more favourable situation with regard to sociodemographic and lifestyle factors. They also had a lower frequency of prevalent disease, lower incidence of cancer and lower mortality (Manjer, Elmsta, Janzon&Berglund, 2002).

The present ECLS study will examine potential differences between the two invitation groups of the ECLS trial. This will assist with the future development of more efficient invitation strategies that will target the most high risk groups.

3.2 Aim

The primary aim of Study 2 is to explore if there are any sociodemographic or psychosocial differences as assessed by a baseline questionnaire between participants of the ECLS study who were invited by GP or self-selected through community advertising.

Methodology

3.3 Inclusion Criteria

In order to be included within the statistical analyses, participants are required to have taken part in the ECLS trial, and completed the baseline study questionnaire.

3.4 Exclusion Criteria

Participants who took part in the ECLS trial, but did not complete the study questionnaire will be excluded from the analysis.

3.5 Procedure for identifying participants

Participants will be identified from the patient management system (PMS) used by the ECLS trial. Eligible participants for the analyses will be identified by their invitation type group (GP or self-select). Once cases have been identified, the anonymised data required including demographic characteristics (age, gender, Scottish Index of Multiple Deprivation) and the responses to the psychosocial questionnaire will be extracted from OpenClinica. Data will be extracted using participants' cohort ID.

3.6 Study Design

The required anonymized data will be extracted from study data base; Open Clinica in order to complete the analysis. Data will be analysed at the University of Glasgow. The data will be transferred and stored as per the Data Sharing Agreement. The data will be analysed using Microsoft Excel 2010 and IBM SPSS version 21, provided by the University of Glasgow.

Statistical Considerations

3.7 Sample Size

This sub study will analyse the data from all attenders of the ECLS Trial.

3.8 Method of Analysis

Statistical analysis will be conducted using IBM SPSS. Participants' base-line data will be compared for the two groups of interest – GP invitation and self-selected. This will include demographic characteristics, beliefs about lung cancer and lung cancer screening, perception of general health and risk perception obtained from the baseline questionnaire.

References

1. Aberle, D.R., Adams, A.M., Berg, C.D., Black, W.C., Clapp, J.D., Fagerstrom, R.M., et al. (2011) Reduced lung-cancer mortality with low-dose computed tomographic screening. New England Journal of Medicine, 365(5), 395-409.
2. Audit Scotland (2012) Health Inequalities in Scotland. Accessed: http://www.audit-scotland.gov.uk/docs/health/2012/nr_121213_health_inequalities.pdf
3. Bradley, C.J., Given, C.W. & Roberts, C. (2002) Race, Socioeconomic Status, and Breast Cancer Treatment and Survival. Journal of the National Cancer Institute, 94(7), 490-496.
4. Chapple, A., Ziebland, S. & McPherson, A. (2004) Stigma, shame, and blame experienced by patients with lung cancer: qualitative study. BMJ Online (accessed: October, 2014).
5. Eiser, J.R. & Cole, N. (2002) Participation in Cervical Screening as a Function of Perceived Risk, Barriers, and Need for Cognitive Closure. Journal of Health Psychology, 7(99), 99-105.
6. Jonnalagadda, S., Bergamo, C., Lin, J.J., Lurslurchachai, L., Diefenbach, M., Smith, C., Nelson,J.E. & Wisnivesky, J.P. (2012) Beliefs and attitudes about lung cancer screening among smokers. Lung Cancer, 77, 526-531.
7. Lo, S.H., Waller, J., Wardle, J. & von Wagner, C. (2013) Comparing barriers to colorectal cancer screening with barriers to breast and cervical screening: a population-based survey of screening-age women in Great Britain. Journal of Medical Screening, 1-7.

8. Manjer, J., Elmstaehl, S., Janzon, L. & Berglund, G. (2002) Invitation to a population-based cohort study: differences between subjects recruited using various strategies. Scandinavian Journal of Public Health, 30, 103-112.

9. Moser, K., Patnick, J. & Beral, V. (2009) Inequalities in reported use of breast and cervical screening in Great Britain: analysis of cross sectional survey data. British Medical Journal,338.

10. National Lung Screening Trial Research Team, Aberle, D.R., Adams, A.M., Berg, C.D., Clapp, J.D., Clingan, K.L., et al.(2010) Baseline characteristics of participants in the randomised national lung screening trial. Journal of the National Cancer Institute, 102,1771–9.

11. Patel, D., Akporobaro, A., Chinyanganya, N., Hackshaw, A., Seale, C., Spiro, S.G. & Griffiths, C. (2012) Attitudes to participation in a lung cancer screening trial: a qualitative study. Thorax, 67, 418-425.

12. Savage, S.A. & Clarke, V.A. (2001) Factors Associated with Breast and Cervical Cancer Screening Behaviours. Health Education, 101(4), 176-186.

13. Scottish Household Survey (2013). Scottish Government. Accessed: http://www.gov.scot/Publications/2014/08/7973

14. Søgaard, A.J., Selmer, R., Bjertness, E. & Thelle, D. (2004) The Oslo Health Study: The impact of self-selection in a large, population-based survey. International Journal for Equity in Health, 3(3).

15. van der Aalst, C.M., van Iersel, C.A., van Klaveren, R.J., Frenken, F.J.M., Fracheboud, J., Otto, S.J., de Jong, P.A., Oudkerk, M. & de Koning, H.J. (2012) Generalisability of the results of the Dutch–Belgian randomised controlled lung cancer CT screening trial (NELSON): Does self-selection play a role? Lung Cancer, 77, 51-77.

16. Walton, L., McNeill, R., Stevens, W., Murray, M., Lewis, C., Aitken, D. & Garrett, J. (2013) Patient perceptions of barriers to the early diagnosis of lung cancer and advice for health service improvement. Family Practice, 30, 436-444.

17. Weller, D.P. & Campbell, C. (2009) Uptake in cancer screening programmes: a priority in cancer control. British Journal of Cancer, 101(2), 55-59.

APPENDIX 4A: ECLS Non-Attenders Interview Schedule (version 1, May 2015)

Study Title: Understanding why people who are initially interested in lung screening fail to participate.

1) General views about cancer screening

What do they think about it, what do they feel about it

How do they think people make decisions about whether to do screening –'know' as soon as invited/think it over/don't know

2) Beliefs about cancer in general and lung cancer

Are they aware of spouse/family/friends taking part in screening?

What comes to mind when you think about:

i. Cancer?
ii. Lung cancer?

Following elicitation of participants' beliefs about, ask how fearful participants are of cancer in general and lung cancer and whether they believe they (lung or other types) can be successfully treated (if these have not come up in response to the first questions).

3) Understanding of the lung screening test

What comes to mind when you think about lung cancer screening?

Following the elicitation of image, ask them to explain how they would explain this image and why they had it.

What is their understanding of what the test involves?

What is their understanding of the purpose of the test – detection/prevention?

4) Personal decision about lung cancer screening participation (show example invitation letters and leaflets to prompt memory)

Do they remember receiving an invitation for the screening test?

As best they can remember, when invitation letter arrived in the post how did they think, how did they feel?

How did they decide what to do next? (e.g. Knew right away what they'd do/thought it over/don't know/remember)

What did they do next? (e.g. Acted immediately, acted after a reminder, forgot, changed mind, didn't get round to it.....)

Did other things happening in life at the time influence decision?

What did they think when decided not to attend the lung screening appointment? How did they feel about it? Were other things happening in their life that influenced their decision?

5) Feelings of risk lung cancer

What do they feel about their chances of getting lung cancer? Do they feel equally at risk/not at risk/higher risk for lung cancer compared to other types of cancer? Do they feel their chances of getting lung cancer is the same or different for other types of cancer? Why?

Who do they think would be at high risk of getting lung cancer and why?

Conclusion

Thank participant for time

Is there anything else you would like to add that we might have missed out?

APPENDIX 5. Sub-Study 6: Is there a difference in the emotional, cognitive and behavioural response to a positive earlyCDT test if pulmonary nodules are present on a chest computed tomography compared to a normal chest computed tomography?

Researchers

Dr. Marcia Clark (Primary Care, University of Nottingham)

Prof. Denise Kendrick (Primary Care, University of Nottingham)

Prof. Kavita Vedhara (Primary Care, University of Nottingham)

Background:

In the United States, it is estimated that every year hundreds of thousands of pulmonary nodules are detected following computed tomography (CT) examination of the chest [1]. With the increasing use of CT scanning for high risk individuals, the often incidental finding of pulmonary nodules is only going to rise. Indeed, it is thought that pulmonary nodules are detected in 20-50 % of individuals who undergo CT screening [2]. In the United States, the National Lung Screening Trial showed that the incidence of pulmonary nodules was 25.9 % in participants with a pack year history of at least 30 years [3]. Whilst the vast majority of pulmonary nodules are benign, the National Lung Screening Research Team found that in 1.1 % of cases they were cancerous [3].

The Early Cancer Detection Test - Lung Cancer Scotland Study (ECLS study), is currently assessing the effectiveness of using a blood test, which detects autoantibodies to tumour antigens (EarlyCDT-Lung test), in high risk individuals. Those with a positive EarlyCDT-Lung test undergo 6-monthly serial CT scans of their chest. Since it is only those with a positive test that have a subsequent CT scan, this test will potentially reduce the number of high risk individuals who undergo CT scanning. Despite this it is probable that a significant number of individuals will be found have to incidental pulmonary nodules following their CT scan.

Previous studies, although limited, have found that a diagnosis of pulmonary nodules can have a negative impact [4-7]. Slatore and colleagues assessed the psychosocial effect that an incidental finding of pulmonary nodules had on a group of veterans from Portland, Oregon [4, 5]. They employed qualitative interview methods and found that the presence of pulmonary nodules was associated with distress [4, 5]. Although this distress decreased with time, some veterans were noted to have increased levels at the time of their follow-up CT scans (1 and 2 years after their original diagnosis) [4, 5]. Their findings are supported by work completed by Weiner, who found through the use of focus groups (participants were undergoing pulmonary nodule surveillance with a median time since diagnosis of 10 months), that a diagnosis of pulmonary nodules results in frustration and fear [7]. The participants' fear was related to their perceived risk of cancer and whilst in some this fear diminished with time, there were participants (particularly those with a family history of cancer) who continued to experience a negative emotional response [7].

To the investigators best knowledge, there have not been any studies investigating the emotional, cognitive and behavioural effect of a diagnosis of pulmonary nodules following a CT scan within a United Kingdom population. In addition, the majority of studies are qualitative in nature. This study aims to address this knowledge gap through the use of validated quantitative health outcome measures. Based on previous studies, the investigators hypothesise that participants of the ECLS study who are diagnosed with pulmonary nodules, will have adverse emotional, cognitive and behavioural responses compared to those who have a normal CT scan.

Objective:

To determine whether the short and long term emotional, cognitive and behavioural response to having a positive early CDT test differs between participants diagnosed with pulmonary nodules on their chest CT and those that have a normal chest CT.

Methods:

Participants and Procedure

Study participants will be taken from the EarlyCDT-positive group who participated in the emotional and behavioural outcomes study and completed the baseline questionnaire and at least one follow-up questionnaire at one, three or six months post recruitment. It is estimated that approximately 150 participants in this group have had a chest CT that shows the presence of pulmonary nodules that are 8 mm or less in diameter (coded 1b on the ECLS Radiology Schema). A comparison group will comprise the remaining participants in the EarlyCDT-positive group with a normal chest CT (coded 1a on the ECLS Radiology Schema). Data collected from the questionnaire study will be compared between the pulmonary nodule and normal chest CT groups at baseline, 1, 3 and 6 months. The emotional outcomes of interest will be EQ5D, positive and negative affect schedule (PANAS), health anxiety subscale of health orientation scale (HOS), lung cancer worry scale (LCWS) and impact of events scale. The revised illness perception questionnaire - lung cancer (IPQ-LC) and lung cancer risk perception will be used to determine the cognitive response. Differences in behavioural response will be assessed using smoking behaviour and health utilisation data.

Analysis

Continuous data will be described using means and standard deviations or medians and interquartile ranges, depending on the distribution. Box and whisker plots will also be used to graphically display the differences between the pulmonary nodule and normal chest CT groups. Histograms will be used to illustrate discrete data.

Baseline characteristics of participants with and without nodules will be compared using 2-sample t-tests or Mann Whitney U tests as appropriate for continuous data and chi-squared tests for categorical

data. Outcomes at 1, 3 and 6 months will be compared between participants with and without nodules using multilevel linear (for continuous outcomes) or logistic (for binary outcomes) regression.

Two-level models will be used with observations at level one and participants at level two. Analyses will:

(a) Adjust for baseline values of outcome variables.

(b) Adjust for baseline values of outcome variables, plus variables used in the minimisation for the ECLS trial (age, sex, smoking history, socio-economic status and practice). If appropriate, practice will be adjusted for as a random effect rather than as a fixed effect.

(c) Adjust for baseline values of outcome variables, plus minimisation variables, plus a prior defined confounder (educational level, family history of lung cancer, taking antidepressants) and variable imbalances at baseline.

All data will be stored in compliance with the Data Protection Act 1998 and in accordance with University of Dundee, Health Informatics and TCTU Standard Operating Procedures and the University of Nottingham Code of Research Conduct and Research Ethics (non-identifiable study management information only, dates of interviews etc.). The researchers will be responsible for maintaining all documents concerning the study. The extracted data will be stored on a University of Nottingham on a password protected computer in a locked office at Queens Medical Centre and will only be accessible by the research team (e.g., the researchers and research supervisors).

Each participant will be assigned a unique study identity code number for use on their interview transcript. Transcripts and signed consent forms will be treated as confidential documents and held securely in accordance with regulations. Each transcript document will be password protected and will be stored on a password protected file on University of Nottingham server. Only members of the research team (e.g., the researchers and the research supervisors) will have access to interview

transcripts. Audio recordings will be stored on an audio recorder in a locked filing cabinet. Once audio recordings have been transcribed, the recording will be deleted off the audio recorder. Signed consent forms will be kept in the study ISF in a locked filing cabinet in a locked office at Queens Medical Centre. In line with the University of Nottingham Code of Research Conduct and Research Ethics, data will be stored for seven years from the date of any publication that is based upon them.

Data protection

All members of the research team will adhere to the Data Protection Act, 1998. Study documents will contain only the minimum required information for the purposes of the study.

The results of this study will be disseminated at conferences and published in peer-reviewed journals. A summary of our findings and recommendations will be produced for dissemination to clinicians, professional bodies and the UK National Screening Committee. A plain English summary of our findings will be published on the ECLS study website for participants to access. This will also be made available to relevant lung cancer charities.

References:

[1] Ost, D., Fein, A.M. and Feinsilver, S.H., *The Solitary Pulmonary Nodule*, The New England Journal of Medicine, 348, 2535-2542, 2003.

[2] Callister, M.E.J., Baldwin, D.R., Akram, A.R., Barnard, S., Cane, P., Draffan, J., Franks, K., Gleeson, F., Graham, R., Malhotra, P., Prokop, M., Rodger, K., Subesinghe, M., Waller, D. and Woolhouse, I., British Thoracic Society Pulmonary Nodule Guideline Development Group, *British Thoracic Society guidelines for the investigation and management of pulmonary nodule*, Thorax, 70, ii1-ii54, 2015.

[3] Aberle, D.R., Adams, A.M., Berg, C.D., *et al.,* National Lung Screening Trial Research Team, *Reduced lung-cancer mortality with low-dose computed tomographic screening*, New England Journal of Medicine, 365, 395-409, 2011.

[4] Slatore, C.G., Pres, N., Au, D.H., Curtis, J.R., Wiener, R.S. and Ganzini, L., *What the heck is a "nodule"? A qualitative study of veterans with pulmonary nodules*, Annals of the American Thoracic Society, 10, 330-335, 2013.

[5] Sullivan, D.R., Golden, S.E., Ganzini, L., Hansen, L. And Slatore, C.G., *'I still don't know diddly': a longitudinal qualitative study of patients' knowledge and distress while undergoing evaluation of incidental pulmonary nodules*, Primary Care Respiratory Medicine, 25, 2015.

[6] Lemonnier, I., Baumann, C., Jolly, D., Arveux, P., Woronoff-Lemsi, M.C., Velten, M. and Guillemin, F., *Solitary pulmonary nodules: consequences for patient quality of life*, Quality of Life Research, 20, 101-109, 2011.

[7] Weiner, R.S., Gould, M.K., Woloshin, S., Schwartz, L.M. and Clark, J.A., *'The thing is not knowing': patients' perspectives on surveillance of an indeterminate pulmonary nodule*, Health Expectations, 18, 355-365, 2012.

APPENDIX 6. Living with lung nodules: what information would patients find helpful?

Investigators:

Dr. Marcia Clark (Primary Care, University of Nottingham)

Prof. Denise Kendrick (Primary Care, University of Nottingham)

Prof. Kavita Vedhara (Primary Care, University of Nottingham)

Prof. Roshan das Nair (Rehabilitation & Ageing, University of Nottingham)

Prof. John Robertson (Director of Centre of Excellence for Autoimmunity on Cancer, University of Nottingham)

Background:

Pulmonary nodules are widely defined as round lesions within the lung that are less than 3 cm in diameter and entirely surrounded by normal lung tissue [1, 2]. It is estimated in the United States, that every year at least 150,000 individuals with pulmonary nodules are detected following computed tomography (CT) examination of the chest [3]. Pulmonary nodules are found in 20-50 % of high risk individuals who undergo CT screening [1]. It is anticipated that the UK incidence of pulmonary nodules is going to increase in light of the possibility of lung cancer screening for high risk individuals.

The Early Cancer Detection Test - Lung Cancer Scotland Study (ECLS study), is currently assessing the effectiveness of using a blood test (EarlyCDT-Lung test), which detects autoantibodies to tumour antigens in high risk individuals. Those with a positive EarlyCDT-Lung test will then undergo 6-monthly serial CT scans of their chest for two years. The resultant CT scans are reviewed and coded according to the ECLS Radiology Schema. Participants of the study who are found to have pulmonary nodules less than 8 mm in diameter (coded 1b) are sent a letter informing them of this result and are advised to contact the study team should they wish to discuss their result further.

Previous studies, although limited, have found that a diagnosis of pulmonary nodules can have a negative impact, causing distress, frustration, fear and reduced health-related quality of life [4-7]. A systematic review by Hagerty showed that amongst cancer patients the manner and quality in which their diagnoses are communicated impacts on the patient's subsequent emotional and behavioural response [8]. For physicians, communicating an incidental finding of pulmonary nodules to individuals can also be challenging. This is especially in light of the fact that a study by Golden and colleagues found that primary care physicians in America felt that they did not have adequate information from their respiratory colleagues to communicate the incidental finding effectively to their patients [9]. It has been shown for breast cancer screening that women who receive their results by letter have a lower level of understanding and satisfaction than those who receive their result in person or over the telephone [10]. Despite this, screening results of screening programmes (e.g. breast, cervical, colorectal cancer screening) in the United Kingdom are communicated in writing in a manner similar to the ECLS study. It is therefore imperative that the results letter sent provides adequate information in order to minimise any negative impact that a diagnosis of pulmonary nodules could potentially have.

The aim of the study is to explore the ECLS study participant response following receipt of a letter informing them that their CT scan showed a pulmonary nodule. The letter currently used in the trial is based on that used in routine clinical care. It is important to explore patient's experiences following receipt of the letter to inform any roll out of lung cancer screening as a national programme. Through the use of focus groups the existing letter will be reviewed and, if deemed appropriate, modifications will be made with a view to improving how these test results are communicated.

Objectives:

1) Develop an understanding of the current participant experience following receipt of the letter informing them that they have a diagnosis of pulmonary nodules. This will be achieved by answering the following questions:
    a. What was the participant's emotional response on receipt of the letter?
    b. What was the participant's initial and subsequent behavioural response to receipt of the letter?
    c. What is the participant's understanding of pulmonary nodules and their relationship to lung cancer?
2) How can the provision of information following a diagnosis of pulmonary nodules be optimised?
3) What is the participant response to the modified information letter?

Methods:

The objectives of this study will be achieved through the use of four different focus groups. Groups 1 and 2 will focus on the current experiences of the ECLS trial participants and will seek to address objectives 1 and 2. Groups 3 and 4 will seek to address the third objective. Each focus group will have a maximum of 8 participants; this number has been chosen to balance the ability of managing the group with the production of high quality data [11]. It is anticipated through the use of focus groups that data will be generated that reflects a variety of opinions, whilst respecting what could potentially be a sensitive topic [12, 13].

Focus Group Participants

Eligible participants will be:

- Recruited to the ECLS trial and given consent to be contacted for future research.

- In the EarlyCDT-positive group and have been informed by letter that they have lung nodules on their study CT. A range of participants will be selected with different times since diagnosis of their lung nodules.
- Able and willing to give informed consent for participation in the study.

Focus Group Recruitment

It is intended that a diverse range of participants are recruited with different demographic backgrounds in order to obtain a good variation of views and experiences. Maximum variation sampling will therefore be employed with the demographics of the participants in focus group 1 analysed prior to recruiting the second. This process will be repeated for focus groups 3 and 4. The demographic factors considered relevant to this study include age, gender, smoking history, GP practice location, level of deprivation (from the Scottish Index of Multiple Deprivation), educational level and time since diagnosis of a pulmonary nodule.

Participants that fit the eligibility criteria will be identified from the ECLS study databases (baseline questionnaire and CT result). Letters will be sent to eligible participants who have previously agreed to be contacted for future research, with a participant information leaflet, consent form and a response slip indicating their interest and availability for participating in the study. On receipt of their response slip a researcher will contact the participant by telephone, and describe the study to them, answer any questions they may have and ask if they are still happy to participate in the study. If they are, they will be advised of the date, time and venue of the focus group. In addition, they will be asked demographic information including their marital status, work situation and smoking history. Non-responders will be sent a reminder letter 14 days after the initial letter. Further letters will be sent to potential participants until an adequate number have been recruited. Participants will be reminded of the focus group one day prior to the date by a phone call from a researcher.

Focus Group Logistics

There will be four focus groups in total. Focus groups 1 and 3 will be conducted in Glasgow. Focus groups 2 and 4 will be conducted in Dundee. Glasgow and Dundee have been chosen as the ECLS study recruited participants from these two locations. It is anticipated that community venues will be used, with each focus group being facilitated by two researchers; one will act as the moderator facilitating the group and the other as observer taking notes. Refreshments will be provided.

Procedure

Prior to the focus groups commencing, written informed consent will be obtained from each participant. A copy will be sent to the participant after the event, a copy to their GP (with consent) and one filed in the ISF. They will be advised that the session will be audio recorded, with a verbatim transcript generated of the discussion held, which will be anonymised. The basis of each focus group will be centred on the relevant objectives as outlined above and structured according to the focus group guide (see below). At the end of each of the focus groups participants will offered reasonable travel expenses and issued as per local policy and procedure and be given a £5 voucher to thank them for their contribution. They will also be advised that on their request they can be sent a plain English summary of the findings of the study which will be documented at time of consent. At any stage of the study participants can request to be withdrawn. Participants do not need to give a reason for this and doing so will not impact on their future care. They will be informed that they can withdraw their data up to 24 hours after the focus group. After this time, the data will have been transcribed and anonymised and therefore, cannot be withdrawn.

It is anticipated that participation in the focus groups will not result in the occurrence of any form of adverse events. However, the researchers are aware that discussion during the focus group may be sensitive and potentially distressing. Should any undue distress occur, participants will be supported

should they wish to withdraw from the focus group and study. They will be advised to seek help from the Principal Investigator, Research Nurse at their site or consult with their general practitioner. Study documentation and digital audio recorders will be security stored in a lockable box/brief case after each focus group prior to transportation and secure storage at the University of Nottingham.

Analysis

Each of the focus groups will be recorded using audio equipment, transcribed verbatim and analysed using the framework method. This involves the researchers familiarising themselves with the transcription, coding the data, developing a working analytical framework, applying this framework and charting the data into the framework matrix [14]. The information obtained from focus groups 1 and 2 will be used to optimise the participant information following a pulmonary nodule diagnosis, with further refinements made following focus groups 3 and 4. Although direct quotes and extracts from the focus groups may be presented in the research outputs, they will be anonymised to ensure that participants cannot be identified through the data. Participants will be assigned pseudonyms to protect their identities.

All data will be stored in compliance with the Data Protection Act 1998 and in accordance the University of Nottingham Code of Research Conduct and Research Ethics (non-identifiable study management information only, dates of interviews etc.). The researchers will be responsible for maintaining all documents concerning the study. The data will be stored on a University of Nottingham on a password protected computer in a locked office at Queens Medical Centre and will only be accessible by the research team (e.g., the researchers and research supervisors).

Each participant will be assigned a unique study identity code number for use on their interview transcript. Transcripts and signed consent forms will be treated as confidential documents and held securely in accordance with regulations. Each transcript document will be password protected and

will be stored on a password protected file on University of Nottingham server. Only members of the research team (e.g., the researchers and the research supervisors) will have access to interview transcripts. Audio recordings will be stored on an audio recorder in a locked filing cabinet. Once audio recordings have been transcribed, the recording will be deleted off the audio recorder. Signed consent forms will be kept in the study ISF in a locked filing cabinet in a locked office at Queens Medical Centre. In line with the University of Nottingham Code of Research Conduct and Research Ethics, data will be stored for seven years from the date of any publication that is based upon them.

The results of this study will be disseminated at conferences and published in peer-reviewed journals. A summary of our findings and recommendations will be produced for dissemination to clinicians, professional bodies and the UK National Screening Committee. A plain English summary of our findings will be published on the ECLS study website for participants to access. This will also be made available to relevant lung cancer charities.

Alternative Data Collection

Should it prove too difficult to organise the focus groups as outlined above, semi-structured interviews will be used as an alternative means of data collection. There will be two different interview types. The first will cover the first and second objectives. The second will cover the third objective. Participants for the interview covering the third objective (what is the participants' response to the modified information letter?) will be sent the modified letter at least one week prior to the interview, to allow time for reading the modified letter. Questions asked in the interview will be the same as those within the focus group guide. Participants will be recruited using the same criteria as that for the focus groups. Written consent will be obtained, with the interviews held face-to-face or over the telephone dependent upon participant preference. Where interviews are conducted by telephone consent forms will be posted to potential participants and interviews will only be conducted once completed forms have been returned. The interview will be audio recorded, with a verbatim transcript

generated of the discussion held, which will be anonymised. Data will be analysed as described above for the focus groups. Interviews will be continued until data saturation is reached.

Focus Group/Interview Guides:

The questions within this guide are designed to act as a participant prompt and as a means of steering participant discussion in order to achieve the objectives of the study. They are intended to facilitate discussion and debate amongst participants, rather than a question and answer session between participant and facilitator.

Prior to commencement of all the focus groups participants will complete both consent and demographic forms. Before starting the recording equipment ground rules will be established, including the need for confidentiality amongst participants. Each focus group will start with an introduction advising the participants that it is their thoughts and opinions that are being sought and that there are no right or wrong answers.

Focus Groups 1 and 2

Following the general introduction the participants will be provided with a copy of the ECLS trial pulmonary results letter.

1) Participant feelings on receipt of the pulmonary nodule results letter
    - What happened when you first received the letter?
    - What was your initial reaction to the letter?
    - What was your understanding of what the letter was trying to inform you of?
    - What was your understanding of the future plan following diagnosis of a nodule?
    - How did you feel about finding out this result in the form of a letter?
2) Participant response following receipt of the pulmonary nodule results letter

- Did you seek any advice or further information after you received the letter? If so how did you do this?
- How do you now feel about having a nodule within your lungs?
- How have your feelings about the nodule changed since you received the letter?
- How often do you think about your lung nodule?
- What do you find most difficult about living with a lung nodule?
- Has the finding of a nodule within your lungs changed your lifestyle? Ask specifically about smoking behaviour if not discussed.

3) Understanding of pulmonary nodules
   - What do you think a lung nodule is?
   - How likely do you think it is that the nodule will become cancer?
   - How often and for how long do you think you will be followed up for as a result of having a lung nodule?
   - Do you think that your nodule is causing you to have symptoms? If so which ones?
   - If you wanted to explain the presence of nodules in your lungs to your family or friends, what would you say to them?

4) Improvement to information provision

   At this stage participants will be given a short explanation of lung nodules.

   - Do you think that the results letter could be improved? If so how? Consider including a definition of a lung nodule, images, the risk of lung cancer, details of a follow-up plan and symptoms that should trigger a visit to their GP.
   - Knowing what you know now, are there things it would have been helpful to know at the time you were told you had a lung nodule?
   - What would be your preferred method of receiving news that you had a lung nodule? Why would you prefer that method? Consider the provision of results in person, over

the telephone, a link to online information or a link to a YouTube video of a physician explaining a diagnosis of lung nodules.

Focus Groups 3 and 4

Participants will be given copies of both the original nodule result letter and the modified nodule result letter and information.

1) Response to modified information provision
   - How would your initial feelings differ if you were to receive the modified letter?
   - How would this letter change what you did after being told you had a lung nodule? How might it affect you looking for information elsewhere? How might it affect where you looked for information. How might it affect the sort of information you looked for? (e.g. about what lung nodules are , risk of cancer, follow-up scans, affect of lifestyle on reducing the chance on progressing to cancer).
   - Has your understanding of lung nodules changed since reading the modified letter?
   - How does the modified nodule result letter compare to the original letter?
   - Are there any other ways the letter could be improved?

All focus groups will close by asking whether there is anything else that the participants would like to share with the group and thanking the participants for their time.

References

[1] Callister, M.E.J., Baldwin, D.R., Akram, A.R., Barnard, S., Cane, P., Draffan, J., Franks, K., Gleeson, F., Graham, R., Malhotra, P., Prokop, M., Rodger, K., Subesinghe, M., Waller, D. and Woolhouse, I., British Thoracic Society Pulmonary Nodule Guideline Development Group, *British Thoracic Society guidelines for the investigation and management of pulmonary nodule*, Thorax, 70, ii1-ii54, 2015.

[2] Folch, E.E. and Mazzone, P.J., *Assessment of solitary pulmonary nodule*, BMJ Best Practice, 2015.

[3] Ost, D., Fein, A.M. and Feinsilver, S.H., *The Solitary Pulmonary Nodule*, The New England Journal of Medicine, 348, 2535-2542, 2003.

[4] Slatore, C.G., Pres, N., Au, D.H., Curtis, J.R., Wiener, R.S. and Ganzini, L., *What the heck is a "nodule"? A qualitative study of veterans with pulmonary nodules*, Annals of the American Thoracic Society, 10, 330-335, 2013.

[5] Sullivan, D.R., Golden, S.E., Ganzini, L., Hansen, L. And Slatore, C.G., *'I still don't know diddly': a longitudinal qualitative study of patients' knowledge and distress while undergoing evaluation of incidental pulmonary nodules*, Primary Care Respiratory Medicine, 25, 2015.

[6] Weiner, R.S., Gould, M.K., Woloshin, S., Schwartz, L.M. and Clark, J.A., *What do you mean, a spot? A qualitative analysis of patients' reactions to discussions with their physicians about pulmonary nodules*, Chest, 143, 672-677, 2013.

[7] Lemonnier, I., Baumann, C., Jolly, D., Arveux, P., Woronoff-Lemsi, M.C., Velten, M. and Guillemin, F., *Solitary pulmonary nodules: consequences for patient quality of life*, Quality of Life Research, 20, 101-109, 2011.

[8] Hagerty, R.G., Butow, P.N., Ellis, P.M., Dimitry, S. and Tattersall, M.H.N., *Communicating prognosis in cancer care: a systematic review of the literature*, Journal of Oncology, 16, 1005-1053, 2005.

[9] Golden, S.E., Weiner, R.S., Sullivan, D., Ganzini, L., Slatore, C., *Primary care providers and a system problem: A qualitative study of clinicians caring for patients with incidental pulmonary nodules*, Chest, 148, 1422-1429, 2015.

[10] Marcus, E.N., Drummond, D. and Dietz, N., *Urban women's preferences for learning of their mammogram result: A qualitative study*, Journal of Cancer Education, 27, 156-164, 2012.

[11] Braun, V. and Clarke, V., *Successful Qualitative Research*, Sage, London, 2013.

[12] Wellings, K., Branigan, P. And Mitchell, K., *Discomfort, discord and discontinuity as data: Using focus groups to research sensitive topics*, Culture, Health & Sexuality, 2, 255-267, 2010.

[13] Jordan, J., Lynch, U., Moutray, M., O'Hagan, M.T., Orr, J., Peake, S. and Power, J., *Using focus groups to research sensitive issues: Insights from group interviews on nursing in the Northern Ireland "troubles",* International Journal of Qualitative Methods, 6, 1-19, 2007.

[14] Gale, N.K., Heath, G., Cameron, E., Rashid, S. and Redwood, S., *Using the framework method for the analysis of qualitative data in multi-disciplinary health research,* BioMed Central Medical Research Methodology, 13, 117-224, 2013.

APPENDIX 7. List of Investigators and Collaborators

**Co-Investigators**
Prof John Robertson<br>University of Nottingham
Prof Kavita Vedhara<br>University of Nottingham
Prof Denise Kendrick<br>University of Nottingham
Prof Herb Sewell<br>University of Nottingham
Dr Alistair Dorward<br>NHS Greater Glasgow & Clyde
Prof Shaun Treweek<br>University of Aberdeen
Prof Frances Mair<br>University of Glasgow
Dr Colin McCowan<br>University of Glasgow
Dr Mike Sproule<br>NHS Greater Glasgow & Clyde
Dr Andrew Briggs<br>University of Glasgow
Prof Cathy Jackson<br>University of St Andrews
Dr Robert Milroy<br>NHS Greater Glasgow & Clyde
Dr Tom Taylor<br>NHS Tayside
Dr Gozde Ozakinci<br>University of St Andrews
Dr Roberta Littleford<br>TCTU, University of Dundee

**Collaborators**
Dr David Brewster<br>Scottish Cancer Registry
**Local Investigators**
Dr William Anderson<br>NHS Tayside
Dr Peter Brown<br>NHS Tayside
Dr Roddy Cameron<br>NHS Tayside
Dr Alan Cook<br>NHS Tayside
Dr Scott Davidson<br>NHS Greater Glasgow & Clyde
Dr Tom Fardon<br>NHS Tayside
Dr Peter Garmany<br>NHS Greater Glasgow & Clyde
Dr Yoris van der Horst<br>NHS Greater Glasgow & Clyde
Dr Ewan Ross<br>NHS Greater Glasgow & Clyde
Dr Joseph Sarvesvaran<br>NHS Greater Glasgow & Clyde
Dr Simon Sheridan<br>NHS Greater Glasgow & Clyde
Dr Robin Smith<br>NHS Tayside
Dr Manish Patel<br>NHS Lanarkshire

Dr Fiona Hogarth<br><br>TCTU, University of Dundee	Dr Cindy Chew<br><br>NHS Lanarkshire
Dr Marcia Clark<br><br>University of Nottingham	Dr Leigh Smart<br><br>NHS Lanarkshire
Dr Jennifer Hancox<br><br>University of Nottingham	

Appendix 2. Statistical Analysis Plan; Version 1.0

TRIAL FULL TITLE	Detection in blood of autoantibodies to tumour antigens as a case-finding method in lung cancer using the EarlyCDT-Lung test
CLINICAL TRIALS GOV ID	NCT01925625
SAP VERSION	1.0
SPONSOR R&D NUMBER	2013ON07
SAP VERSION DATE	16/05/2019
TRIAL STATISTICIAN	Petra Rauchhaus
TRIAL CHIEF INVESTIGATOR	Professor Frank Sullivan
SAP AUTHOR	Petra Rauchhaus

SAP Approvals:

Role	Name	Signature	Date
SAP AUTHOR	Petra Rauchhaus		
LEAD STATISTICIAN	Alex McConachie		
CHIEF INVESTIGATOR	Prof Frank Sullivan		
CO-CHIEF INVESTIGATOR	Dr Stuart Schembri		

Table of Contents

Contents	146
Abbreviations and Definitions	147
1. Introduction	148
2. Study Objectives and Endpoints	149
3. Study Methods	152
4. Sample Size	153
5. General Considerations	156
6. Summary of Data	157
7. Efficacy Analyses	157
8. Safety Analyses	162
9. Reporting Conventions	163
10. Technical Details	163
11. Example Tables	163
Appendix 1: List of Sub-Studies	164

Abbreviations and Definitions

AAB	Autoantibody
AE	Adverse event
CRF	Care Report Form
CI	Chief Investigator
CNORIS	clinical negligence and other risks scheme
CT scan	Computerised Tomography Scan
CXR	Chest X-Ray
eCRF	Electronic case report form
EarlyCDT – Lung Test	Early Cancer Detection Test- Lung Test
ECLS Study	Early Cancer Detection Test – Lung Cancer Scotland Study
GG&C	Greater Glasgow & Clyde
GCP	Good Clinical Practice
HEAP	Health Economics Analysis Plan
HIC	Health Informatics Centre
ICF	Informed Consent Form
ISF	Investigator Site File
MAR	Missing at random
SCR	Scottish Cancer Register
SIMD	Scottish Index of Multiple Deprivation
SMR	Scottish Morbidity Record
SOP	Standard Operating Procedure
TASC	Tayside Medical Science Centre
TCTU	Tayside Clinical Trials Unit
TAA	Tumour Derived/Associated Antigens
TMF	Trial Master File
TMN	Tumour, Node, Metastases

1. Introduction

Preface

The EarlyCDT-Lung Test is an early detection test designed to assist in lung cancer risk assessment and detection in the earliest stages of the disease. Survival rates are much higher when cancer is diagnosed early but because lung cancer is often diagnosed symptomatically, most cases are discovered after the disease has spread. In these cases, the 5-year survival rate is less than 10%. By testing patients who are at a high risk for developing lung cancer before symptoms appear, the EarlyCDT-Lung test could help diagnose lung cancer sooner, when treatment options are more likely to be successful. The EarlyCDT-Lung test detects autoantibodies, which are a patient’s immune response to antigens produced by solid-tumor cells. Because these autoantibodies are produced by healthy individuals at lower levels, the EarlyCDT-Lung test enables physicians to identify those patients producing autoantibodies at higher levels and who are at an increased lung cancer risk or who are already in the early stages of lung cancer.

Purpose of the analyses

The analyses proposed in this SAP will be part of the final study report and assess the outcomes described below.

2. Study Objectives and Endpoints

Study Objectives

Primary Objective

To assess the effectiveness of EarlyCDT-Lung test in reducing the incidence of patients with late-stage lung cancer (defined as grade 3, 4 or undefined) at first diagnosis compared with standard practice.

Secondary Objectives

1) to assess the effectiveness of EarlyCDT-Lung test in improving the diagnosis of early-stage lung cancers;
2) to undertake a cost-effectiveness analysis of EarlyCDT-Lung test as a primary screening method compared to standard clinical practice;
3a) to compare lung-cancer mortality, all-cause mortality and cancer-specific mortality in high-risk groups provided with EarlyCDT-Lung test, compared with standard practice;
3b) to compare long-term future mortality in high-risk groups provided with EarlyCDT-Lung test, compared with standard practice;
4) to obtain refined estimates of the sensitivity, specificity, positive predictive value and negative predictive value of EarlyCDT-Lung test;
5) to assess behavioural outcomes including smoking, psychological outcomes including cancer worry, anxiety, depression, distress specific to clinical investigations;
6) to assess the effectiveness of EarlyCDT-Lung test on other clinical outcomes as CVD, COPD, other cancers, hospital stays and outcomes identified though SMR linkage
7) to assess uptake of subsequent investigations such as CXR, CT, bronchoscopy and biopsies.

Endpoints

Primary Outcome

The difference, at 24 months after randomisation, between the number[1] of patients with stage 3, 4 or unclassified lung cancer at diagnosis in the intervention arm, and those in the control arm;

Secondary Outcomes

1) The numbers, at 24 months after randomisation, in the different stages at diagnosis (3/ 4/ U/ other) in the intervention arm and the control arm;
2) The difference, after 2 years, in the costs and outcomes between the intervention arm and the control arm; cost-effectiveness of the intervention compared to standard clinical practice;
3a) The estimates, after 2 years, of lung cancer mortality, all-cause mortality and cancer-specific mortality rates in the intervention arm and in the control arm; assessment of significance of differences;
3b) The estimates, after 5 years and 10 years of long-term mortality rates in the intervention arm and in the control arm; assessment of significance of differences;
4) The estimates, after 2 years of (i) the number of patients with stage 3, 4 or unclassified lung cancer at diagnosis in the EarlyCDT-Lung test-positive group and those in the EarlyCDT-Lung test-negative group and (ii) stage at diagnosis in the EarlyCDT-Lung test-positive and EarlyCDT-Lung test-negative group;
5) The scores at baseline, and follow-up on in a survey administered prior to treatment allocation, including EQ5D, Positive and Negative Affect Schedule (PANAS), Revised Illness Perception Questionnaire – Lung Cancer (IPQ-LC), Lung cancer risk perception, Health anxiety subscale of

[1] The Protocol contains Primary Outcome inconsistencies. For clarity and consistency the SAP will adopt the clinicaltrials.gov published text – incidence and number; however incidence, number and rate will be reported.

Health Orientation Scale (HOS) and the Lung Cancer Worry Scale (LCWS), Medication, smoking behaviour, demographic details. Follow-up questionnaires include same items, plus Impact of Events Scale (intervention group only), healthcare utilisation and dates and results of follow-up investigations for lung cancer (test positive group only). The HADS is not included in follow- up questionnaires. Follow-up questionnaires are EQ-5D, cancer worry, positive and negative mood, smoking behaviour including cessation intentions and attempts; scores in additional questionnaires administered at between 1 and 24 months to subsets of the control arm and intervention arm; (all participants in the EarlyCDT-positive group will be approached with the recruitment aim of 300 from this group collected at 1,3,6,12,18 and 24 months. The EarlyCDT-negative and control groups will be recruited at the same rate as the EarlyCDT-positive group with the recruitment aim of 300 from each group collected at 1,3,6 and 12 months).

6) The incidence of other clinical outcomes such as CVD, COPD, other cancers, hospital stays, identified through SMR linkage, measured at 24 months, 5 and 10 years in the intervention arm and in the control arm; assessment of significance of differences;
7) The numbers in Tayside group (EarlyCDT-Lung test-positive, EarlyCDT-Lung test-negative, control) undertaking subsequent investigations such as CXR, CT, bronchoscopy and biopsies.

3. Study Methods

General Study Design and Plan

This trial is a randomised controlled parallel-arm trial involving 12,000 participants recruited through primary care and community based recruitment strategies in Scotland.

Initially ten thousand participants from general practices in the most deprived quintile of the population Scotland (as measured by the quintiles of the Scottish Index of Multiple Deprivation (SIMD) 2012 - version 2) were recruited. In the second phase of recruitment, following from a sample size recalculation, an additional 2,000 participants from NHS Greater Glasgow and Clyde and NHS Lanarkshire only were invited as recruitment in NHS Tayside was complete.

For a separate sub-study, all participants in the EarlyCDT-positive group were approached with the recruitment aim of 300 from this group collected at 1,3,6,12,18 and 24 months. The EarlyCDT-negative and control groups were recruited at the same rate as the EarlyCDT-positive group with the recruitment aim of 300 from each group collected at 1,3,6 and 12 months. Sub-study analysis was done separately and is not addressed in this SAP (See Appendix 1)

Randomisation

Participants were allocated to intervention or comparison group during the recruitment visit (Visit 1) using a web-based randomisation system; TRuST, provided by Tayside Clinical Trials Unit (TCTU). Set-up of the randomisation system was performed by TCTU staff under the supervision of a TCTU statistician. Randomisation was stratified by site and minimised by age, sex and smoking history.

4. Sample Size

Original sample size calculation

The rate of lung cancer is 187/100,000 per year for patients aged 50-74 in Scotland 2008 (ISD cancer statistics). Deprivation is associated with a higher risk of lung cancer. Those in the most deprived quintile are associated with an increased risk of 1.8 times compared to the middle quintile of deprivation (ISD cancer statistics). This gives an estimated annual lung cancer rate of 336/100,000 among the practices taking part in the study. A high risk group within this population will be selected using similar entry criteria as the Mayo screening study which had a 2% prevalence rate of lung cancer and a further 2% incidence rate over the following 5 years (Swensson 2005). The baseline rate of late stage presentation for the particular high risk population envisaged in this study is uncertain, as is the size of the reduction in late stage presentation likely to be achieved through use of EarlyCDT-Lung. Using an estimated late stage presentation rate of 1,200/100,000 per year in the control group i.e. 2.4% over the two-year follow-up period, we require 85% power at 5% significance (two-sided) to detect an estimated reduction of 35% in presentation rate in the test group, i.e. as low as 780/100,000 per year or 1.56% over the two-year follow-up period. This corresponds to an estimated event rate over the two years of follow-up of 120 events in the control group and 78 events in the test group and implies a required sample size of n=5,000 per group i.e. 10,000 altogether.

The anticipated 35% reduction in event rate between the control group and the test group is justified by current estimates of the capability of the EarlyCDT-Lung test to identify cases (41% sensitivity, 93% specificity) together with current estimates of the specificity of CT scanning (67%). The sample size calculations are based upon standard methods for time to event data using the cpower function in R and stpower exponential procedure in Stata and assuming exponential survival.

They were also confirmed using standard approaches for detecting a change in binomial probabilities, and confirmed using approaches to detect a change in Poisson rates (with essentially identical results as loss to follow up is expected to be low).

The study aims for a short recruitment period and so no allowance has been made for accrual. With such an allowance, say to 1 year, the power will increase to 91% to identify a 35% reduction provided the minimum follow up period of 2 years is observed.

Revised sample size calculation

The initial assumptions of the rate of late stage presentation of 1,200/100,000 per year among the study participants was too optimistic and in January to May 2015 investigations were carried out to inform an increase in the sample size. Baseline information on the 8,639 participants recruited to March 2015 (18 months from first randomisation) was used to derive an estimate of lung cancer risk based upon the Spitz Model. A number of variables in this model were not recorded in the study data base and low risk values were used in the risk calculation implying that the risk estimates should be underestimates. This suggested that the with 10,000 participants the rate of lung cancer would be expected to be around 680/100,000 and 540/100,000 for stage T3/T4/Unknown lung cancer using ISD cancer statistics figures of 80% lung cancers in Scotland are late stage. A sensitivity analysis around the missing data assumptions suggests that a late stage rate of around 600/100,000 may not be unreasonable, though is likely to be at the upper limit.

Using an assumption of 600/100,000 for late stage lung cancer and acknowledging that recruitment is over a 2 year period the study has a power of 80% to detect a 35% reduction associated with the use of the EarlyCDT-Lung test to identify cases, provided that analysis takes place after all randomised patients have been followed up for 2 years. While an 80% power is at the lower end of

acceptable powers this is the power level which has been used in a number of lung cancer screening trials.

The power of the study is sensitive to the assumptions about the rate of late stage cancer and the recruitment rate, see Table 2. A power in excess of 90% could only realistically be achieved by recruiting 15,000 patients or by changing the primary endpoint to 3 years post randomisation for all patients. It the recruitment phase extends past 2 years to 2.5 years to recruit 12,000 participants then the power will increase slightly to 83%.

Table 2. Powers for a 35% reduction in the rate of T3/T4/Unknown lung cancer using a log rank test at the 5% significance level for various underlying rates in the control group, total sample sizes, and differing lengths of recruitment periods and follow up periods.

Recruitment		2 Years	2.5 Years	3 Years	2 Years
Follow Up		2 Years	2 Years	2 Years	3 Years
Rate T3T4/Unk	Sample Size				
500	10000	0.649	0.683	0.715	0.771
	12000	0.727	0.761	0.791	0.841
	15000	0.818	0.847	0.872	0.911
600	10000	0.727	0.761	0.791	0.841
	12000	0.802	0.832	0.858	0.9
	15000	0.881	0.905	0.924	0.952
700	10000	0.791	0.822	0.848	0.892
	12000	0.859	0.884	0.906	0.938
	15000	0.924	0.942	0.956	0.975

5. General Considerations

Timing of Analyses

The analysis for the primary outcome and any outcomes for 2-year follow-up will be performed using data up to 2 years after the last patient randomised, after all data have been entered and the clinical database has been locked.

Scotland-wide follow-up data will be requested from eData Research & Innovation Service (eDRIS), to allow derivation of study outcomes.

Analysis for the 5- and 10-year outcomes will be performed using data up to 5 and 10 years respectively after the last patient randomised.

Analysis Populations

The analysis population will be all available subjects on an intention-to-treat basis for the outcome measures.

Missing Data

The extent of missing data will be examined and multiple imputation will be implemented for missing baseline data to provide robust results, assuming data are missing at random (MAR). It is assumed that there will be no missing data for randomised group as the patients are randomised centrally. It is likewise assumed that there are no missing data in the primary outcome as the data is extracted from Scotland-wide data.

Multiple imputation will be performed using a Markov chain Monte Carlo method with multiple chains over 1000 imputations.

6. Summary of Study Data

All continuous variables will be summarised using the following descriptive statistics: n (non-missing sample size), number of missing records, mean, standard deviation, median, maximum and minimum. The frequency and percentages (based on the non-missing sample size) of observed levels will be reported for all categorical measures. Number of missing records will also be reported.

In general, all data will be listed, sorted by subject and treatment and where appropriate by visit number within subject.

All summary tables will be structured with a column for each treatment in the order (Positive assessment, Negative assessment, Control) and an additional column for the total population relevant to that table/treatment, including any missing observations.

Demographic and Baseline Variables

Baseline characteristics for patients are: GP Practice, age, gender, current smoking status, Pack year history, marital status, employment status, housing status, SIMD at recruitment and ethnicity.

7. Efficacy Analyses

Characteristics of participants will be compared informally between treatment arms at baseline. Cox proportional hazards models will be used to estimate the hazard ratio of the rate of late stage lung cancer in the intervention arm compared to the control arm. Participants who are lost to follow up will be censored. The models will adjust for age, gender, smoking history, and practice. Random cluster effects will be included rather than fixed effects for practices. A similar methodology will be used for the secondary outcomes of comparisons of mortality rates (secondary outcomes 3a and 3b). A subsequent analysis will compare the outcomes of those with EarlyCDT positive in comparison to those in the intervention group with EarlyCDT negative (primary contrast for this analysis) and

those in the control group (secondary analysis 1). Comparisons of proportions (secondary analyses 1 and 4) will be carried out using chi square tests. Fisher's Exact test will be used if the numbers of events are small.

Poisson regression models, adjusting for age, gender, smoking history, practice and follow up time if necessary, will be used to investigate the other clinical measures (secondary outcomes 6 and 7).

Primary Efficacy Analysis

The primary outcome is to assess the difference, up to 24 months after randomisation, in the incidence of stage 3, 4 or unclassified lung cancer at diagnosis, between the intervention arm, and the control arm. The data will be taken from the SMR06 Scottish cancer registry data. The outcome variable is the first occurrence (as defined by the field "DATE OF INCIDENCE") of the field "SITE ICD10" and include all diagnosis starting with C33 and C34. Where more than one tumour is present at diagnosis, the most advanced tumour will be used for classification of disease.

To determine staging, the fields "CLINICAL T, N, M" and "PATHOLOGICAL T, N, M" will be used. Where both fields are present, Pathological staging will be used for greater accuracy. Staging is

classified as:

	No	N1	N2	N3
**T1**	IA	IIB	IIIA	IIIB
**T2a**	IB	IIB	IIIA	IIIB
**T2b**	IIA	IIB	IIIA	IIIB
**T3**	IIB	IIIA	IIIB	IIIC
**T4**	IIIA	IIIA	IIIB	IIIC
**M1a**	IVA	IVA	IVA	IVA
**M1b**	IVA	IVA	IVA	IVA
**M1c**	IVB	IVB	IVB	IVB

Mets O. and Smithuis R. Lung - Cancer TNM 8th edition [internet]. 2017 [cited 25 October 2018]. Available from: http://www.radiologyassistant.nl/en/p58ef5eeb172c8/lung-cancer-tnm-8th-edition.html.

Lung Cancer TNM Status was changed during the trial from version 7 to version 8. This change was performed in Tayside on 01/01/2018, in Glasgow and Greater Clyde in January 2017 and Lanarkshire on 01/02/2017. As the TNM status maps to the Lung Cancer staging in the same way for both TNM versions, and patients were recruited on a 1:1 basis, TNM status from the original data will be used to map to Staging.

A Cox proportional hazards model will be used to estimate the hazard ratio of the rate of late stage lung cancer in the intervention arm compared to the control arm. Participants who are lost to follow up will be censored, with lost to follow-up being defined as participants no longer present on the CHI register. The model will adjust for age, gender, smoking history, and practice. Random cluster effects will be included rather than fixed effects for practices.

Secondary Efficacy Analyses

1) numbers, at 24 months after randomisation, in the different stages at diagnosis (3/ 4/ U/ other) in the intervention arm and the control arm. The data will be taken from the SMR06 Scottish cancer registry data with the same criteria as the primary outcome. Comparisons of proportions will be carried out using chi square tests. If the number of diagnoses is small, Fisher's Exact test will be used instead.

2) Outcome 2 (The difference, after 2 years, in the costs and outcomes between the intervention arm and the control arm; cost-effectiveness of the intervention compared to standard clinical practice) will be assessed by the health economics team and is not described in the SAP. For details of the analysis see the Health Economics Analysis Plan (HEAP)

3a) estimates, after 2 years, of lung cancer mortality, all-cause mortality and cancer-specific mortality rates in the intervention arm and in the control arm; assessment of significance of differences. Date and cause of death will be extracted from the NRS death dataset for all-cause mortality. The field to be used is “PRIMARY CAUSE OF DEATH”. For lung cancer mortality, ICD10 codes C33 and C34 will be used. For cancer specific mortality, all ICD codes from C00 to C97 will be used. All cause mortality will use all records and ICD10 codes in this field.

These outcomes will be assessed in the same way as the primary outcome.

3b) estimates, after 5 years and 10 years of long-term future mortality rates in the intervention arm and in the control arm; assessment of significance of differences. These outcomes will be defined and assessed in the same way as outcome 3a.

4) estimates, after 2 years of (i) the number of patients with stage 3, 4 or unclassified lung cancer at diagnosis in the EarlyCDT-Lung test-positive group and those in the EarlyCDT-Lung test-negative group and (ii) stage at diagnosis in the EarlyCDT-Lung test-positive and EarlyCDT-Lung test-negative group. These outcomes will be assessed as described in secondary outcome 1.

5) For analysis of outcome 5 see section 4.2.2.

6) The incidence of other clinical outcomes such as CVD, COPD, other cancers, hospital stays, identified through SMR linkage, measured at 24 months, 5 and 10 years in the intervention arm and in the control arm; assessment of significance of differences.

Data will be extracted from the Scottish dataset SMR01 (Inpatient visits). The date of admission, the main reason for admission and up to 3 additional reasons will be extracted.

In the primary analysis, only the field "MAIN CONDITION" will be used, in a secondary analysis, all 4 fields will be used. The following ICD10 codes will be extracted: CVD will be defined as all codes from I20 to I25. COPD is defined as all ICD10 codes from J40 to J44. Other cancers will include all ICD10 codes from C00 to C97. No other conditions will be assessed. Hospital stays will be extracted from SMR01 field "LENGTH OF STAY" and used in the economic analysis.

This outcome will be assessed using Poisson regression models, adjusting for age, gender, smoking history, practice and follow up time if necessary.

7) numbers in Tayside group (EarlyCDT-Lung test-positive, EarlyCDT-Lung test-negative, control) undertaking the following subsequent investigations: CXR, CT, bronchoscopy .

Examinations are not part of the public datasets. For Tayside patients, HIC (Health Informatics Centre) will provide a dataset including all examinations of CXR, CT and bronchoscopy and biopsies. No data will be available for participants from the other health Boards.

8. Safety Analyses

Adverse Events

Adverse events were only collected in the positive group and relate to clinical investigations performed in this group. All other participants were referred to standard clinical practice and had no additional parameters assessed.

Adverse events (AE) will be coded with MedDRA 18.1. Where more than one diagnosis is present in the AE description, the AE will be split with all the descriptors kept the same for all diagnosis. Adverse events will be reported by primary System Organ Class (SOC) and Preferred Term (PT). Subjects will be counted only once when calculating the incidence of AEs. An overview table will be created counting the number of adverse events by system organ class and preferred term. Descriptors for Adverse events will be tabulated separately as described for categorical variables in section 8. The total number of AEs will be used as basis for tabulation.

9. Reporting Conventions

P-values ≥0.001 will be reported to 3 decimal places; p-values less than 0.001 will be reported as "<0.001". The mean, standard deviation, and any other statistics other than quantiles, will be reported to one decimal place greater than the original data. Quantiles, such as median, or minimum and maximum will use the same number of decimal places as the original data. Estimated parameters, not on the same scale as raw observations (e.g. regression coefficients) will be reported to 3 significant figures.

10. Technical Details

All analysis will be performed using SAS 9.3. All data, analysis programs and output will be kept on the TCTU Server and backed up according to the internal IT SOPs.

Analysis programs will be required to run without errors or warnings. The analysis programs for outcomes will be reviewed by a second statistician, and any irregularities within the programs will be investigated and fixed and date of finalised analysis programs will be signed and recorded.

11. Example tables

Example tables will be created on a subset of 1000 patients on a blinded basis and will be reviewed and signed off by the Chief Investigator. Prior to the final analysis the Trial Steering Committee statistician will receive example tables for information.

Appendix 1: List of Sub studies

Study	Investigator	Group	Sample size	Timepoints
Sub-Study 1. Investigating the experiences of individuals who choose not to have the EarlyCDT-Lung test	Ben Young	Non-Responders	20 Participants	Periodically
Sub-Study 2. How do participants perceive the EarlyCDT-Lung test and what do they understand about their test results?	Laura Bedford (Dr Gozde in St Andrews will analyse this data)	Negative<br>Positive	Positive n= 15<br>Negative n= 15	10 month study period
Sub-Study 3. An examination of changes in smoking behaviour following EarlyCDT-Lung testing	Ben Young	Negative<br>Positive	Positive n= 15<br>Negative n= 15	Over a 12 month period
Sub-Study 4. MRC START in ECLS - This study assessed the potential impact of the presentation of the PIB on recruitment.	Mairie Pithkely	Practice level data only)	All Participants	Recruitment

Sub- Study 5.1 Exploring Public Perceptions of Lung Cancer Screening - Qualitatively investigate why individuals decided not to take part in the ECLS Trial, after showing initial interest	Hannah Scobie	Non-Responders	20 Non-attenders (Glasgow and Lanarkshire)	One interview
Sub- Study 5.2 Exploring Public Perceptions of Lung Cancer Screening - Exploring differences between the people taking part who self refer for the study as opposed to reply to a GP invitation	Hannah Scobie	Control<br>Negative<br>Positive	All participants	
Sub-Study 6. Is there a difference in the emotional, cognitive and behavioural response to a positive EarlyCDT test if pulmonary nodules are present on a chest computed tomography compared to a normal chest computed tomography?	Marcia Clarke	Positive	300 Participants	Baseline, 1, 3 and 6 months
Sub-Study 7. Living with lung nodules: what information would patients find helpful?	Marcia Clarke	Positive	32 Participants	Varying timepoints

Questionnaire Study	Ben Young will analyse behavioural outcomes. Dr. Jennie Hancox will analyse psychological outcomes	Control<br>Negative<br>Positive	All participants	Follow-up questionnaires will be given to participants at the time-points already specified in the research protocol:<br>• 1-2 weeks following ELCD test results (all groups; n = 300 in each group): to examine emotional consequences following receipt of results from the ELCD test.<br>• 3, 6 and 12 months (patients who receive a negative ELCD test result (n = 300) and the control group (n

				= 300)): to examine long-term emotional consequences of receiving a negative ELCD test result or standard care.<br>• 3, 6, 12, 18 and 24 months (patients who receive a positive ELCD test result (n = 300)): to examine the long-term emotional consequences of receiving a positive ELCD test result and the imaging investigations required (chest x-ray and CT scan followed by 6 monthly CT scans for 24 months).

Appendix 3. Within-trial model-based cost effectiveness analysis: assumptions and parameters.

The within trial model utilised a combination of trial data and assumptions extracted from expert knowledge, assumptions made in the model are presented below:

- Lung cancer prevalence was the same in both arms
- Prevalence was calculated as the proportion of participating patients developing lung cancer during the two-year within trial period.
- 6 monthly non-contrast thoracic CT scans in the test positive arm detected all cases of lung cancer in that arm (100% accuracy over two years)
- Trial resource use data for test positive participants were utilised in the model: initial x-ray and non-contrast thoracic CT scans
- Expert opinion was used to estimate the resource use associated with a diagnosis (confirmatory diagnostic) – whether as a result of a suspicious 6 monthly non-contrast thoracic CT scan in the test positive arm or as a result of participants becoming symptomatic or presenting at A&E in the test negative and control arms
- Confirmatory diagnostic resource use was applied to all participants with a diagnosis of lung cancer during the trial follow-up irrespective of randomisation group
- Confirmatory diagnostic tests consisted of: x-ray, contrast CT scan and a bronchoscopy or CT guided biopsy
- The cost for the bronchoscopy/ CT guided biopsy was calculated by applying the average cost of these tests
- The distribution of lung cancer stage is assumed to be conditional on screened detection (i.e. a positive EarlyCDT®-Lung Test result) vs. symptomatic presentation
- Deterministic sensitivity analysis was conducted by varying the cost of the blood test and prevalence of lung cancer, roughly halving and doubling the original parameters
- Trial parameters are presented in Tables 1 and 2.
- Unit costs are presented in Table 3.

Table 1. Model parameters

Parameter	Description
Probabilities:	
% LC	Proportion of patients developing lung cancer within two years from randomisation. The same proportion is assumed for the screening and no screening arms.
Sens	Sensitivity of the EarlyCDT-Lung test. Computed as the ratio of the number of patients diagnosed with lung cancer and positive test result over the total number of patients diagnosed with lung cancer.
Spec	Specificity of the EarlyCDT-Lung test. Computed as the ratio of the number of patients without lung cancer and negative test result over the total number of patients without lung cancer.
% Late LC	Proportion of LCs diagnosed at late stage (III, IV or U). Estimated separately for true positive patients in the screening arm, on the one

	hand, and false negative in the screening arm jointly with patients in the no-screening arm, on the other hand.
% Early LC	Proportion of LCs diagnosed at early stage (I or II). Estimated separately for true positive patients in the screening arm, on the one hand, and false negative in the screening arm jointly with patients in the no-screening arm, on the other hand.
% CT+	Proportion of patients with positive result after CT scans follow-up screening. Assumed to be 100% (0%) for those with(out) lung cancer.
% CT-	Proportion of patients with negative result after CT scans follow-up screening. Assumed to be 0% (100%) for those with(out) lung cancer.
Costs:	
C_test	Cost of blood test - EarlyCDT®-Lung Test plus cost of 15 minutes of nurse at a GP practice
C_i	Investigation costs: contrast CT scans; Chest X-ray; contrast enhanced staging CT.

Table 2. Parameters values

Parameter	Mean	SE	Distribution
% LC	0.0104		Beta(127, 12081)
Sens	0.3214		Beta(18, 38)
Spec	0.9038		Beta(5451, 580)
% Late LC (True positive)	0.6667		Beta(12, 6)
% Early LC (True positive)	0.3333		Beta(6, 12)
% Late LC (False negative)	0.2970		Beta(30, 71)
% Early LC (False negative)	0.7030		Beta(71, 30)
% Late LC (No screening arm)	0.2970		Beta(30, 71)
% Early LC (No screening arm)	0.7030		Beta(71, 30)
C_test	£105.50		
C_i (True positive)	£1343.54	28.18	Gamma(1282.77, 28.18)
C_i (False negative)	£1,162.77		
C_i (False positive)	£473.26	4.22	Gamma(478.8, 4.23)
C_i (True negative)	£0.00		

C_i (No screening LC)	£1,162.77		
C_i (No screening No LC)	£0.00		

Table 3: Unit costs and sources

Unit of resource	£	Source	Details
Per blood test	95.00	Oncimmune	$124 per kit
Per blood test	10.50	PSSRU 2017/18	15 minutes of nurse time (GP surgery)
Per scan	90.00	NHS reference costs 2017/18	CT of one area, no contrast RD20A in diagnostic imaging tab, outpatient
Per scan	107.00	NHS reference costs 2017/18	CT scan of two areas with contrast (chest and abdomen) RD24Z, diagnostic imaging tab, outpatient
Per x-ray	60.77	ISD costs 2017/18	Other radiology R120X
Bronchoscopy	728.00	NHS reference costs 2017/18	Diagnostic bronch DZ69A day case
CT guided biopsy	1,245.00	NHS reference costs 2017/18	DZ63C major thoracic procedures, 19 years and over, with CC score 0-2

Appendix 4. CONSORT 2010 Checklist for Reporting a Randomised Trial

Section/Topic	Item No	Checklist item	Reported on page No
**Title and abstract**			
	1a	Identification as a randomised trial in the title	P1
	1b	Structured summary of trial design, methods, results, and conclusions (for specific guidance see CONSORT for abstracts)	P2-3
**Introduction**			
Background and objectives	2a	Scientific background and explanation of rationale	P4
	2b	Specific objectives or hypotheses	P4
**Methods**			
Trial design	3a	Description of trial design (such as parallel, factorial) including allocation ratio	P5,<br>App 1 SA23
	3b	Important changes to methods after trial commencement (such as eligibility criteria), with reasons	P7- sample size<br>App1 SA44
Participants	4a	Eligibility criteria for participants	P5,<br>App1 SA27-28
	4b	Settings and locations where the data were collected	P5,<br>App1 SA23, 26, 29
Interventions	5	The interventions for each group with sufficient details to allow replication, including how and when they were actually administered	P5-6,<br>Suppl. Table 1
Outcomes	6a	Completely defined pre-specified primary and secondary outcome measures, including how and when they were assessed	P8,<br>App1 SA21-22
	6b	Any changes to trial outcomes after the trial commenced, with reasons	N/A
Sample size	7a	How sample size was determined	P7,<br>App1 SA43<br>App2 SA153-155
	7b	When applicable, explanation of any interim analyses and stopping guidelines	App2 SA164<br>(sub-studies)
Randomisation:			
Sequence generation	8a	Method used to generate the random allocation sequence	P5-6,<br>App1 SA34<br>App2 SA152

	8b	Type of randomisation; details of any restriction (such as blocking and block size)	P5-6,<br>App1 SA34<br>App2 SA152
Allocation concealment mechanism	9	Mechanism used to implement the random allocation sequence (such as sequentially numbered containers), describing any steps taken to conceal the sequence until interventions were assigned	N/A
Implementation	10	Who generated the random allocation sequence, who enrolled participants, and who assigned participants to interventions	P5,<br>App1 SA34<br>App2 SA152
Blinding	11a	If done, who was blinded after assignment to interventions (for example, participants, care providers, those assessing outcomes) and how	P6-7
	11b	If relevant, description of the similarity of interventions	N/A
Statistical methods	12a	Statistical methods used to compare groups for primary and secondary outcomes	P8,<br>App1 SA46-47<br>App2, SA56-162<br>App3, SA168-170
	12b	Methods for additional analyses, such as subgroup analyses and adjusted analyses	P8,<br>App1 SA60-142<br>(sub-studies)
**Results**			
Participant flow (a diagram is strongly recommended)	13a	For each group, the numbers of participants who were randomly assigned, received intended treatment, and were analysed for the primary outcome	P9,<br>Figure 1
	13b	For each group, losses and exclusions after randomisation, together with reasons	Figure 1
Recruitment	14a	Dates defining the periods of recruitment and follow-up	P5,<br>App1 SA15
	14b	Why the trial ended or was stopped	App1 SA37
Baseline data	15	A table showing baseline demographic and clinical characteristics for each group	Table 1
Numbers analysed	16	For each group, number of participants (denominator) included in each analysis and whether the analysis was by original assigned groups	Table 2<br>Suppl Data Packs 1-3 ST21-30
Outcomes and estimation	17a	For each primary and secondary outcome, results for each group, and the estimated effect size and its precision (such as 95% confidence interval)	P9-11,<br>Table 2<br>Figure 1<br>Figure 2<br>App1 SA43-45

			App2 SA153-155
	17b	For binary outcomes, presentation of both absolute and relative effect sizes is recommended	P9-11
Ancillary analyses	18	Results of any other analyses performed, including subgroup analyses and adjusted analyses, distinguishing pre-specified from exploratory	P9-11
Harms	19	All important harms or unintended effects in each group (for specific guidance see CONSORT for harms)	P11<br>App1 SA38-39, SA49-50
**Discussion**			
Limitations	20	Trial limitations, addressing sources of potential bias, imprecision, and, if relevant, multiplicity of analyses	P12-14
Generalisability	21	Generalisability (external validity, applicability) of the trial findings	P12-14
Interpretation	22	Interpretation consistent with results, balancing benefits and harms, and considering other relevant evidence	P12-14
**Other information**			
Registration	23	Registration number and name of trial registry	P3<br>App1 SA4
Protocol	24	Where the full trial protocol can be accessed, if available	App1 SA4-144
Funding	25	Sources of funding and other support (such as supply of drugs), role of funders	P3<br>App1 SA4
