# Supplementary material for: Earlier diagnosis of lung cancer in a randomised trial of an autoantibody blood test followed by imaging
Source: Eur Respir J. 2021 Jan 14;57(1):2000670. doi: 10.1183/13993003.00670-2020 (PMC7806972; doi:10.1183/13993003.00670-2020)
Supplement: Supplementary file 2 [file ERJ-00670-2020_Supplementary_Tables.pdf]

SUPPLEMENTARY TABLES (ST)

Manuscript Title: Earlier diagnosis of lung cancer in a randomised trial of an autoantibody blood test followed by imaging. The Early Diagnosis of Lung Cancer Scotland (ECLS) Team

Table of Contents:

Supplementary Table S1. Assessment Schedule for Participants in the Intervention Group	ST. 2
Supplementary Table S2. Baseline Questionnaires for Participants	ST. 3
Supplementary Table S3. Estimation of Cost Per Early Stage Lung Cancer Case Detected Per One Thousand Participants Screened	ST. 20
Supplementary Data Pack S1. Primary Outcome Analysis	ST. 21
Supplementary Data Pack S2. Analysis of Primary Outcome for Participants with Records in the Last Two Years of the Trial Period (Subgroup Analysis)	ST. 24
Supplementary Data Pack S3. Secondary Outcome Analyses (Mortality- Lung Cancer, Cancer, All-Cause)	ST. 26

Supplementary Table S1. Assessment Schedule for Participants in the Intervention Group

Procedure	Visit 1	≤ 2 weeks	CT scan follow-up schedule (± 4 weeks)			
			6 months	12 months	18 months	24 months
EarlyCDT-Lung Blood Test	x					
Chest X-Ray		x				
CT Scan *		x	x	x	x	x

* Study CT scan follow-up was carried out only if the EarlyCDT-Blood test result was positive. If a participant entered NHS Clinical Care pathway, then their subsequent study CT scans were cancelled.

Supplementary Table S2. Baseline Questionnaires for Participants

Baseline Questionnaire V (Part A)
Hospital Anxiety and Depression Scale (HADS) (Part B)
Positive and Negative Affect Schedule (PANAS) (Part C)
Views about Lung Cancer (Part D, E)
Views about Health (Part F)
Medical History (G)
Smoking Behaviour (Part H)

ECLS STUDY

Participant Questionnaire

**Cohort ID:**	ECLS – ☐ ☐ ☐ ☐ ☐ ☐
**Participant Initials:**	☐ ☐ ☐
**Please tell us the date you filled in this questionnaire:**	D D – M M – Y Y Y Y
**This participant has been allocated to group:**	**TEST** ☐ **NO TEST** ☐

University of Dundee · The University of Nottingham · University of Glasgow · NHS Scotland · tctu Tayside Clinical Trials Unit

PART A

By placing a tick in one box in each group below, please indicate which statements best describe your own health state today.

Mobility

I have no problems in walking about ☐
I have some problems in walking about ☐
I am confined to bed ☐

Self-Care

I have no problems with self-care ☐
I have some problems washing or dressing myself ☐
I am unable to wash or dress myself ☐

Usual Activities (e.g. work, study, housework, family or leisure activities)

I have no problems with performing my usual activities ☐
I have some problems with performing my usual activities ☐
I am unable to perform my usual activities ☐

Pain/Discomfort

I have no pain or discomfort ☐
I have moderate pain or discomfort ☐
I have extreme pain or discomfort ☐

Anxiety/Depression

I am not anxious or depressed ☐
I am moderately anxious or depressed ☐
I am extremely anxious or depressed ☐

To help people say how good or bad a health state is, we have drawn a scale (rather like a thermometer) on which the best state you can imagine is marked 100 and the worst state you can imagine is marked 0.

We would like you to indicate on this scale how good or bad your own health is today, in your opinion. Please do this by drawing a line from the box below to whichever point on the scale indicates how good or bad your health state is today.

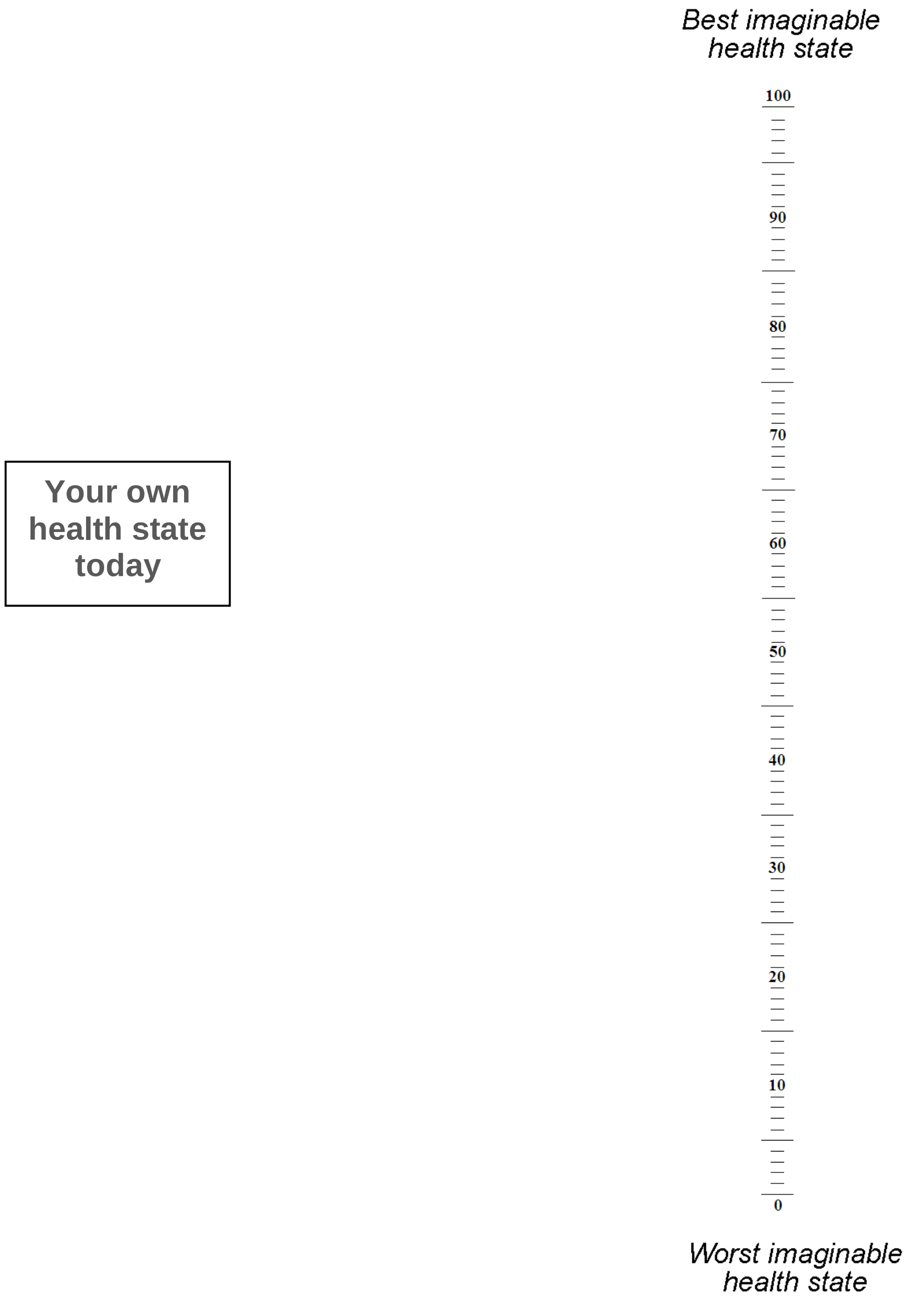

PART B

This questionnaire is designed to help us know how you feel. Read each item and place a tick opposite the reply which comes closest to **how you have been feeling in the past week.**

Don't take too long over your replies: your immediate reaction to each item will probably be more accurate than a long thought-out response. ***Tick only one answer in each section.***

I feel tense or 'wound up':

- Most of the time ☐
- A lot of the time ☐
- Time to time, Occasionally ☐
- Not at all ☐

I still enjoy the things I used to enjoy:

- Definitely as much ☐
- Not quite so much ☐
- Only a little ☐
- Hardly at all ☐

I get a sort of frightened feeling as if something awful is about to happen:

- Very definitely and quite badly ☐
- Yes, but not too badly ☐
- A little, but it doesn't worry me ☐
- Not at all ☐

I can laugh and see the funny side of things:

- As much as I always could ☐
- Not quite so much now ☐
- Definitely not so much now ☐
- Not at all ☐

I feel as if I am slowed down:

- Nearly all the time ☐
- Very often ☐
- Sometimes ☐
- Not at all ☐

I get a sort of frightened feeling like "butterflies" in the stomach:

- Not at all ☐
- Occasionally ☐
- Quite often ☐
- Very often ☐

I have lost interest in my appearance:

- Definitely ☐
- I don't take as much care as I should ☐
- I may not take quite as much care ☐
- I take just as much care as ever ☐

I feel restless as if I have to be on the move:

- Very much indeed ☐
- Quite a lot ☐
- Not very much ☐
- Not at all ☐

Worrying thoughts go through my mind:

- A great deal of the time ☐
- A lot of the time ☐
- From time to time but not too often ☐
- Only occasionally ☐

I look forward with enjoyment to things:

- As much as I ever did ☐
- Rather less than I used to ☐
- Definitely less than I used to ☐
- Hardly at all ☐

I feel cheerful:

- Not at all ☐
- Not often ☐
- Sometimes ☐
- Most of the time ☐

I get sudden feelings of panic:

- Very often indeed ☐
- Quite often ☐
- Not very often ☐
- Not at all ☐

I can sit at ease and feel relaxed:

- Definitely ☐
- Usually ☐
- Not often ☐
- Not at all ☐

I can enjoy a good book or radio or TV programme:

- Often ☐
- Sometimes ☐
- Not often ☐
- Very seldom ☐

PART C

This questionnaire lists a number of words that people often use to describe different feelings and emotions. Read each item and then tick the appropriate answer in the space next to the word. **Indicate the extent you have felt this way over the past week.**

		Very slightly or not at all	A little	Moderately	Quite a bit	Extremely
1)	Interested	☐	☐	☐	☐	☐
2)	Distressed	☐	☐	☐	☐	☐
3)	Excited	☐	☐	☐	☐	☐
4)	Upset	☐	☐	☐	☐	☐
5)	Strong	☐	☐	☐	☐	☐
6)	Guilty	☐	☐	☐	☐	☐
7)	Scared	☐	☐	☐	☐	☐
8)	Hostile	☐	☐	☐	☐	☐
9)	Enthusiastic	☐	☐	☐	☐	☐
10)	Proud	☐	☐	☐	☐	☐
11)	Irritable	☐	☐	☐	☐	☐
12)	Alert	☐	☐	☐	☐	☐
13)	Ashamed	☐	☐	☐	☐	☐
14)	Inspired	☐	☐	☐	☐	☐
15)	Nervous	☐	☐	☐	☐	☐
16)	Determined	☐	☐	☐	☐	☐
17)	Attentive	☐	☐	☐	☐	☐
18)	Jittery	☐	☐	☐	☐	☐
19)	Active	☐	☐	☐	☐	☐
20)	Afraid	☐	☐	☐	☐	☐

From "Development and validation of brief measures of positive and negative affect: The PANAS scales," by D. Watson, L. A. Clark, and A. Tellegen, 1988, Journal of Personality and Social Psychology, 54, 1063-1070. Copyright © 1988 by the American Psychological Association. Reproduced with permission. No further reproduction or distribution is permitted without written permission from the American Psychological Association.

PART D

We are interested in your personal views about **lung cancer**.

Please indicate how much you agree or disagree with the statements below by ticking the box that best describes your views.

	VIEWS ABOUT LUNG CANCER	Strongly agree	Agree	Neutral	Disagree	Strongly disagree
1	**What I do can affect my risk of getting lung cancer**					
2	**When I think about my risk of getting lung cancer I get upset**					
3	**I do not know how likely it is that I might get lung cancer**					
4	**Finding lung cancer early can improve my chances of survival**					
5	**Lung cancer would have a big impact on my life**					
6	**Lung cancer lasts for a long time**					
7	**A blood screening test can accurately detect lung cancer**					
8.	**Please list in rank-order the three most important factors that you believe cause lung cancer.**<br><br>**The most important causes for me are:-**<br><br>1. ……………………………………………………………………………………………<br>2. ……………………………………………………………………………………………<br>3. ……………………………………………………………………………………………					

PART E

1. What are the chances that **you** will develop lung cancer over the next five years? (Please tick one box)

   - Approximately 1 in 1000 (0.1%) ☐
   - Approximately 1 in 500 (0.2%) ☐
   - Approximately 1 in 250 (0.4%) ☐
   - Approximately 1 in 100 (1%) ☐
   - Approximately 1 in 20 (5%) ☐
   - Approximately 1 in 10 (10%), or greater ☐
   - Don't know ☐

2. **Compared to other people of** your age and sex, how likely are **you** to develop lung cancer over the next 5 years? (Please tick one box)

   - A lot less likely than other people ☐
   - Less likely than other people ☐
   - About as likely as other people ☐
   - More likely than other people ☐
   - Much more likely than other people ☐
   - Don't know ☐

PART F

Please read the following statements carefully and decide to what extent it is a **characteristic of you at the moment**. Please **tick one** of the responses to each question.

		Not at all	Slightly	Somewhat	Moderately	Very
1)	I feel anxious when I think about my health	☐	☐	☐	☐	☐
2)	I rarely become discouraged about my health	☐	☐	☐	☐	☐
3)	I am pleased with how well and healthy I feel	☐	☐	☐	☐	☐
4)	I have positive feeling about my health	☐	☐	☐	☐	☐
5)	I feel that I have handled my health very well	☐	☐	☐	☐	☐

Below are four questions asking you about how worried you are about **lung cancer** at the moment. Please **tick one** of the responses to each question.

How worried are you about getting lung cancer someday?

- Not at all worried ☐
- Rarely worried ☐
- Sometimes worried ☐
- Often worried ☐
- Worried almost all the time ☐

How much does your worry affect your mood?

- Not at all ☐
- A little ☐
- Somewhat ☐
- A lot ☐
- Almost all the time ☐

How much does your worry affect your ability to perform your daily activities?

- Not at all ☐
- A little ☐
- Sometimes ☐
- A lot ☐
- Almost all the time ☐

What is your current level of anxiety about the results of future tests/treatments?

- Not at all anxious ☐
- A little anxious ☐
- Somewhat anxious ☐
- Anxious a lot ☐
- Anxious almost all the time ☐

PART G

Below are some questions about medications and OTHER treatments you may be receiving.

1) Have you been prescribed any medications to help you with low mood (e.g., anxiety, depression) in the last 3 months?

Yes ☐ No ☐

If YES, please go to **Question 1a)**
If NO, please go to **Question 2**

1 a) If YES, please state the name of the medication:_______________________

2) Are you currently taking any medications to help you with low mood (e.g., anxiety, depression)?

Yes ☐ No ☐

If YES, please go to **Question 2 a)**
If NO, please go to **Question 3**

2 a) If YES, please state the name of the medication:_______________________

3) Are you currently being investigated for any other conditions?

Yes ☐ No ☐

If YES, please answer **questions 3 a) and 3 b)**
If NO, please go to **PART H**

3 a) If YES, please describe what investigations you are having and why

____

____

____

3 b) How worried are you about these other investigations at the moment?

Not at all worried ☐
A little worried ☐
Quite worried ☐
Very worried ☐

Part H

This study is not about trying to encourage people to stop smoking but we are still interested in your smoking behaviour and your views about smoking.

1) Not counting the last week, have you smoked any cigarettes or tobacco in the last 6 months?

Yes ☐ No ☐

---

2) Have you smoked any cigarettes or tobacco in the last seven days / week?

Yes ☐ No ☐

If YES, please go to **Question 3**
If NO, please go to **PART I**

---

3) How soon after you wake up do you smoke your first cigarette or tobacco? (Tick one box)

After 60 minutes ☐

31 to 60 minutes ☐

6 to 30 minutes ☐

Within 5 minutes ☐

---

4) On average, how many cigarettes do you smoke each day? (Enter a number)

☐

5) Do you have any intention of giving up smoking in the next four weeks? (Tick one box)

Yes, definitely ☐

Yes, probably ☐

Don't know ☐

Probably not ☐

Definitely not ☐

---

6) How sure or confident are you that if you tried, you could give up smoking for good? (Tick one box)

Very certain ☐

Fairly certain ☐

Don't know ☐

Fairly uncertain ☐

Very uncertain ☐

---

7) Which one of these statements do you most strongly agree with? (Tick one box)

I would like to keep on smoking ☐

I don't really want to stop smoking ☐

I don't know whether I want to stop smoking or not ☐

I don't really want to carry on smoking ☐

I would like to stop smoking ☐

8) Which one of these statements do you most strongly agree with? (Tick one box)

I never think about stopping smoking ☐

One day I will need to think about stopping smoking ☐

I should stop smoking but I don't think I'm ready ☐

I am starting to think about how I can smoke less ☐

I am trying to stop smoking ☐

---

9) How strongly do you agree or disagree with the statement "My health will improve if I stop smoking." (Tick one box)

Disagree strongly ☐

Disagree ☐

Don't know ☐

Agree ☐

Agree strongly ☐

---

10) How strongly do you agree or disagree with the statement "People who are important to me want me to stop smoking." (Tick one box)

Disagree strongly ☐

Disagree ☐

Don't know ☐

Agree ☐

Agree strongly ☐

---

11) In the LAST 6 MONTHS have you tried to cut down the number of cigarettes

you smoke?

Yes ☐ No ☐

---

12) In the LAST 6 MONTHS have you tried to stop smoking?

Yes ☐ No ☐

Please continue to the next page.

PART I

Please tell us a little more about yourself. Your answers to these questions will be very helpful to us and will be stored using a unique study code from which you cannot be identified.

1) Date of birth ☐☐ – ☐☐ – ☐☐☐☐

2) Age at which you left full-time education ☐☐

---

3) Marital Status:

Single	☐	Widowed	☐
In a relationship	☐	Separated	☐
Married / in a civil partnership	☐	Divorced	☐

---

4) Which best describes your work situation?

In paid employment ☐

Unable to work due to illness or disability ☐

Unemployed and looking for work ☐

At home and not looking for work ☐

Retired ☐

Other (please describe) ☐ ______________________________

---

5) Do you own or rent your home?

Rented ☐

Owned or mortgaged ☐

Other (Please say what) ☐ ______________________________

6) The total number of people (adults + children) living in my home is:

(Please give a number) ____________________

7) The total number of rooms (excluding halls, landings, toilets and bathrooms) in my home is

(Please give a number) ____________________

8) How many cars or vans are available for use by one or more members of your household?

None ☐

One or more ☐

9) Ethnic Origin:

I would describe my ethnic origin as:		
**Asian or Asian British**	**Mixed**	**Other Ethnic Group**
Bangladeshi ☐	White & Asian ☐	Chinese ☐
Indian ☐	White & Black African ☐	Any other ethnic group (Give details): ☐ ____________________
Pakistani ☐	White & Black Caribbean ☐	I do not wish to disclose my ethnic origin ☐
Any other Asian background ☐	Any other mixed background ☐	
**Black or Black British**	**White**	
African ☐	Scottish ☐	
Caribbean ☐	Other White British ☐	
Any other Black background ☐	Irish ☐	
	Any other White background ☐	

Thank you for completing this questionnaire.

Supplementary Table S3. Estimation of Cost Per Early Stage Lung Cancer Case Detected Per One Thousand Participants Screened.

	**Screening** *Mean (95% CI)*	**No screening** *Mean (95% CI)*	**Difference** *Mean (95% CI)*
**Base case analysis**			
Costs (£)	163,000 (159,000 – 167,000)	12,100 (9990 – 14,400)	151,000 (148,000 – 155,000)
Early stage LCs detected	4.19 (3.02, 5.62)	2.89 (1.91, 4.09)	1.3 (-0.15, 2.77)
ICER			116 000 (54 900, dominated)
**Deterministic sensitivity analysis**			
Prevalence (0.5%)			
Costs (£)	157,000	5810	151,000
Early stage LCs detected	2.01	1.39	0.6
ICER			242,000
Prevalence (2%)			
Costs (£)	175,000	23,300	151,000
Early stage LCs detected	8.06	5.56	2.5
ICER			60,500
EarlyCDT cost (£58.00)			
Costs (£)	116,000	12,100	104,000
Early stage LCs detected	4.19	2.89	1.3
ICER			79 700
EarlyCDT cost (£200.50)			
Costs (£)	258,000	12,100	246,000
Early stage LCs detected	4.19	2.89	1.3
ICER			189,000

Legend: ICER- Incremental cost effectiveness ratio; LC- Lung cancer; CI- Confidence interval.

Note: Dominated is when the intervention arm is more costly and less effective than the control arm

Supplementary Data Pack S1:
Primary Outcome Analysis: Diagnosis of Stage III/IV/Unspecified Lung Cancer Two Years after Randomisation

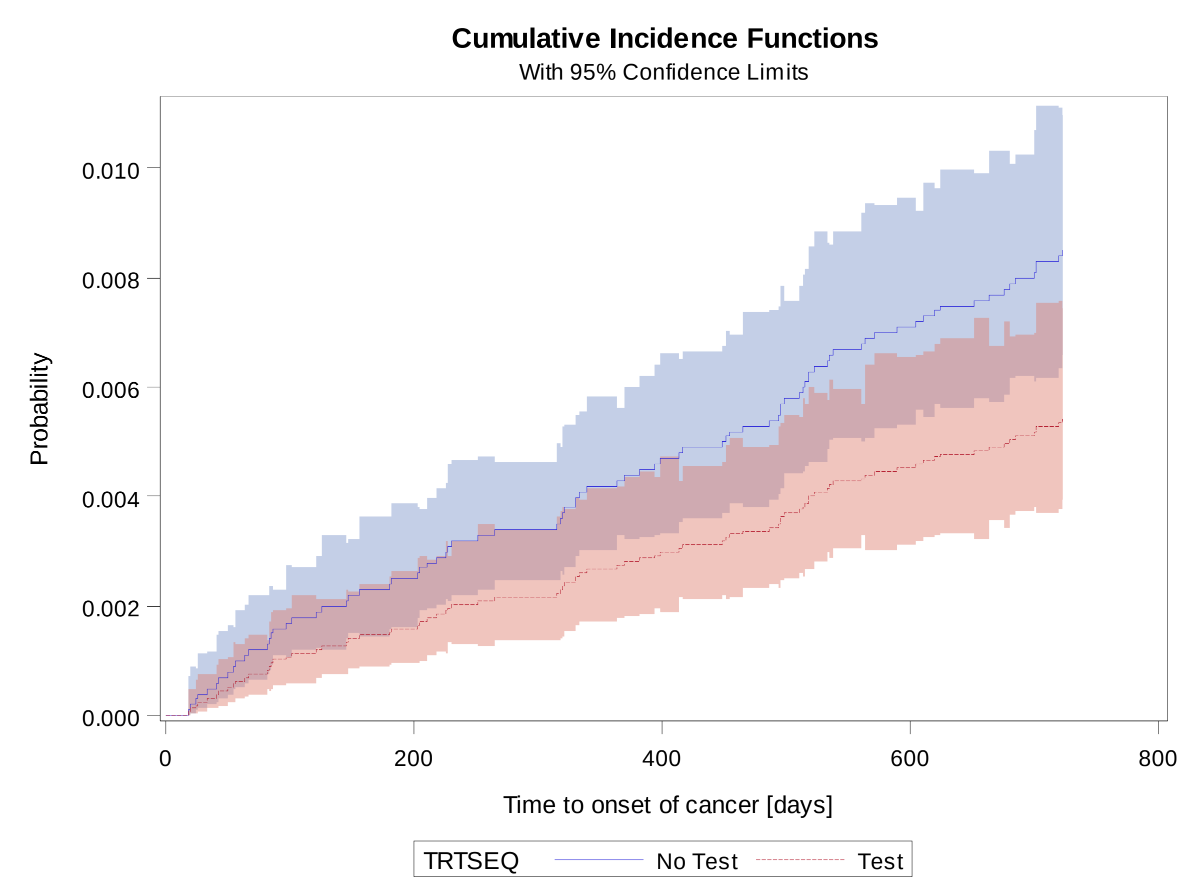

First incidence of late stage lung cancer in the 2 years since randomisation

	No Test		Test		Total	
**Variable**	**N**	**(%)**	**N**	**(%)**	**N**	**(%)**
No	6069	(99.2%)	6054	(99.5%)	12123	(99.3%)
Yes	52	(0.8%)	33	(0.5%)	85	(0.7%)
Total	6121	(100.0%)	6087	(100.0%)	12208	(100.0%)

Lung cancer staging (numeric)

	No Test		Test		Total	
**Variable**	**N**	**(%)**	**N**	**(%)**	**N**	**(%)**
No cancer	6069	(99.2%)	6054	(99.5%)	12123	(99.3%)
Unspecified	7	(0.1%)	0	(0.0%)	7	(0.1%)
Stage 3	17	(0.3%)	15	(0.2%)	32	(0.3%)
Stage 4	28	(0.5%)	18	(0.3%)	46	(0.4%)
Total	6121	(100.0%)	6087	(100.0%)	12208	(100.0%)

Chi-Square Statistics		
**Statistic**	**Value**	**p-Value**
Chi-Square	4.1710	0.0411
Continuity Adj. Chi-Square	3.7382	0.0532
Likelihood Ratio Chi-Square	4.2070	0.0403
Mantel-Haenszel Chi-Square	4.1706	0.0411

Fisher's Exact Test	
Left-sided Pr <= F	0.0263
Right-sided Pr >= F	0.9846
Table Probability (P)	0.0108
Two-sided Pr <= P	0.0495

Analysis of Maximum Likelihood Estimates				
			Type 3 Tests	
**Parameter**	**Parameter Estimate**	**Standard Error**	**Chi-Square**	**p-value**
Group allocation Test	-0.450	0.223	4.0856	0.0432

		Confidence Interval	
**Parameter**	**Hazard Ratio**	**lower**	**upper**
Group allocation Test	0.638	0.412	0.986

Analysis of Maximum Likelihood Estimates				
			Type 3 Tests	
**Parameter**	**Parameter Estimate**	**Standard Error**	**Chi-Square**	**p-value**
Age [years]	0.112	0.018	39.4211	<.0001
Pack year history	0.013	0.003	18.8157	<.0001
Gender Male	0.144	0.226	0.4050	0.5245
Smoking status Ex-smoker	-0.479	0.223	4.6020	0.0319
Group allocation Test	-0.440	0.223	3.9039	0.0482

		Confidence Interval	
**Parameter**	**Hazard Ratio**	**lower**	**upper**
Age [years]	1.119	1.080	1.159
Pack year history	1.013	1.007	1.019
Gender Male	1.155	0.741	1.800
Smoking status Ex-smoker	0.619	0.400	0.959
Group allocation Test	0.644	0.416	0.996

Supplementary Data Pack S2:
Analysis of Primary Outcome for Participants with Records in the Last Two Years of the Trial Period (Post-Hoc Subgroup Analysis)

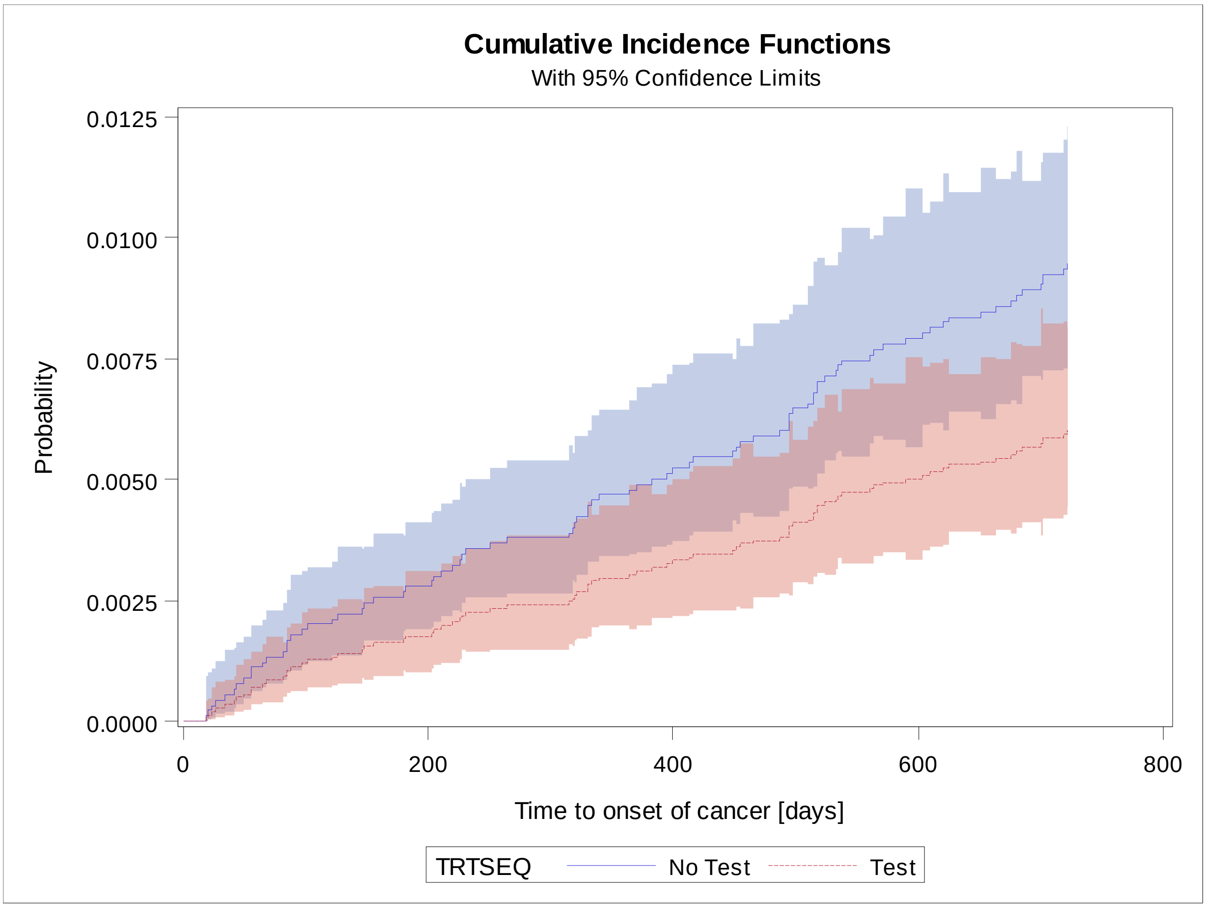

First incidence of late stage lung cancer in the 2 years since randomisation

	No Test		Test		Total	
**Variable**	**N**	**(%)**	**N**	**(%)**	**N**	**(%)**
No	5432	(99.1%)	5456	(99.4%)	10888	(99.2%)
Yes	52	(0.9%)	33	(0.6%)	85	(0.8%)
Total	5484	(100.0%)	5489	(100.0%)	10973	(100.0%)

Lung cancer staging (numeric)

	No Test		Test		Total	
**Variable**	**N**	**(%)**	**N**	**(%)**	**N**	**(%)**
No cancer	5432	(99.1%)	5456	(99.4%)	10888	(99.2%)
Unspecified	7	(0.1%)	0	(0.0%)	7	(0.1%)
Stage 3	17	(0.3%)	15	(0.3%)	32	(0.3%)
Stage 4	28	(0.5%)	18	(0.3%)	46	(0.4%)
Total	5484	(100.0%)	5489	(100.0%)	10973	(100.0%)

Chi-Square Statistics		
**Statistic**	**Value**	**p-Value**
Chi-Square	4.2977	0.0382
Continuity Adj. Chi-Square	3.8581	0.0495
Likelihood Ratio Chi-Square	4.3338	0.0374
Mantel-Haenszel Chi-Square	4.2973	0.0382

Fisher's Exact Test	
Left-sided Pr <= F	0.0244
Right-sided Pr >= F	0.9857
Table Probability (P)	0.0102
Two-sided Pr <= P	0.0390

Analysis of Maximum Likelihood Estimates				
			Type 3 Tests	
**Parameter**	**Parameter Estimate**	**Standard Error**	**Chi-Square**	**p-value**
Group allocation Test	-0.456	0.223	4.2061	0.0403

		Confidence Interval	
**Parameter**	**Hazard Ratio**	**lower**	**upper**
Group allocation Test	0.634	0.410	0.980

Analysis of Maximum Likelihood Estimates				
			Type 3 Tests	
**Parameter**	**Parameter Estimate**	**Standard Error**	**Chi-Square**	**p-value**
Age [years]	0.108	0.018	36.2773	<.0001
Pack year history	0.015	0.004	16.1504	<.0001
Gender Male	0.154	0.228	0.4550	0.5000
Smoking status Ex-smoker	-0.443	0.225	3.8986	0.0483
Group allocation Test	-0.472	0.223	4.4963	0.0340

		Confidence Interval	
**Parameter**	**Hazard Ratio**	**lower**	**upper**
Age [years]	1.114	1.076	1.154
Pack year history	1.016	1.008	1.023
Gender Male	1.166	0.746	1.822
Smoking status Ex-smoker	0.642	0.413	0.997
Group allocation Test	0.624	0.403	0.965

Supplementary Data Pack S3:
Secondary Outcome Analysis 1: Lung Cancer Mortality Two Years After Randomisation

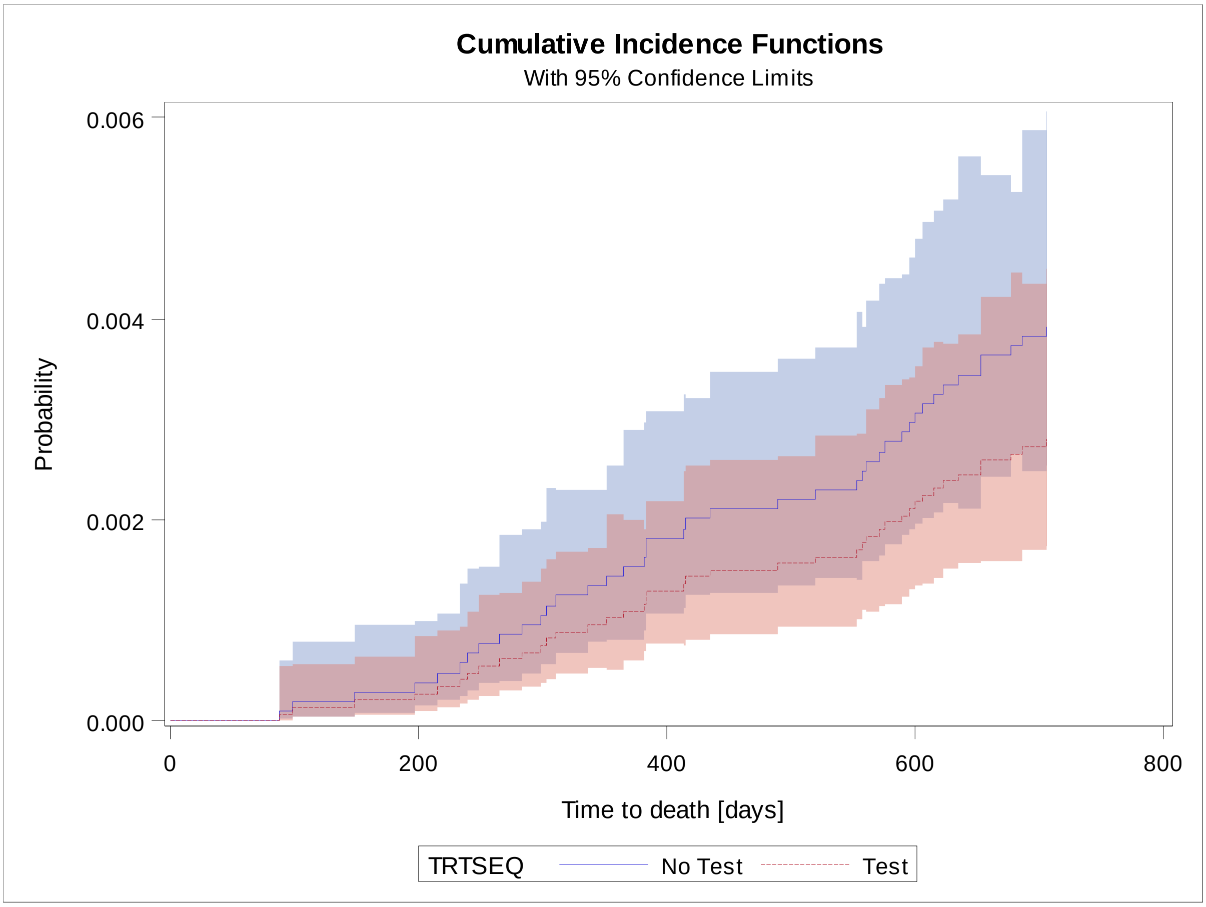

Lung cancer death reported in the 2 years since randomisation?

	No Test		Test		Total	
**Variable**	**N**	**(%)**	**N**	**(%)**	**N**	**(%)**
No	6097	(99.6%)	6070	(99.7%)	12167	(99.7%)
Yes	24	(0.4%)	17	(0.3%)	41	(0.3%)
Total	6121	(100.0%)	6087	(100.0%)	12208	(100.0%)

Chi-Square Statistics

**Statistic**	**Value**	**p-Value**
Chi-Square	1.1604	0.2814
Continuity Adj. Chi-Square	0.8478	0.3572
Likelihood Ratio Chi-Square	1.1662	0.2802
Mantel-Haenszel Chi-Square	1.1603	0.2814

Fisher's Exact Test

Left-sided Pr <= F	0.1787
Right-sided Pr >= F	0.8916
Table Probability (P)	0.0703
Two-sided Pr <= P	0.3482

Analysis of Maximum Likelihood Estimates				
			Type 3 Tests	
Parameter	Parameter Estimate	Standard Error	Chi-Square	p-value
Group allocation Test	-0.340	0.317	1.1509	0.2834

		Confidence Interval	
Parameter	Hazard Ratio	lower	upper
Group allocation Test	0.712	0.382	1.325

Analysis of Maximum Likelihood Estimates					
			Type 3 Tests		
Parameter	Parameter Estimate	Standard Error	Chi-Square	p-value	adjusted p-value
Age [years]	0.127	0.026	23.1273	<.0001	<.0001
Pack year history	0.016	0.004	21.5281	<.0001	<.0001
Random Factor Practice			2.5531		0.3744
Gender Male	0.121	0.329	0.1358	0.7125	0.7122
Smoking status Ex-smoker	-0.924	0.340	7.3666	0.0066	0.0066
Group allocation Test	-0.330	0.318	1.0805	0.2986	0.2985

		Confidence Interval	
Parameter	Hazard Ratio	lower	upper
Age [years]	1.135	1.078	1.195
Pack year history	1.016	1.009	1.023
Random Factor Practice			
Gender Male	1.129	0.592	2.154
Smoking status Ex-smoker	0.397	0.204	0.774
Group allocation Test	0.719	0.386	1.340

Secondary Outcome Analysis 2: Cancer Mortality Two Years After Randomisation

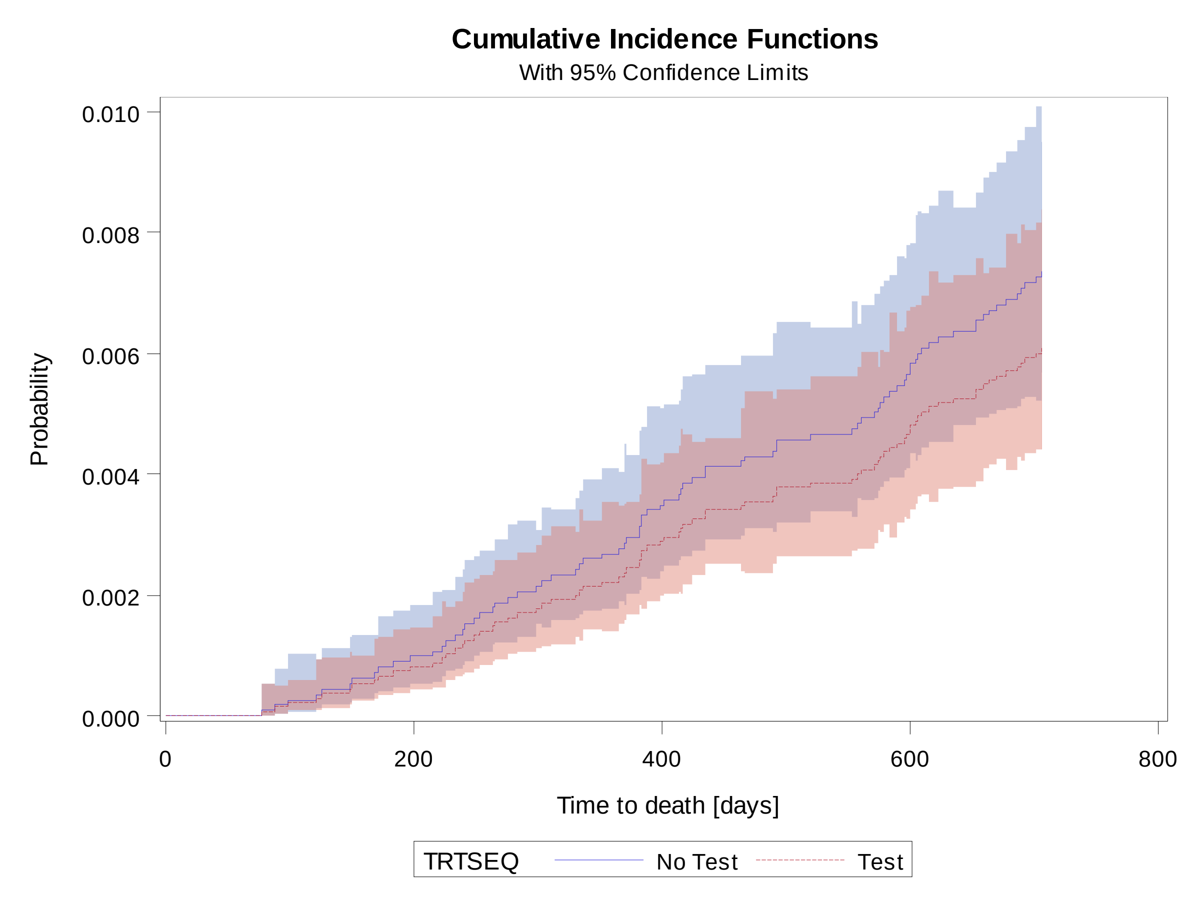

Any cancer death reported in the 2 years since randomisation?

	No Test		Test		Total	
**Variable**	**N**	**(%)**	**N**	**(%)**	**N**	**(%)**
No	6076	(99.3%)	6050	(99.4%)	12126	(99.3%)
Yes	45	(0.7%)	37	(0.6%)	82	(0.7%)
Total	6121	(100.0%)	6087	(100.0%)	12208	(100.0%)

Chi-Square Statistics

Statistic	Value	p-Value
Chi-Square	0.7415	0.3892
Continuity Adj. Chi-Square	0.5630	0.4531
Likelihood Ratio Chi-Square	0.7428	0.3888
Mantel-Haenszel Chi-Square	0.7415	0.3892

Fisher's Exact Test

Left-sided Pr <= F	0.2266
Right-sided Pr >= F	0.8344
Table Probability (P)	0.0611
Two-sided Pr <= P	0.4383

Analysis of Maximum Likelihood Estimates				
			Type 3 Tests	
Parameter	Parameter Estimate	Standard Error	Chi-Square	p-value
Group allocation Test	-0.191	0.222	0.7400	0.3897

		Confidence Interval	
Parameter	Hazard Ratio	lower	upper
Group allocation Test	0.826	0.535	1.276

Analysis of Maximum Likelihood Estimates				
			Type 3 Tests	
Parameter	Parameter Estimate	Standard Error	Chi-Square	p-value
Age [years]	0.098	0.018	30.4475	<.0001
Pack year history	0.011	0.004	10.0361	0.0015
Gender Male	0.286	0.232	1.5111	0.2190
Smoking status Ex-smoker	-0.859	0.240	12.7782	0.0004
Group allocation Test	-0.191	0.222	0.7366	0.3907

		Confidence Interval	
Parameter	Hazard Ratio	lower	upper
Age [years]	1.103	1.065	1.142
Pack year history	1.011	1.004	1.019
Gender Male	1.331	0.844	2.099
Smoking status Ex-smoker	0.424	0.265	0.679
Group allocation Test	0.827	0.535	1.277

Secondary Outcome Analysis 3: All-Cause Mortality Two Years After Randomisation

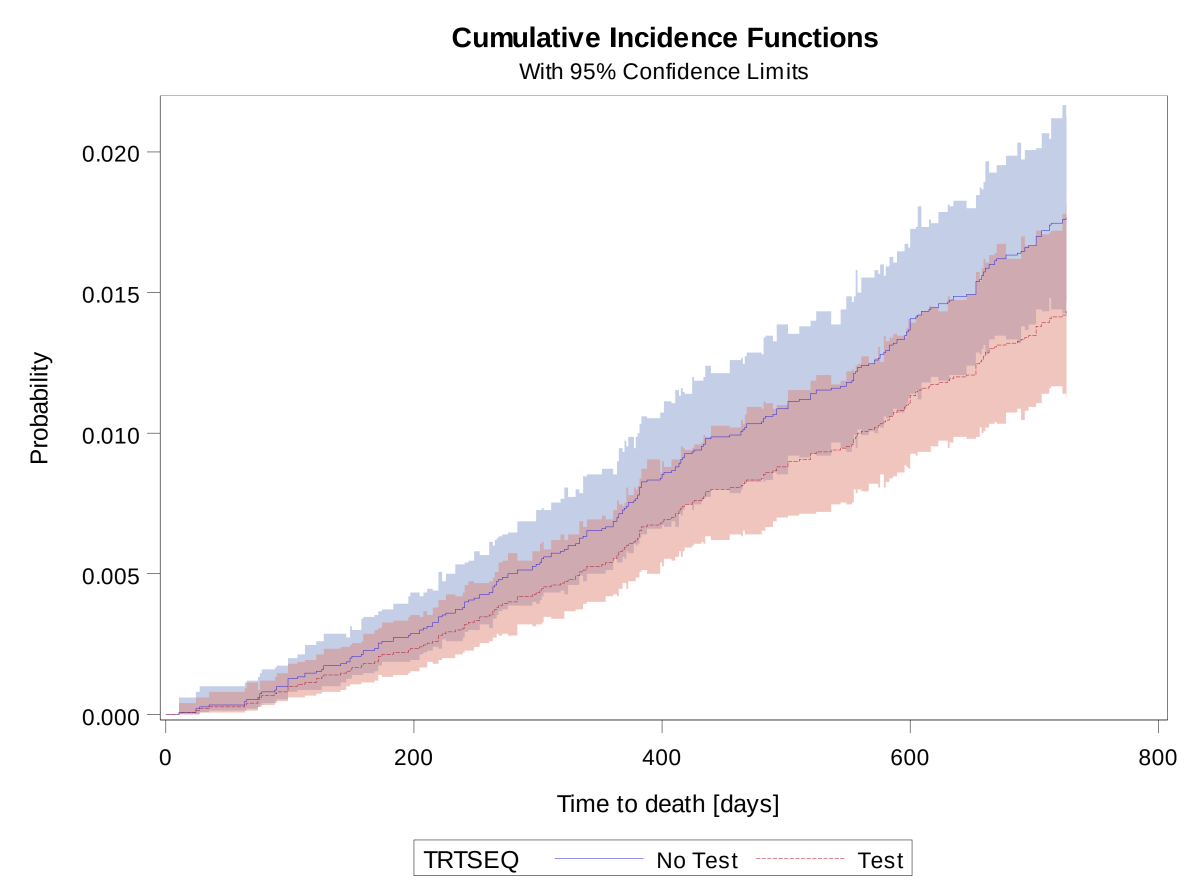

Any cause death reported in the 2 years since randomisation?

	No Test		Test		Total	
**Variable**	**N**	**(%)**	**N**	**(%)**	**N**	**(%)**
No	6013	(98.2%)	6000	(98.6%)	12013	(98.4%)
Yes	108	(1.8%)	87	(1.4%)	195	(1.6%)
Total	6121	(100.0%)	6087	(100.0%)	12208	(100.0%)

Chi-Square Statistics		
**Statistic**	**Value**	**p-Value**
Chi-Square	2.1809	0.1397
Continuity Adj. Chi-Square	1.9729	0.1601
Likelihood Ratio Chi-Square	2.1853	0.1393
Mantel-Haenszel Chi-Square	2.1808	0.1397

Fisher's Exact Test	
Left-sided Pr <= F	0.0800
Right-sided Pr >= F	0.9394
Table Probability (P)	0.0194
Two-sided Pr <= P	0.1489

Analysis of Maximum Likelihood Estimates				
			Type 3 Tests	
Parameter	Parameter Estimate	Standard Error	Chi-Square	p-value
Group allocation Test	-0.212	0.144	2.1735	0.1404

		Confidence Interval	
Parameter	Hazard Ratio	lower	upper
Group allocation Test	0.809	0.610	1.072

Analysis of Maximum Likelihood Estimates					
			Type 3 Tests		
Parameter	Parameter Estimate	Standard Error	Chi-Square	p-value	adjusted p-value
Age [years]	0.070	0.011	38.4582	<.0001	<.0001
Pack year history	0.010	0.003	14.9175	0.0001	0.0001
Random Factor Practice			14.3637		0.3422
Gender Male	0.486	0.154	10.0249	0.0015	0.0015
Smoking status Ex-smoker	-0.679	0.153	19.6351	<.0001	<.0001
Group allocation Test	-0.212	0.144	2.1549	0.1421	0.1420

		Confidence Interval	
Parameter	Hazard Ratio	lower	upper
Age [years]	1.072	1.049	1.096
Pack year history	1.010	1.005	1.015
Random Factor Practice			
Gender Male	1.626	1.203	2.197
Smoking status Ex-smoker	0.507	0.375	0.685
Group allocation Test	0.809	0.610	1.073
