# Supplementary material for: Earlier diagnosis of lung cancer in a randomised trial of an autoantibody blood test followed by imaging
Source: Eur Respir J. 2021 Jan 14;57(1):2000670. doi: 10.1183/13993003.00670-2020 (PMC7806972; doi:10.1183/13993003.00670-2020)
Supplement: Supplementary file 3 [file ERJ-00670-2020.Shareable.pdf]

SHAREABLE PDF

# Earlier diagnosis of lung cancer in a randomised trial of an autoantibody blood test followed by imaging

Frank M. Sullivan[1], Frances S. Mair[2], William Anderson[3], Pauline Armory[4], Andrew Briggs[5], Cindy Chew[6], Alistair Dorward[7], John Haughney[8], Fiona Hogarth[4], Denise Kendrick[9], Roberta Littleford[10], Alex McConnachie[2], Colin McCowan[1], Nicola McMeekin[2], Manish Patel[11], Petra Rauchhaus[4], Lewis Ritchie[12], Chris Robertson[13], John Robertson[9], Jose Robles-Zurita[2], Joseph Sarvesvaran[7], Herbert Sewell[14], Michael Sproule[15], Thomas Taylor[16], Agnes Tello[1], Shaun Treweek[17], Kavita Vedhara[9] and Stuart Schembri[3] on behalf of the Early Diagnosis of Lung Cancer Scotland (ECLS) Team

**Affiliations**: [1]School of Medicine, University of St Andrews, St Andrews, UK. [2]Institute of Health and Wellbeing, University of Glasgow, Glasgow, UK. [3]Respiratory Medicine, NHS Tayside, Dundee, UK. [4]Tayside Clinical Trials Unit, University of Dundee, Dundee, UK. [5]Dept of Health Services Research and Policy, London School of Hygiene and Tropical Medicine, London, UK. [6]Radiology, NHS Lanarkshire, Bothwell, UK. [7]Respiratory Medicine, NHS Greater Glasgow and Clyde, Glasgow, UK. [8]General Practice, NHS Greater Glasgow and Clyde, Glasgow, UK. [9]School of Medicine, University of Nottingham, Nottingham, UK. [10]Centre for Clinical Research, University of Queensland, Saint Lucia, Australia. [11]Respiratory Medicine, NHS Lanarkshire, Bothwell, UK. [12]The Institute of Applied Health Sciences, University of Aberdeen, Aberdeen, UK. [13]Dept of Mathematics and Statistics, University of Strathclyde, Glasgow, UK. [14]School of Life Sciences, University of Nottingham, Nottingham, UK. [15]Radiology, NHS Greater Glasgow and Clyde, Glasgow, UK. [16]Radiology, NHS Tayside, Dundee, UK. [17]Health Services Research Unit, University of Aberdeen, Aberdeen, UK.

**Correspondence**: Frank M. Sullivan, School of Medicine, Medical and Biological Sciences Building, University of St Andrews, North Haugh, St Andrews, KY16 9TF, UK. E-mail: fms20@st-andrews.ac.uk

@ERSpublications
**A positive EarlyCDT-Lung test, followed by CT, significantly reduced the numbers of late-stage cancers: performance of the blood test should be assessed further in a screening study where all eligible participants in the intervention arm have a CT scan** https://bit.ly/32maUrB

**Cite this article as:** Sullivan FM, Mair FS, Anderson W, *et al*. Earlier diagnosis of lung cancer in a randomised trial of an autoantibody blood test followed by imaging. *Eur Respir J* 2021; 57: 2000670 [https://doi.org/10.1183/13993003.00670-2020].

This single-page version can be shared freely online.

ABSTRACT The EarlyCDT-Lung test is a high-specificity blood-based autoantibody biomarker that could contribute to predicting lung cancer risk. We report on the results of a phase IV biomarker evaluation of whether using the EarlyCDT-Lung test and any subsequent computed tomography (CT) scanning to identify those at high risk of lung cancer reduces the incidence of patients with stage III/IV/unspecified lung cancer at diagnosis compared with the standard clinical practice at the time the study began.

The Early Diagnosis of Lung Cancer Scotland (ECLS) trial was a randomised controlled trial of 12 208 participants at risk of developing lung cancer in Scotland in the UK. The intervention arm received the EarlyCDT-Lung test and, if test-positive, low-dose CT scanning 6-monthly for up to 2 years. EarlyCDT-Lung test-negative and control arm participants received standard clinical care. Outcomes were assessed at 2 years post-randomisation using validated data on cancer occurrence, cancer staging, mortality and comorbidities.

At 2 years, 127 lung cancers were detected in the study population (1.0%). In the intervention arm, 33 out of 56 (58.9%) lung cancers were diagnosed at stage III/IV compared with 52 out of 71 (73.2%) in the

Copyright ©ERS 2021. This version is distributed under the terms of the Creative Commons Attribution Non-Commercial Licence 4.0.

control arm. The hazard ratio for stage III/IV presentation was 0.64 (95% CI 0.41–0.99). There were nonsignificant differences in lung cancer and all-cause mortality after 2 years.

ECLS compared EarlyCDT-Lung plus CT screening to standard clinical care (symptomatic presentation) and was not designed to assess the incremental contribution of the EarlyCDT-Lung test. The observation of a stage shift towards earlier-stage lung cancer diagnosis merits further investigations to evaluate whether the EarlyCDT-Lung test adds anything to the emerging standard of low-dose CT.
